# Supplementary material for: Potential risk factors associated with seropositivity for Toxoplasma gondii among pregnant women and HIV infected individuals in Ethiopia: A systematic review and meta-analysis
Source: PLoS Negl Trop Dis. 2020 Dec 15;14(12):e0008944. doi: 10.1371/journal.pntd.0008944 (PMC7771857; doi:10.1371/journal.pntd.0008944)

Potential risk factors for *T. gondii* seropositivity in pregnant women of Ethiopia

1. History of abortion/ miscarraige (yes/no)

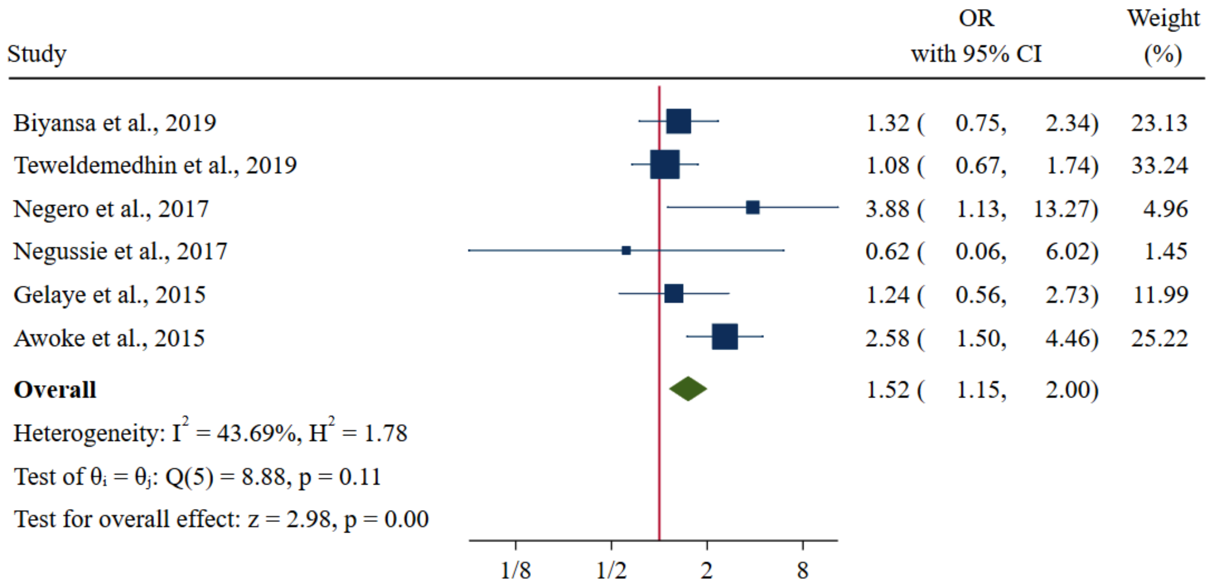

Fixed-effects inverse-variance model: history of abortion/miscarriage

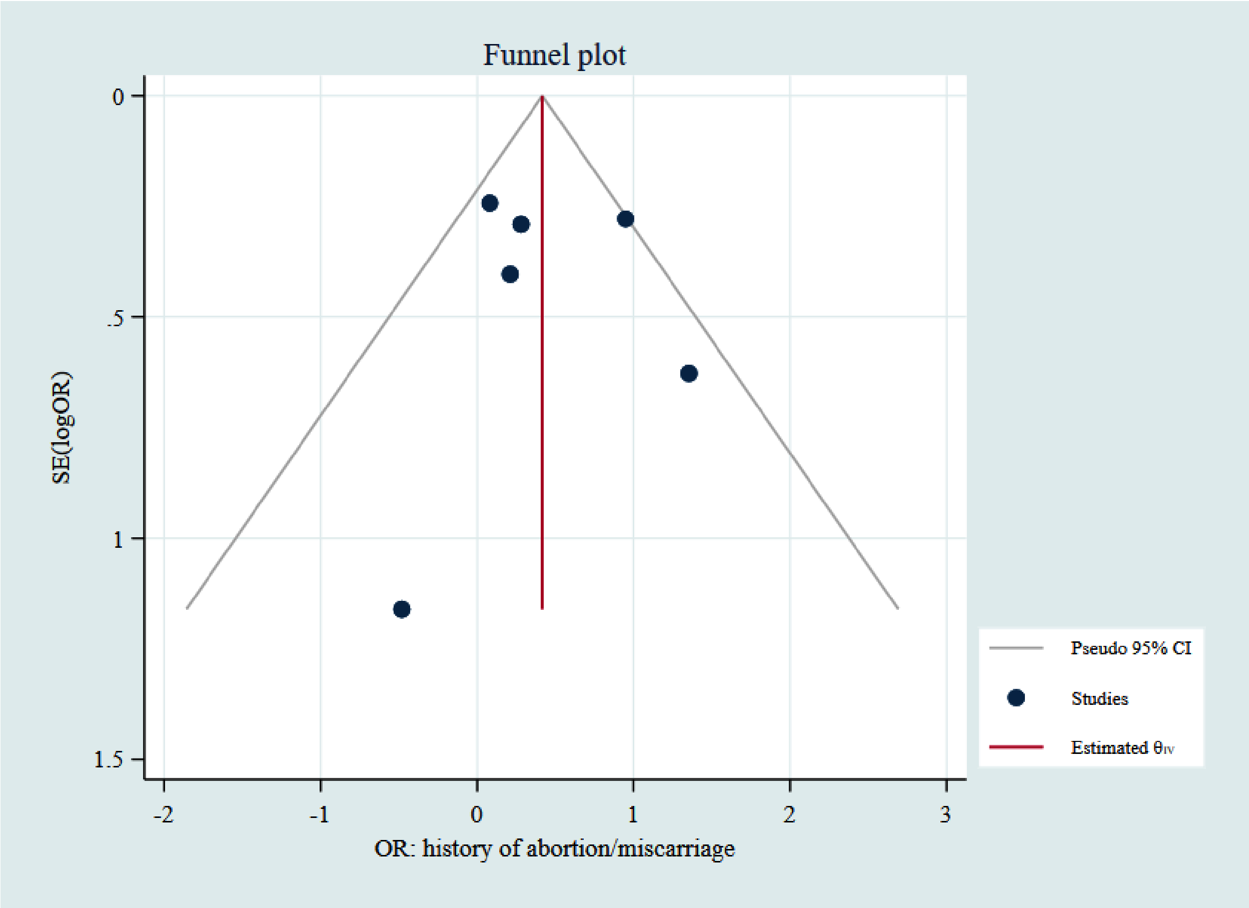

2. Age category (≥35 & 15-34)

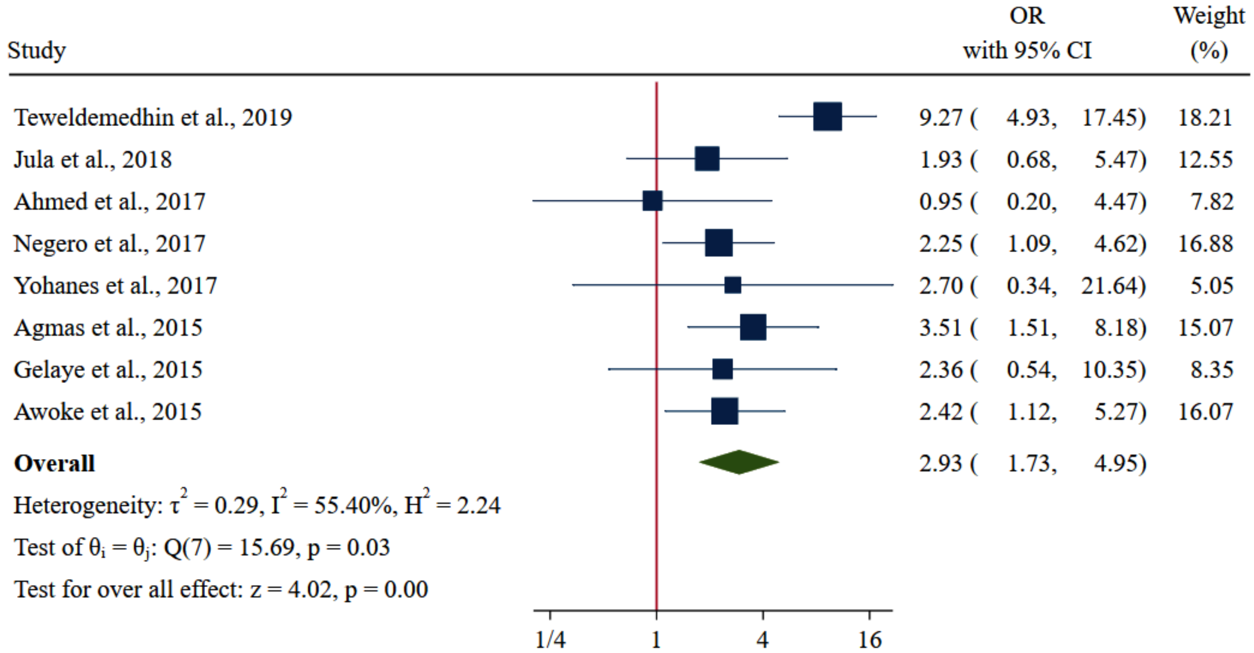

Random-effects DerSimonian-Laird model: age (≥35) vs age (15-34)

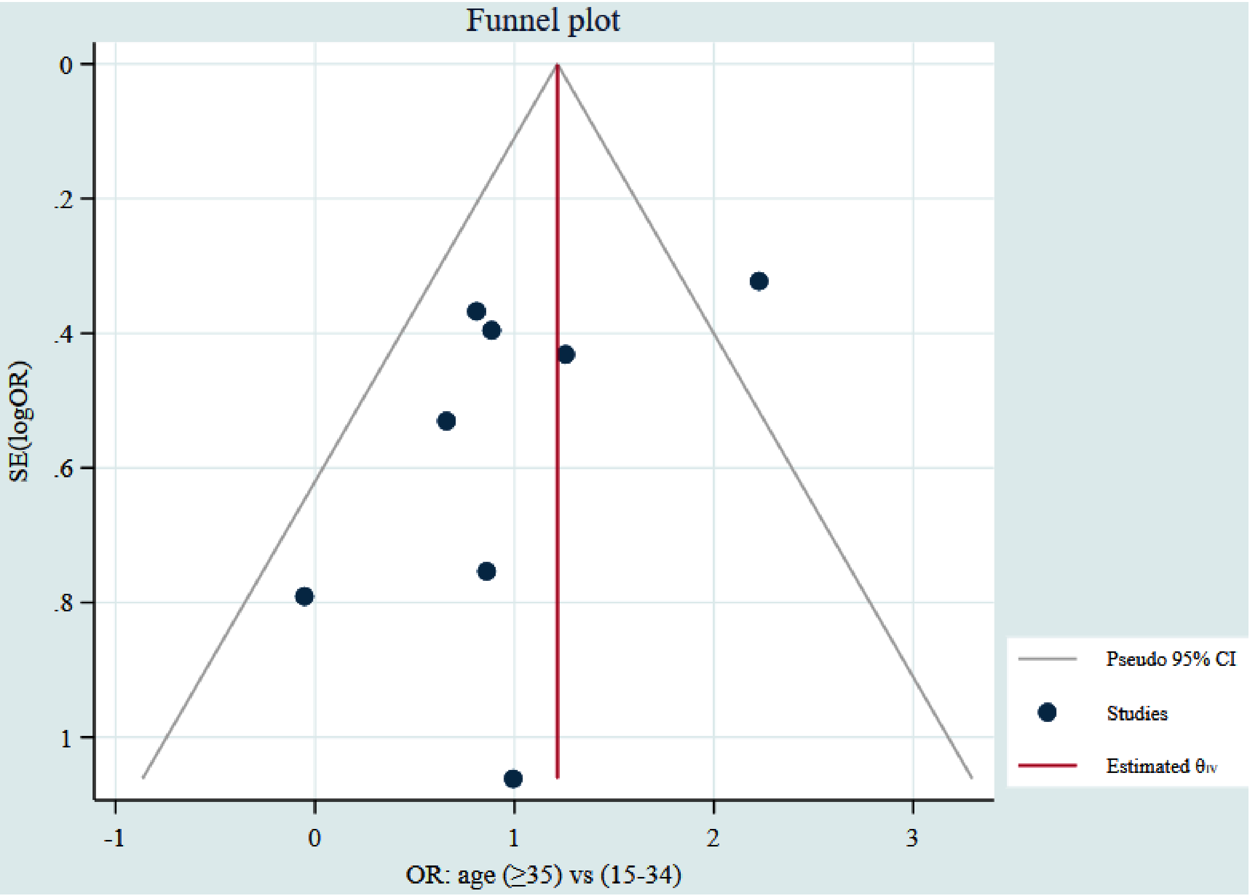

3. Blood transfusion experience (yes/no)

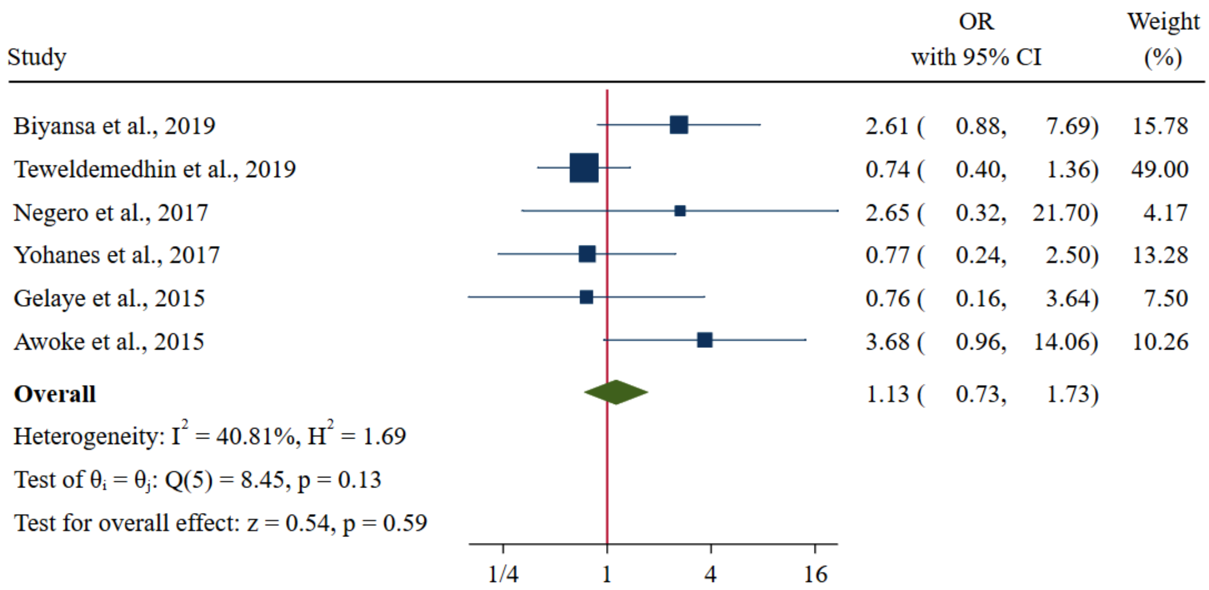

Fixed-effects inverse-variance model: blood transfusion experience

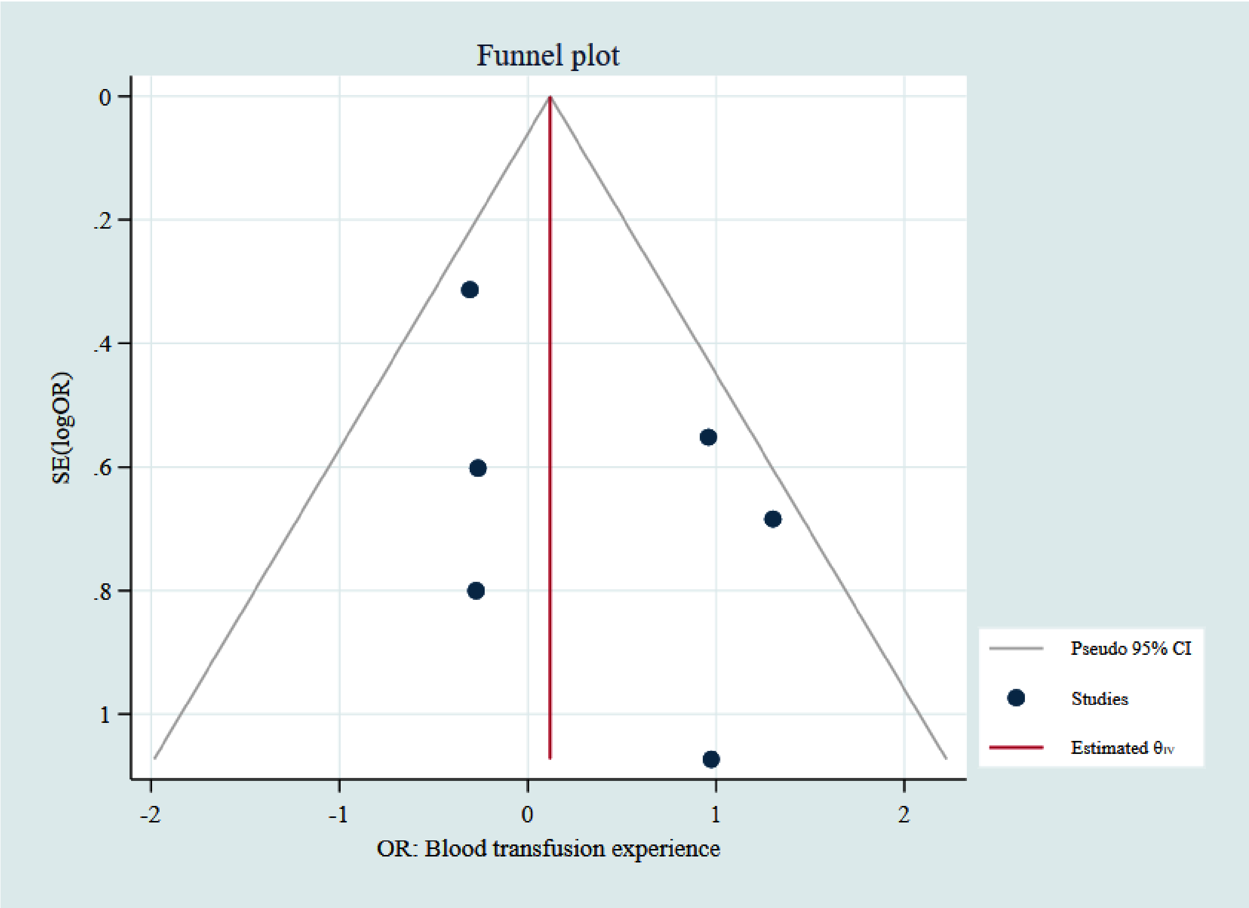

4. Contact with cat(yes/no)

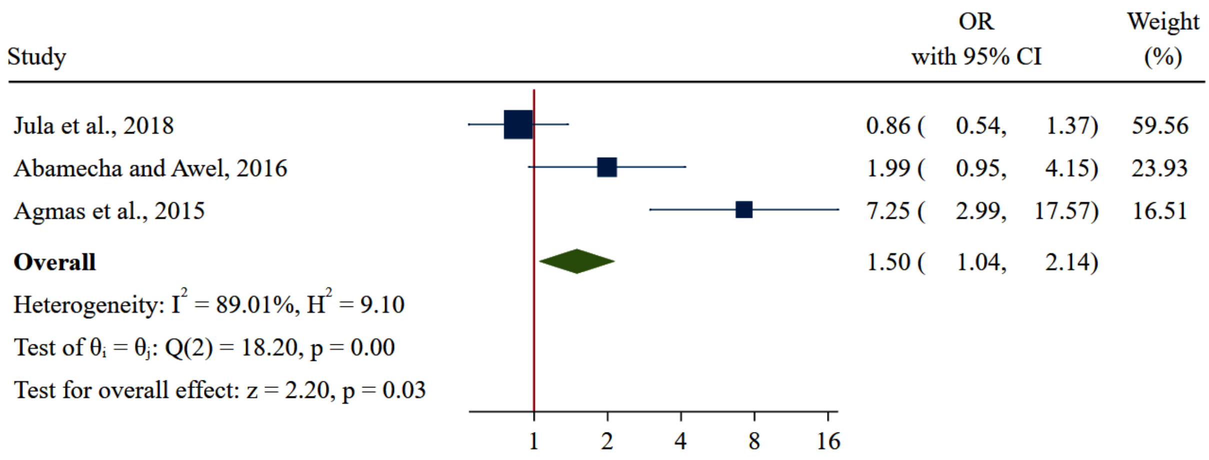

Fixed-effects inverse-variance model: contact with cat

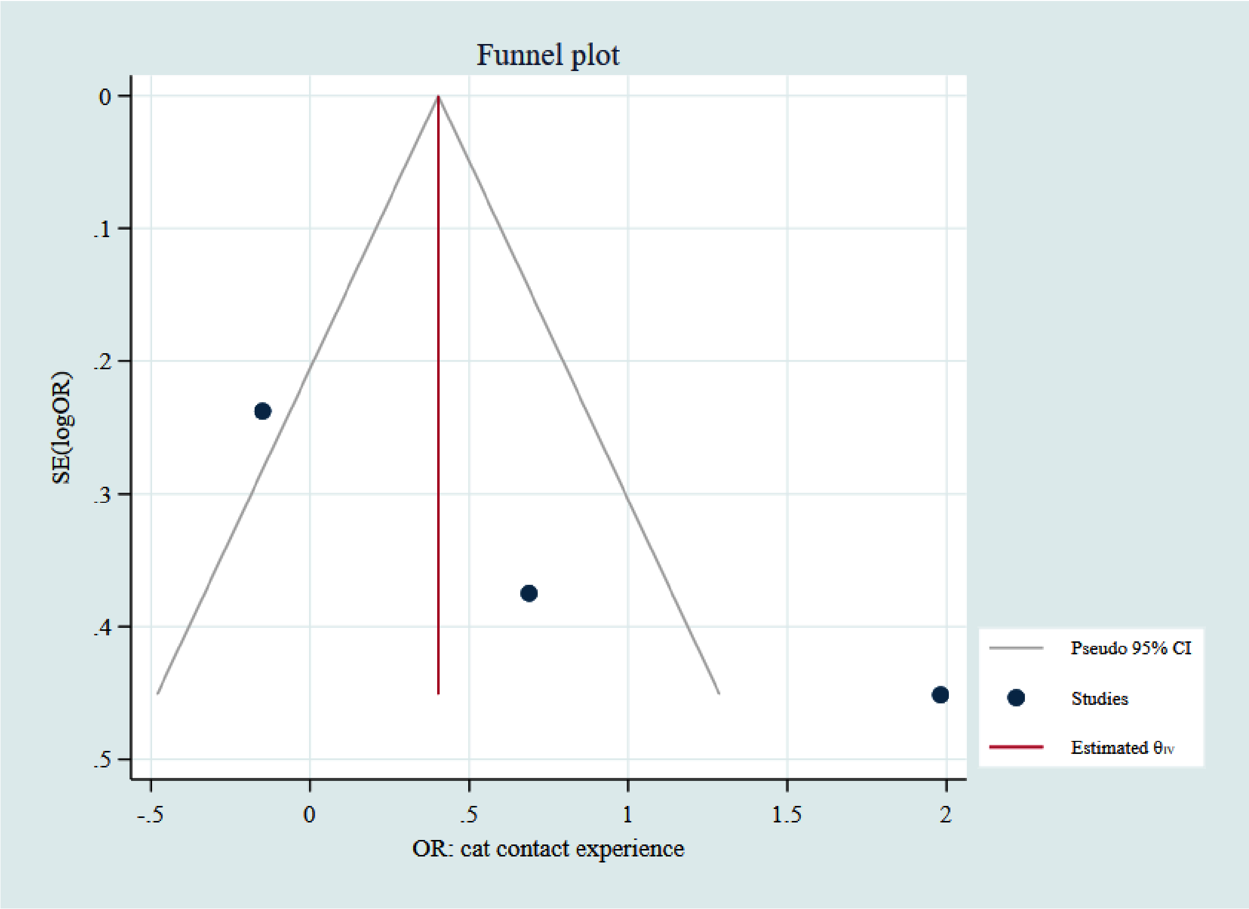

5. Cat presence/ownership(yes/no)

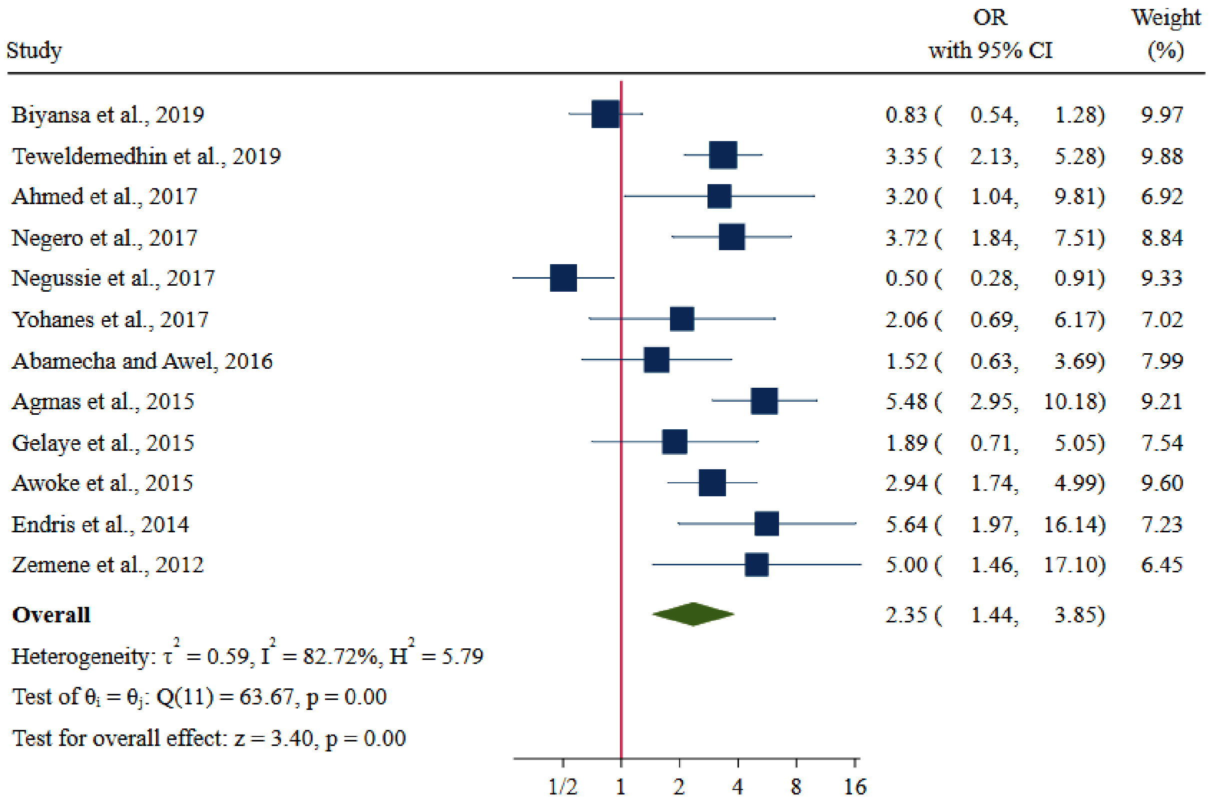

Random-effects DerSimonian-Laird model: Cat presence/ownership

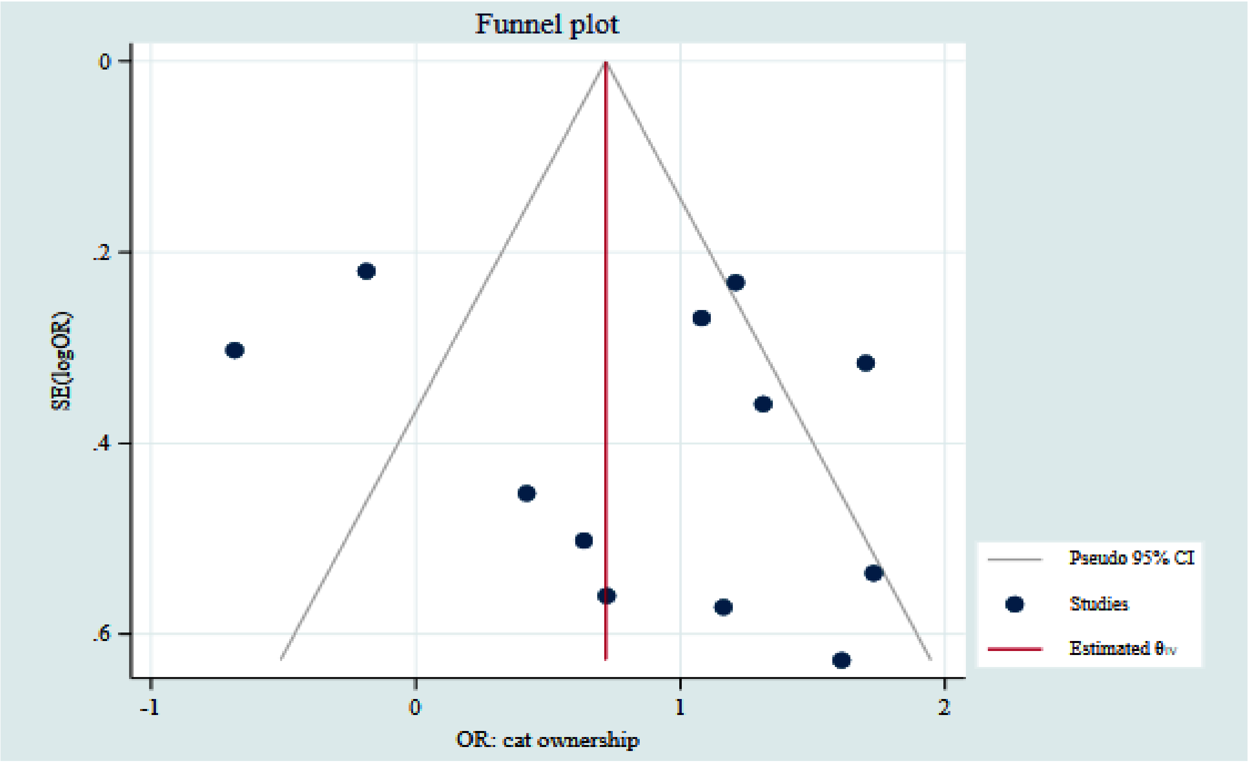

6. Contact with (garden) soil (yes/no)

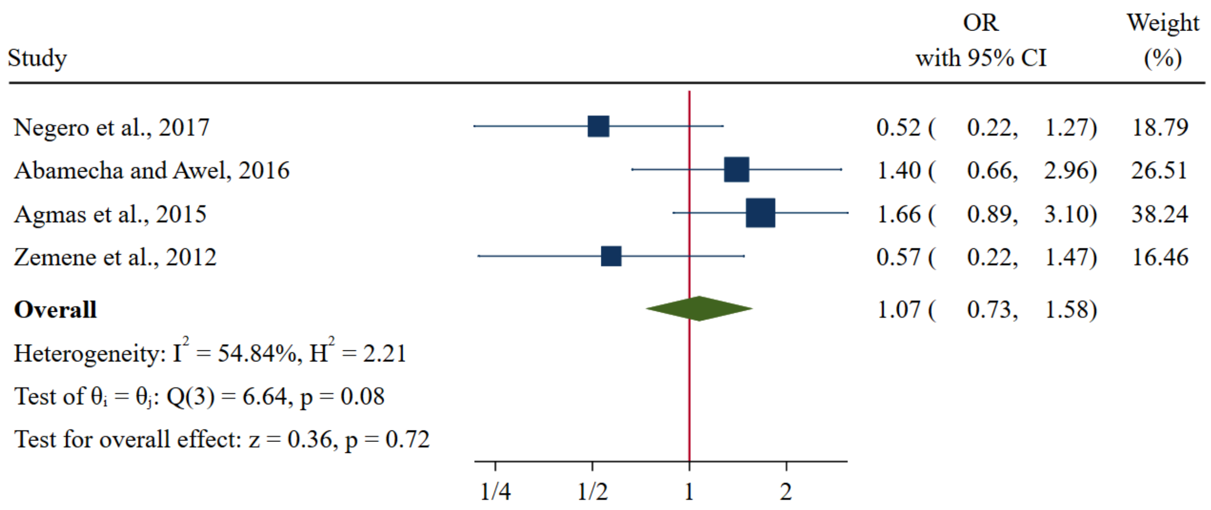

Fixed-effects inverse-variance model: contact with (garden) soil

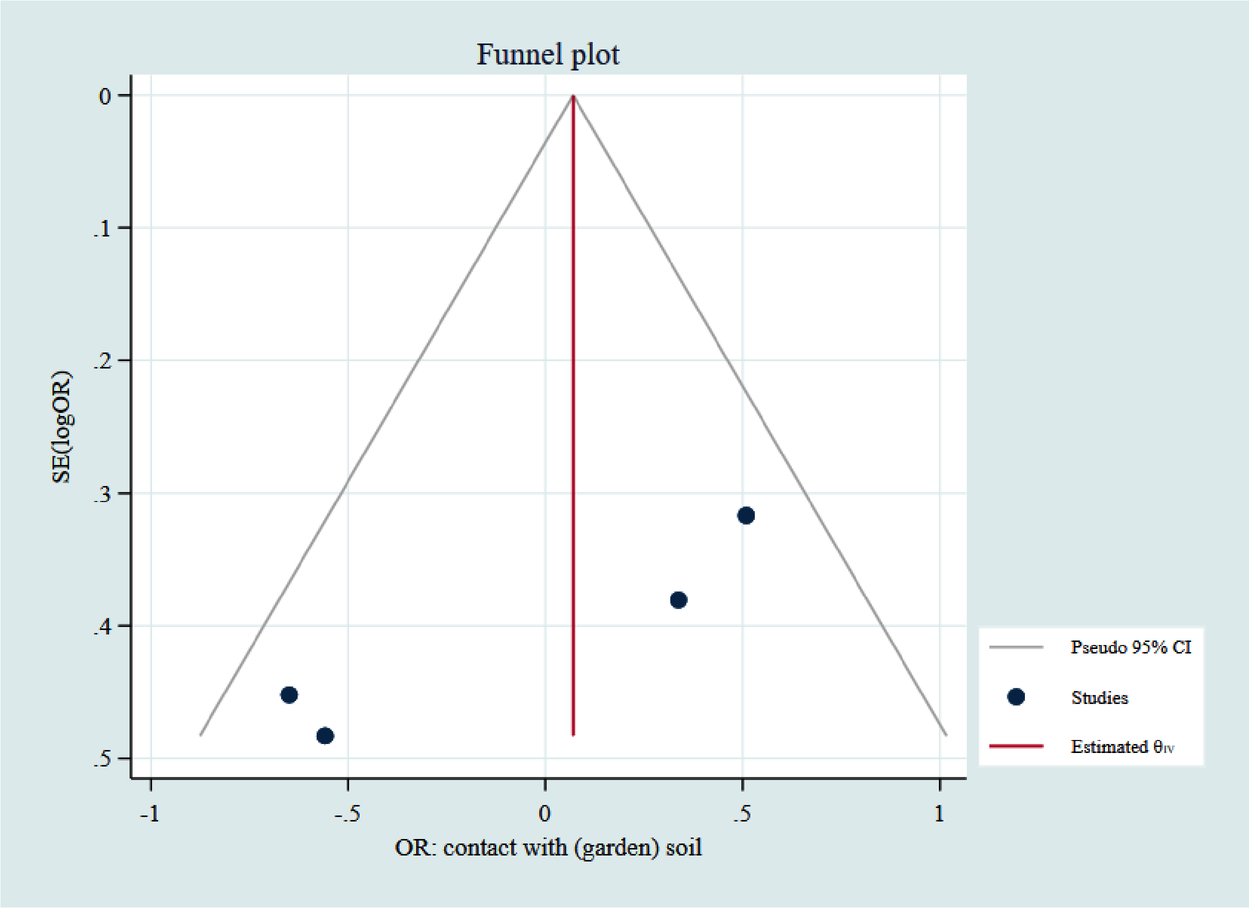

7. <u>Dog presence/ownership (yes/no)</u>

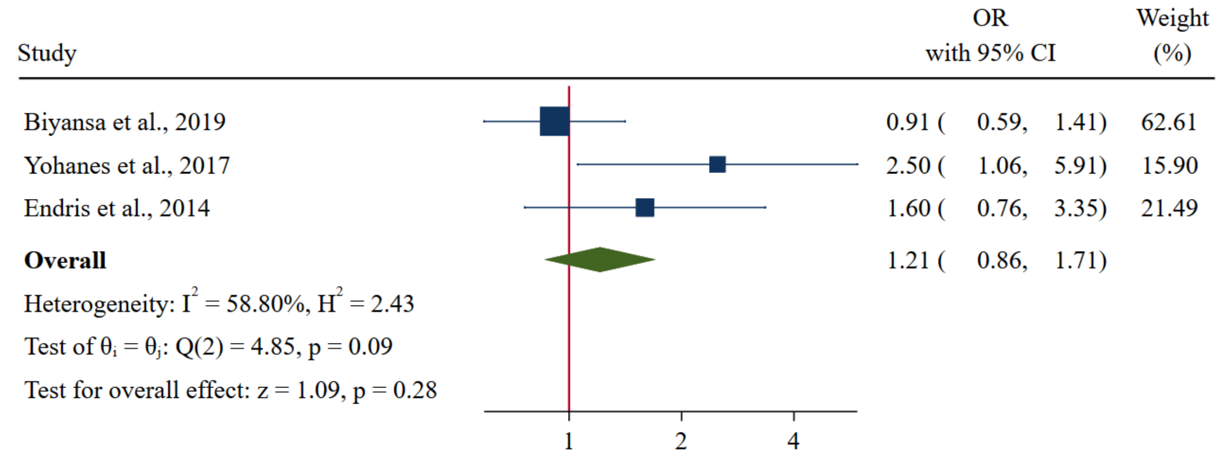

Fixed-effects inverse-variance model: presence of dogs

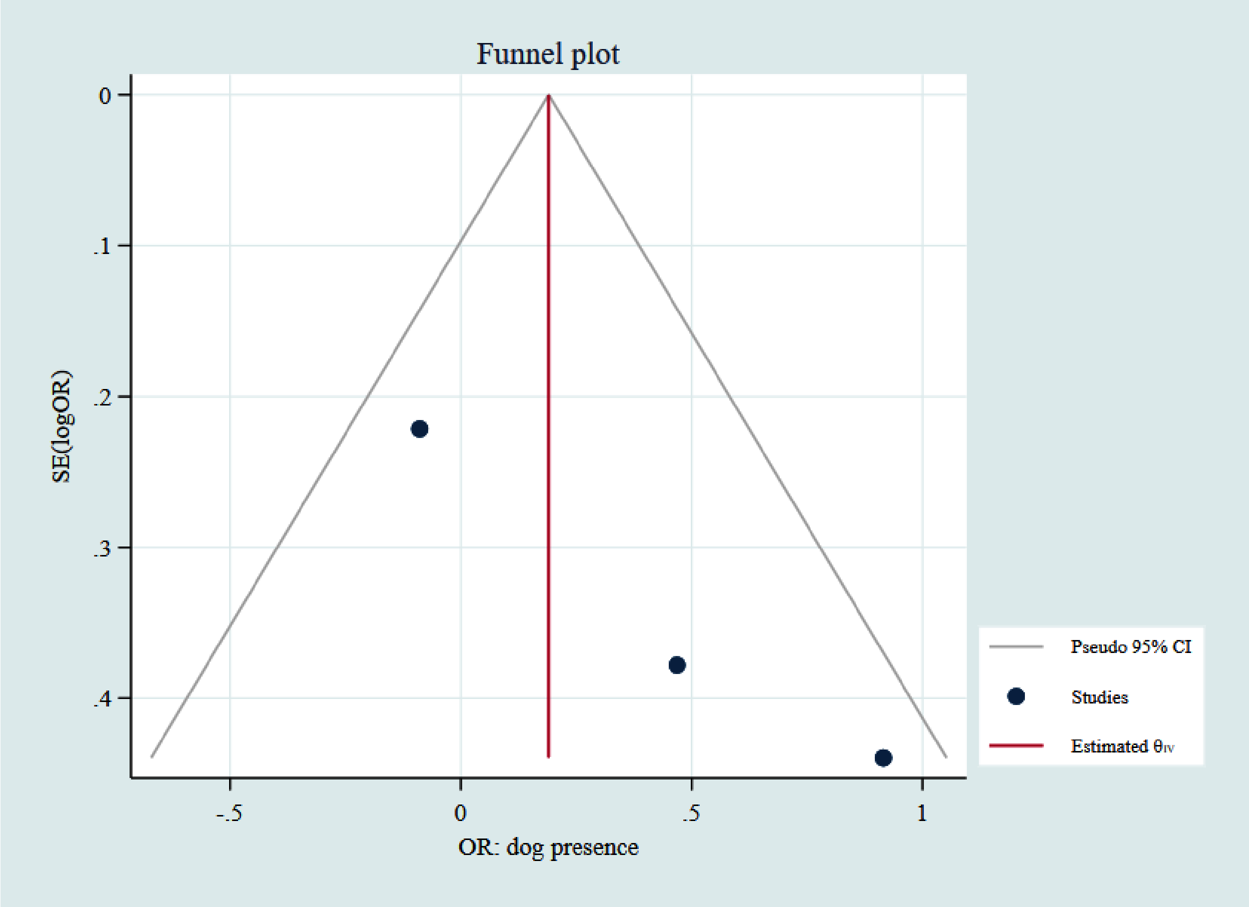

8. Educational level (illiterate/literate)

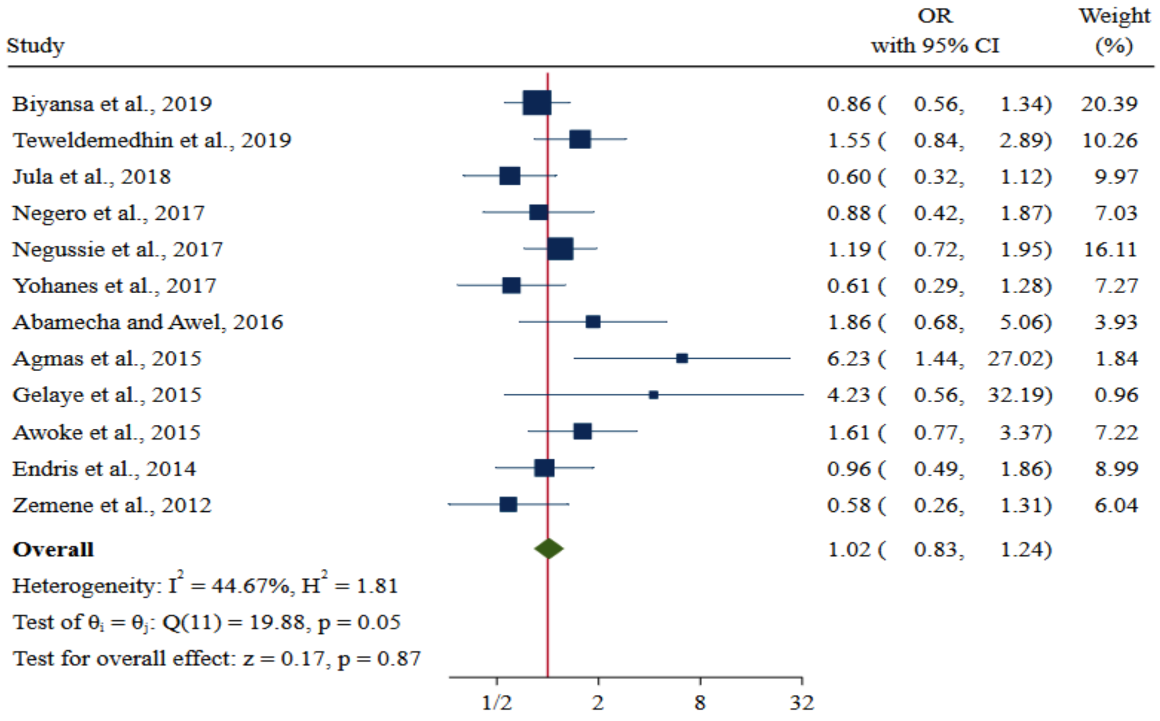

Fixed-effects inverse-variance model: Educational level

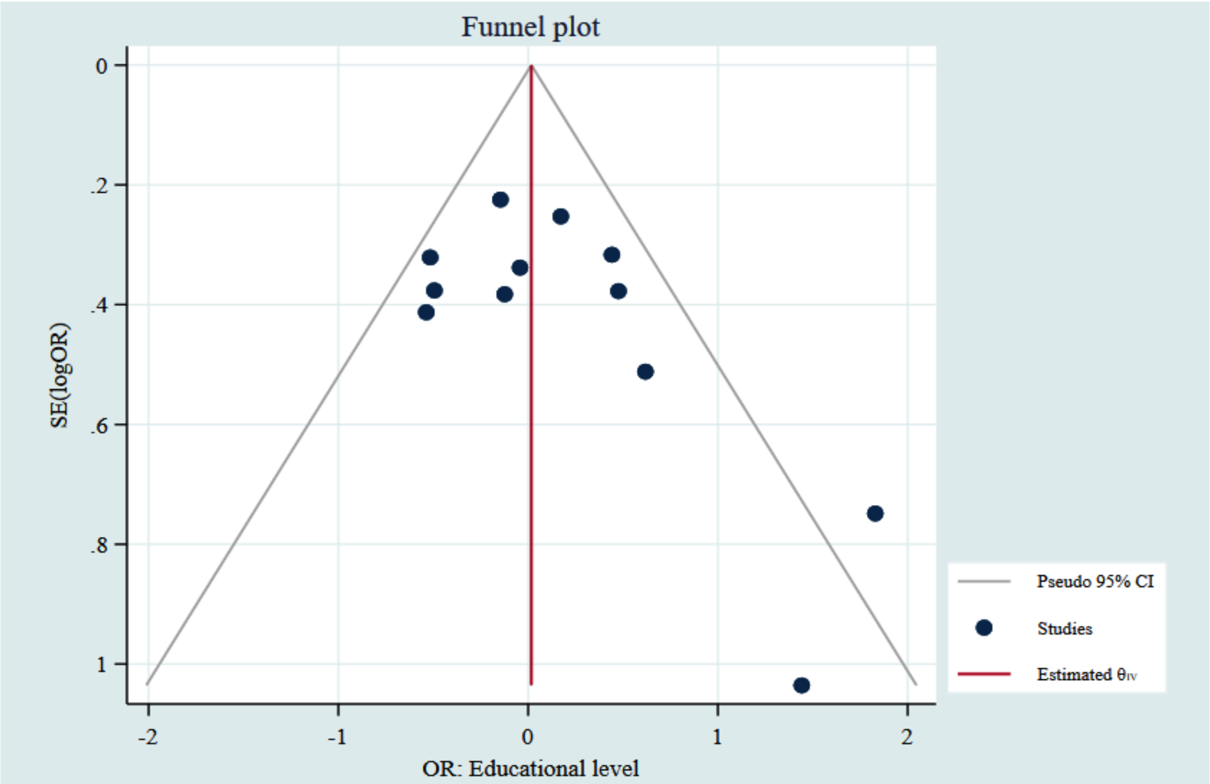

9. Farming or gardening activity (yes/no)

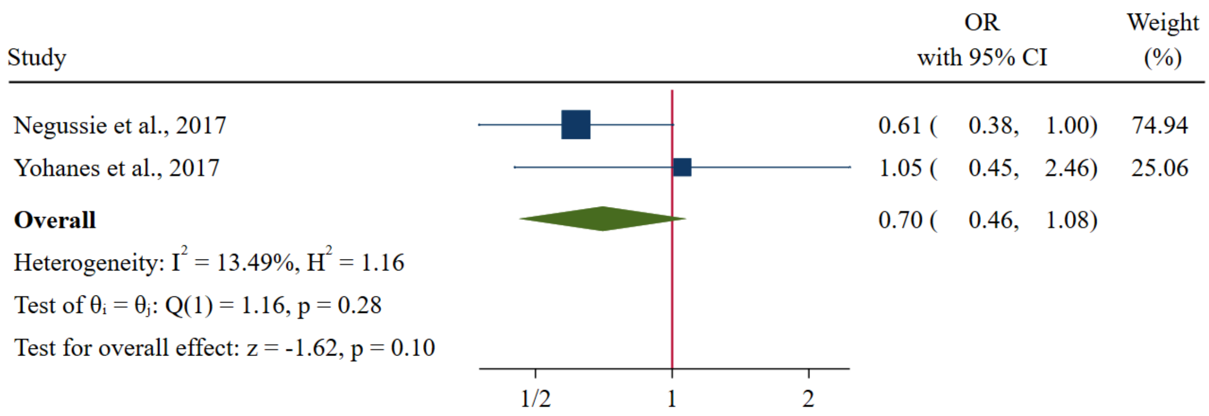

Fixed-effects inverse-variance model: farming/gardening activity

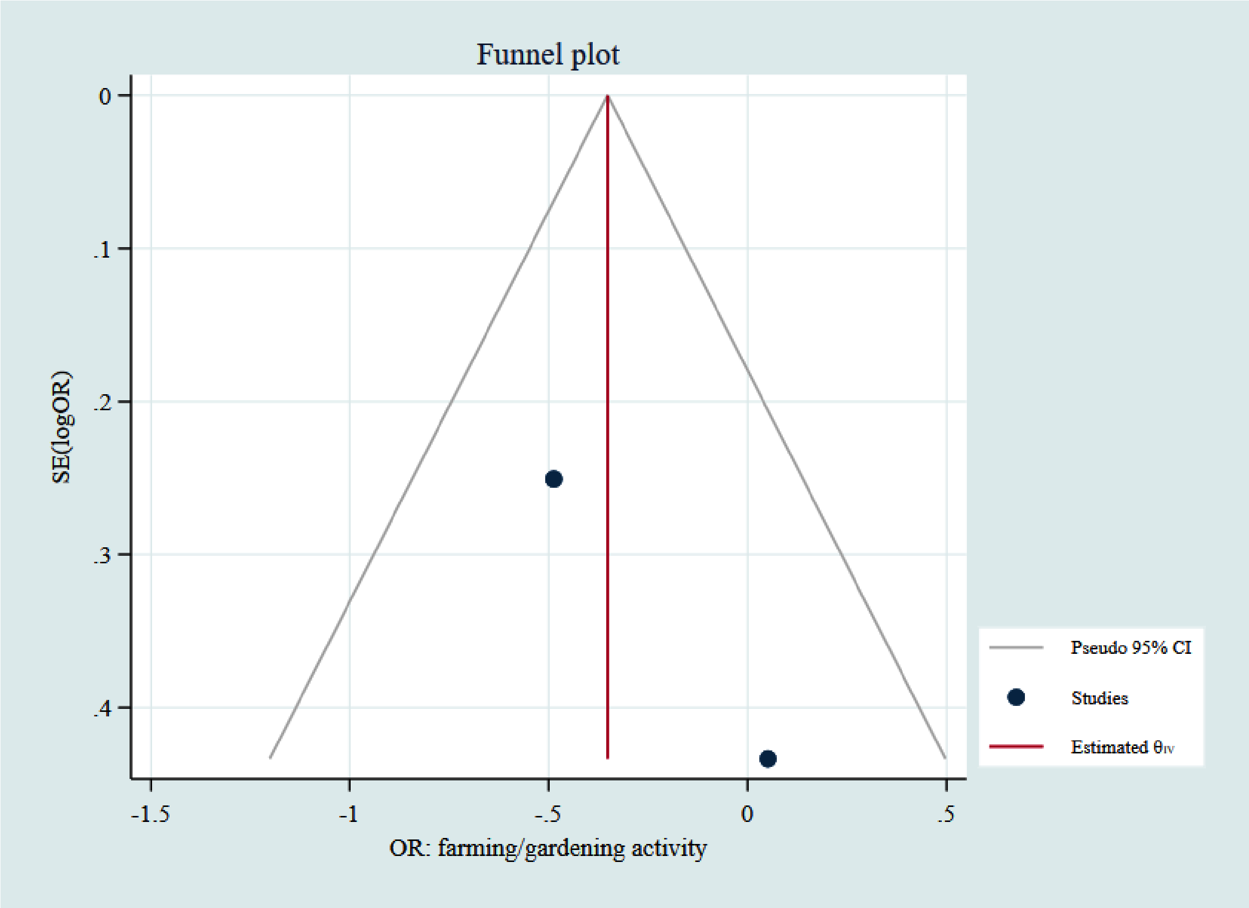

10. Hand washing habit after meat contact (regularly/ sometimes)

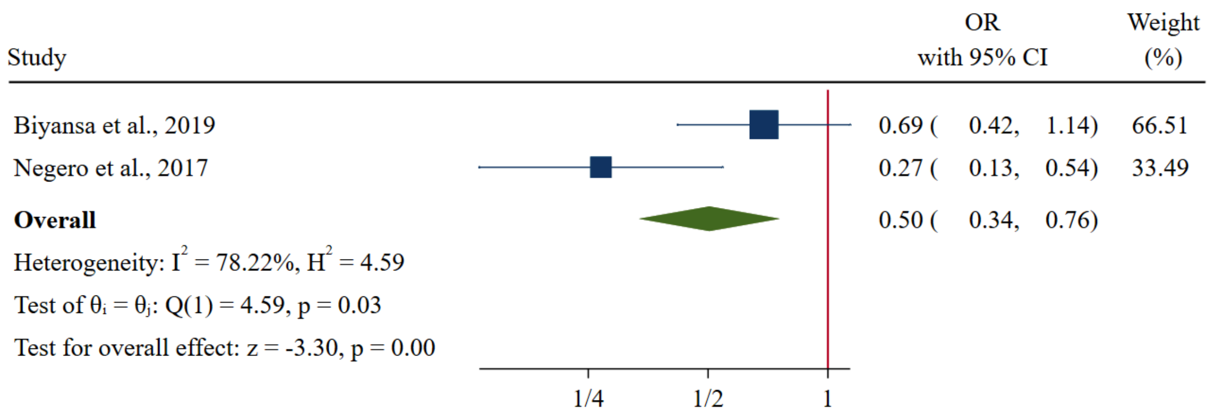

Fixed-effects inverse-variance model: handwashing habit after meat handling

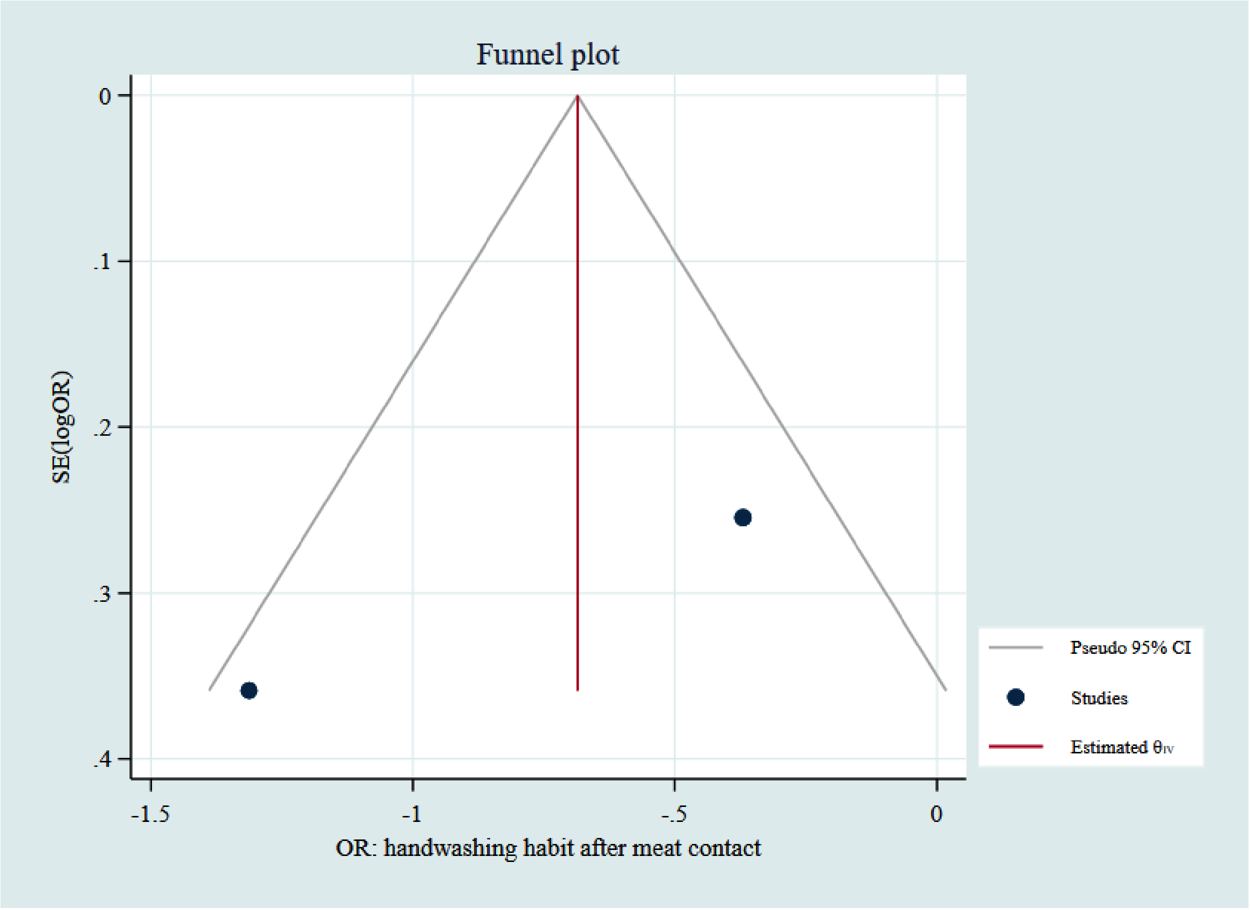

11. HIV status (positive/negative)

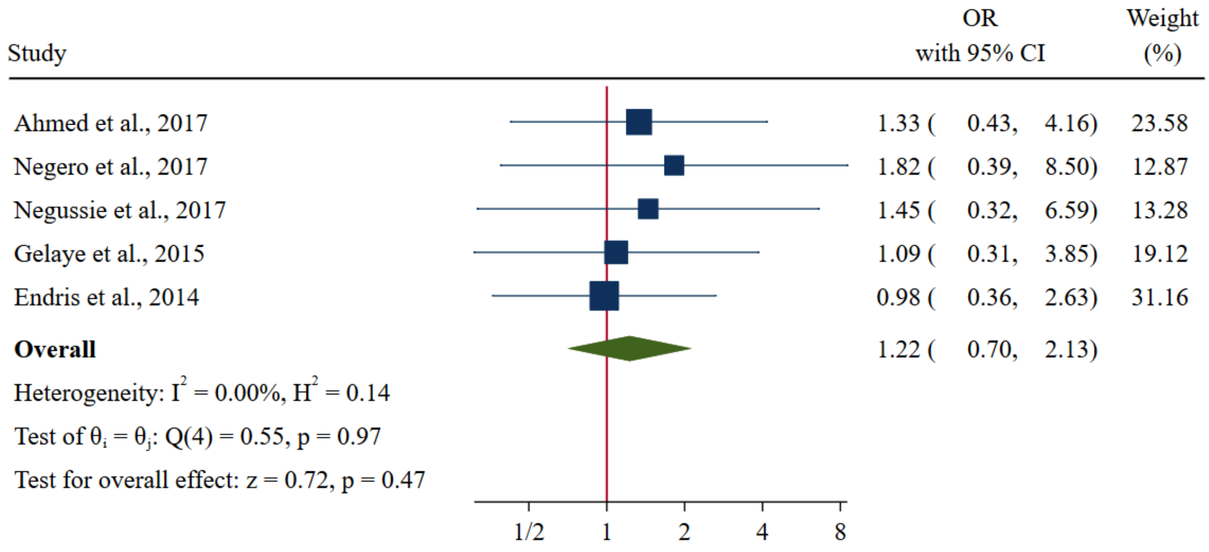

Fixed-effects inverse-variance model: HIV status

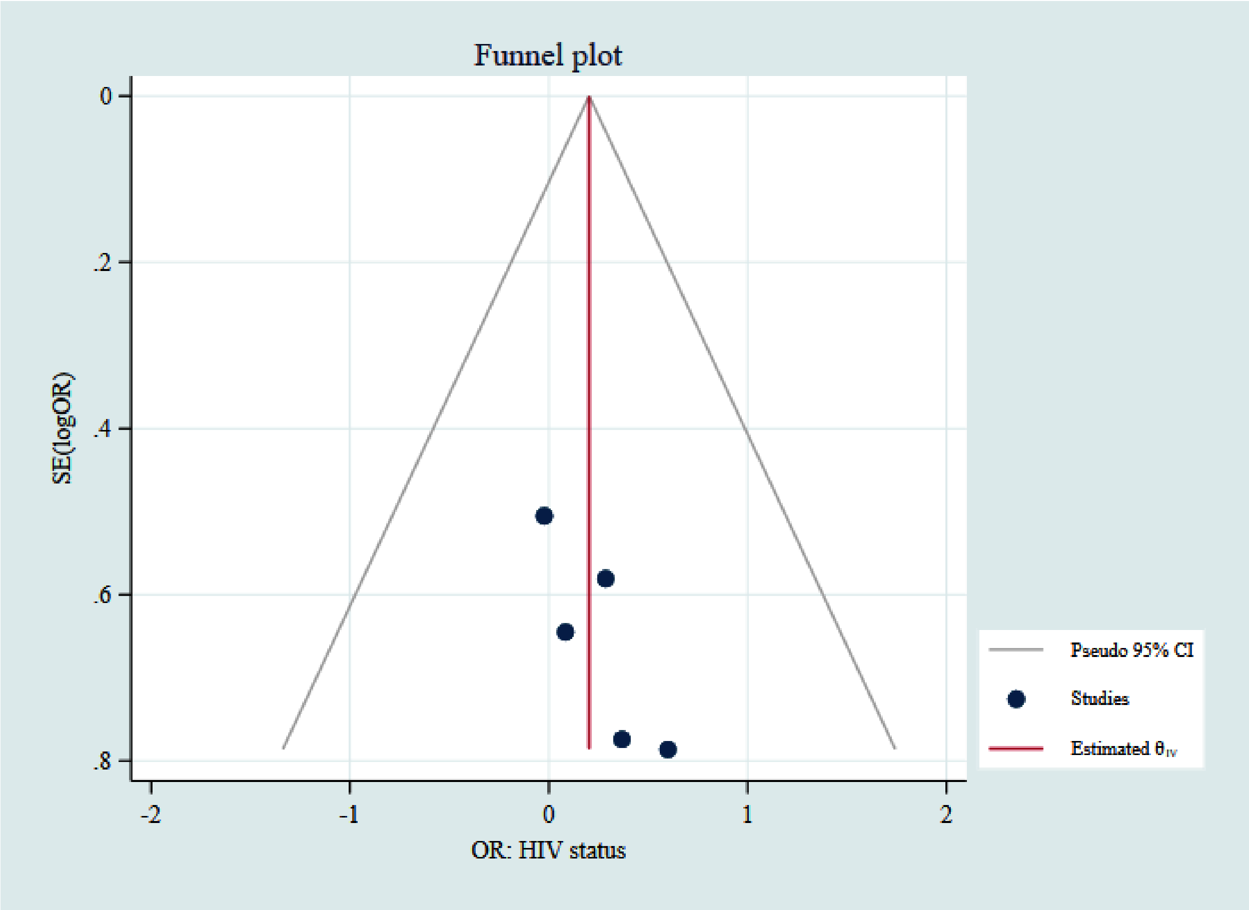

12. Knowledge on toxoplasmosis (yes/no)

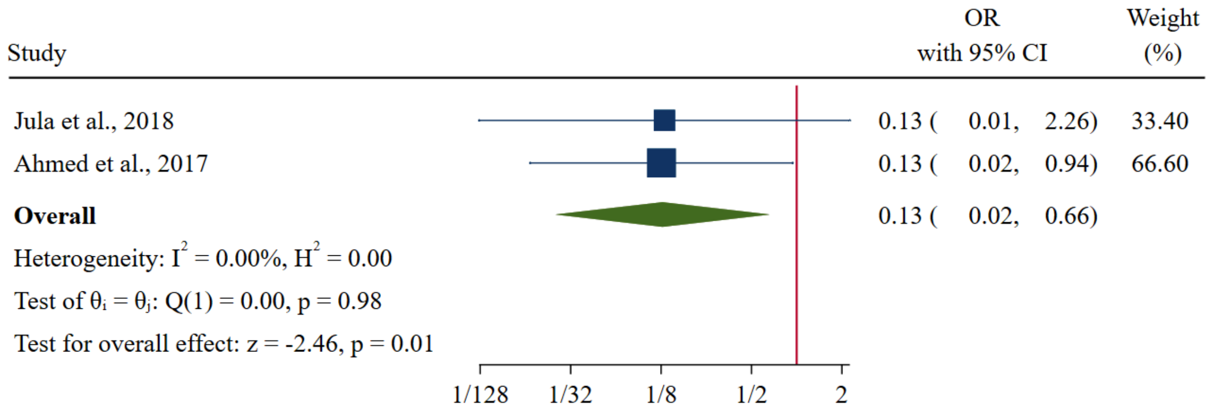

Fixed-effects inverse-variance mode: knowledge on toxoplasmosis

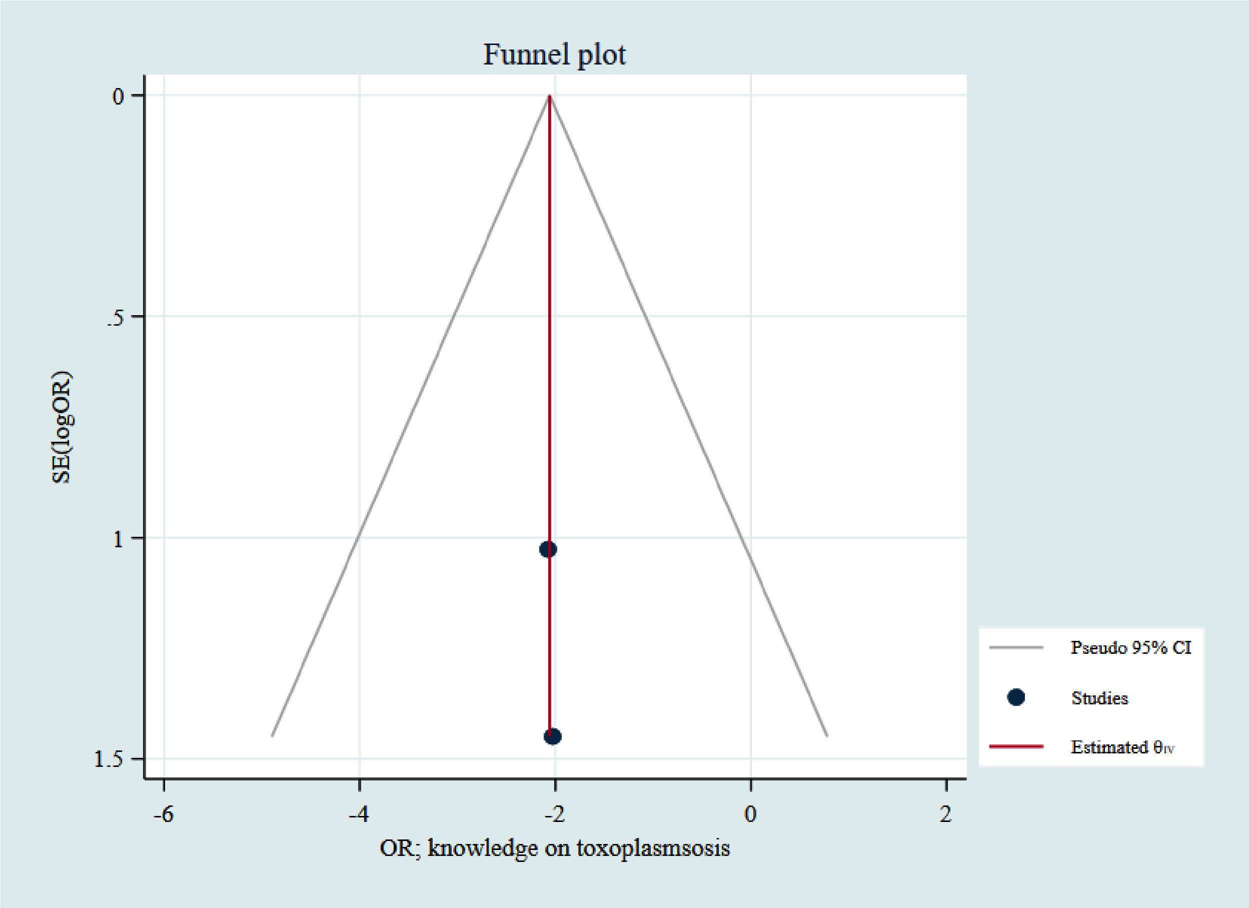

13. Number of pregnancy (more than one/first)

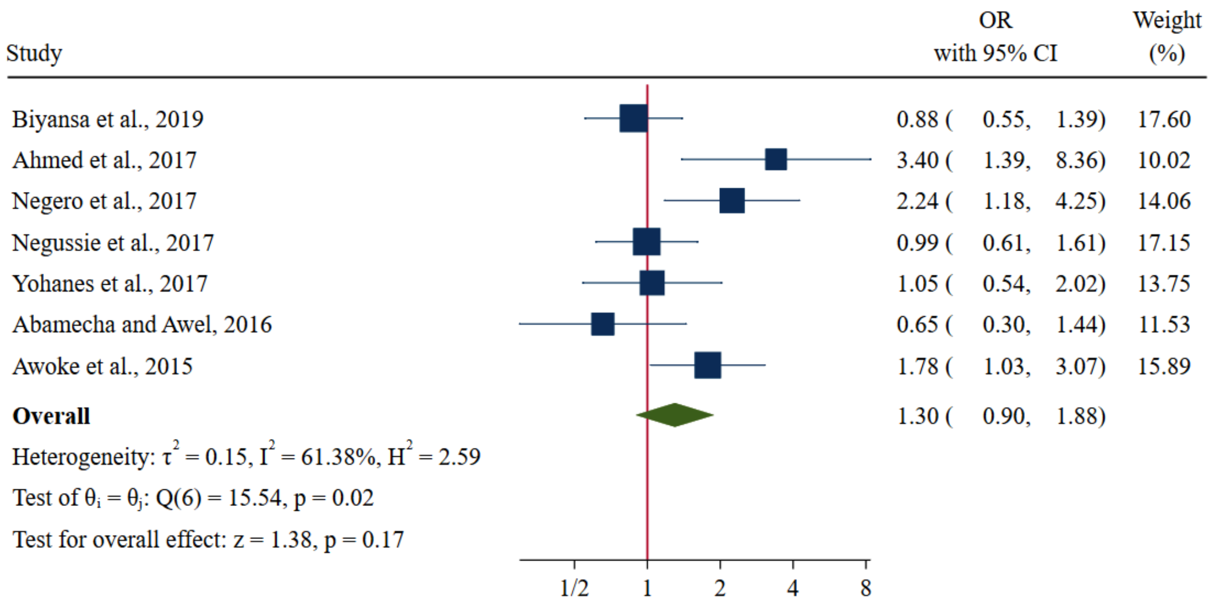

Random-effects DerSimonian-Laird model: number of pregnancy

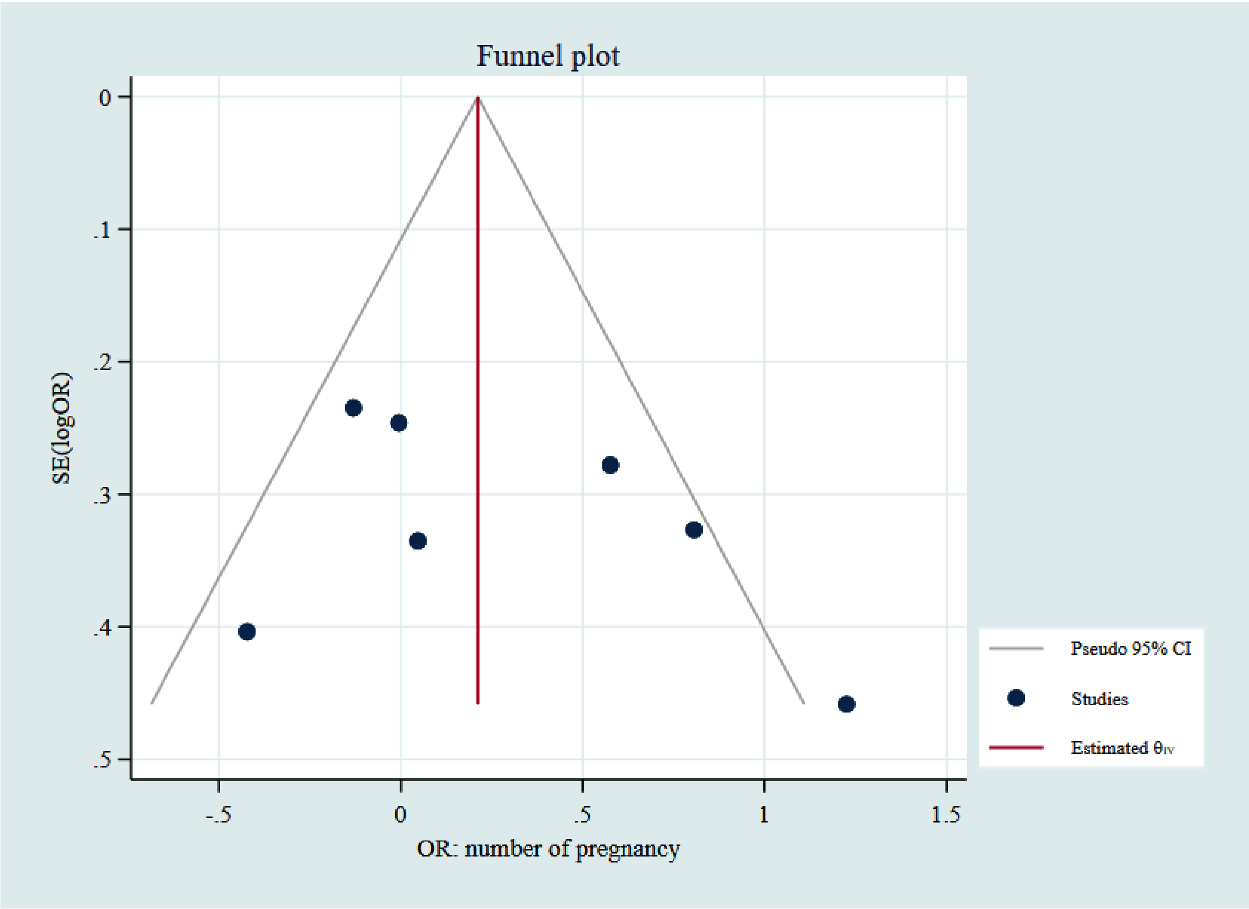

14. Occupation type (housewife & other activities)

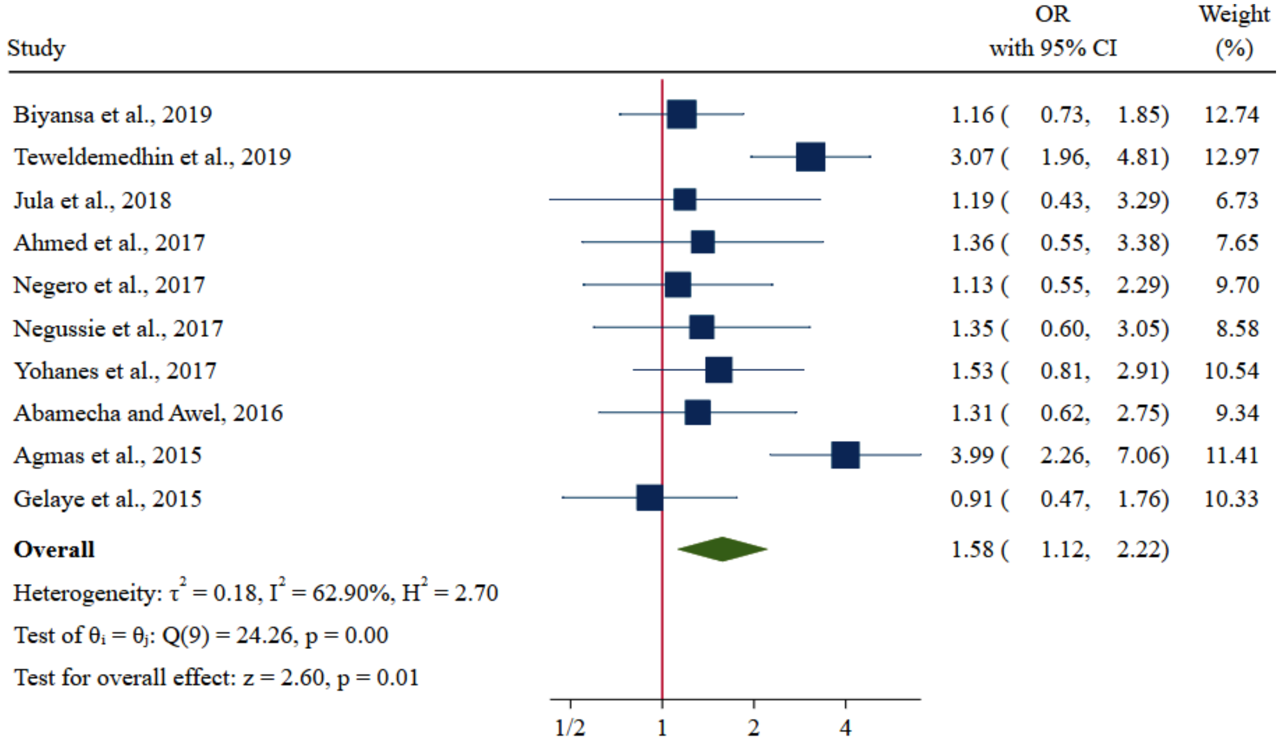

Random-effects DerSimonian-Laird model: being house wife vs other activities engaged

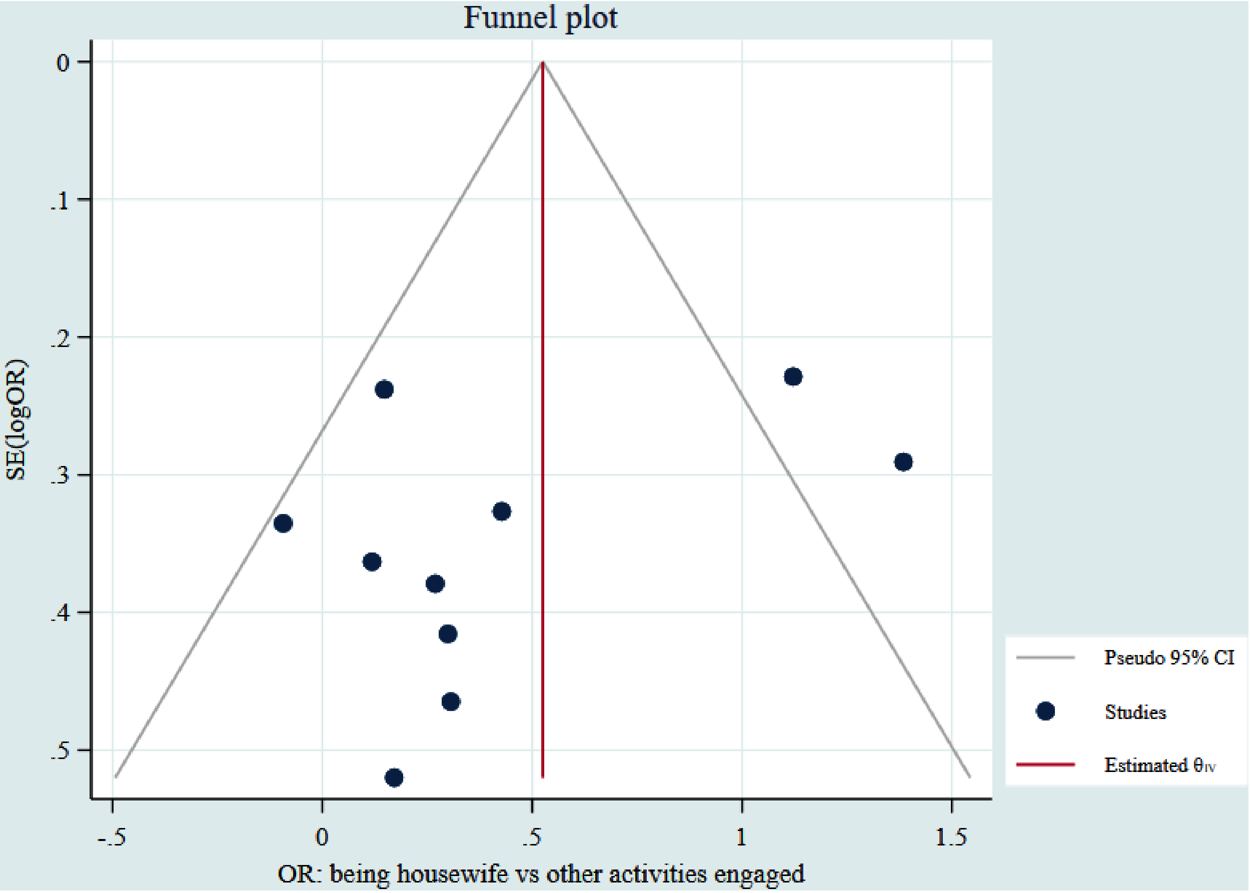

15. **Raw meat consumption habit (yes/no)**

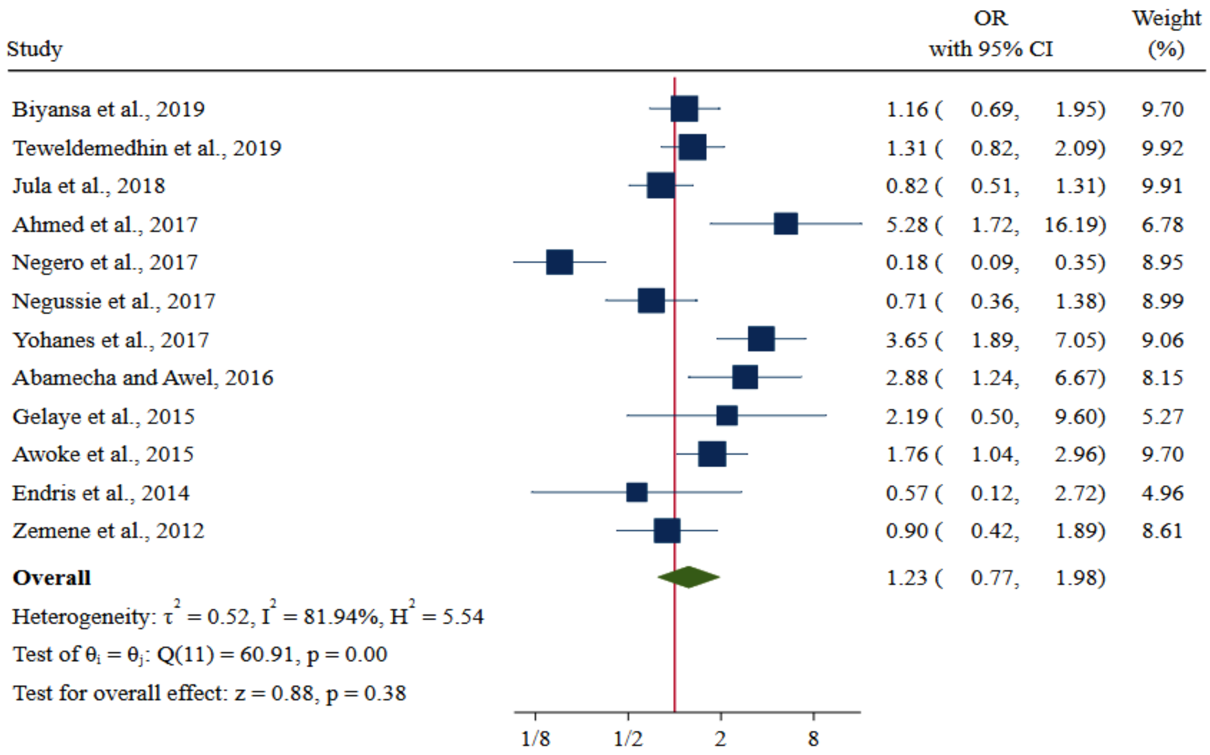

Random-effects DerSimonian-Laird model: raw meat consumption experince

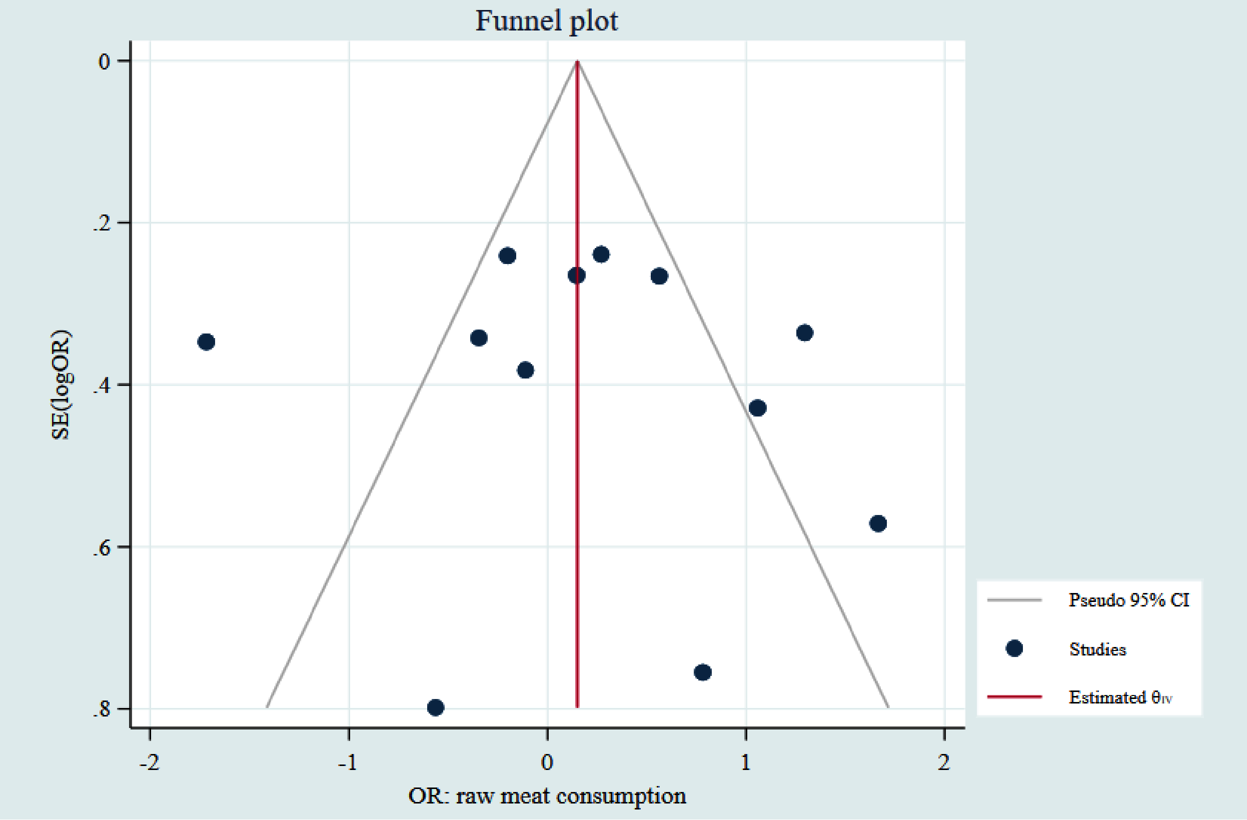

16. <u>Raw milk consumption habit(yes/no)</u>

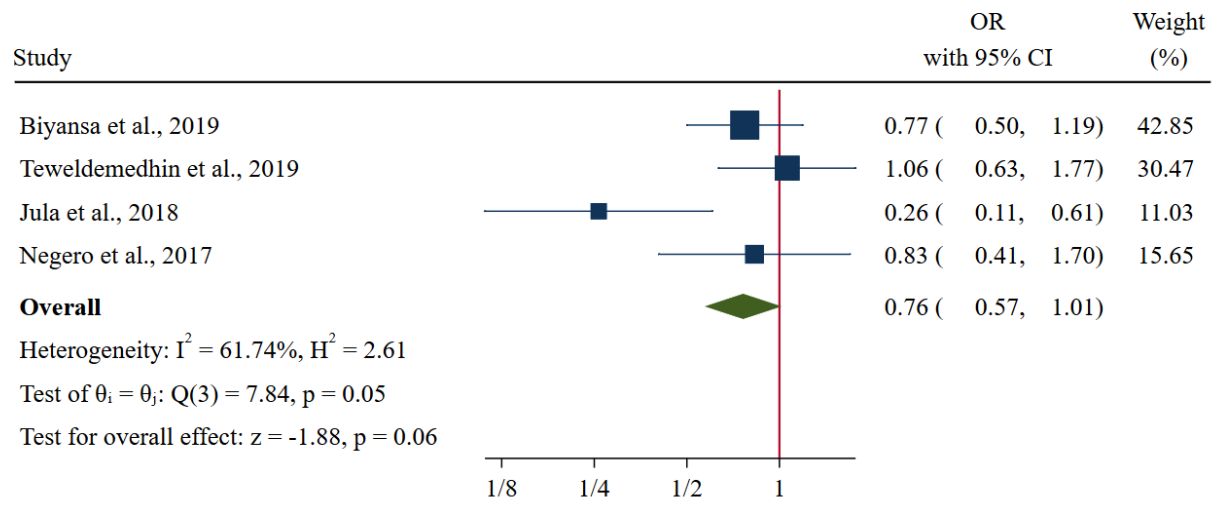

Fixed-effects inverse-variance model: raw milk consumption experience

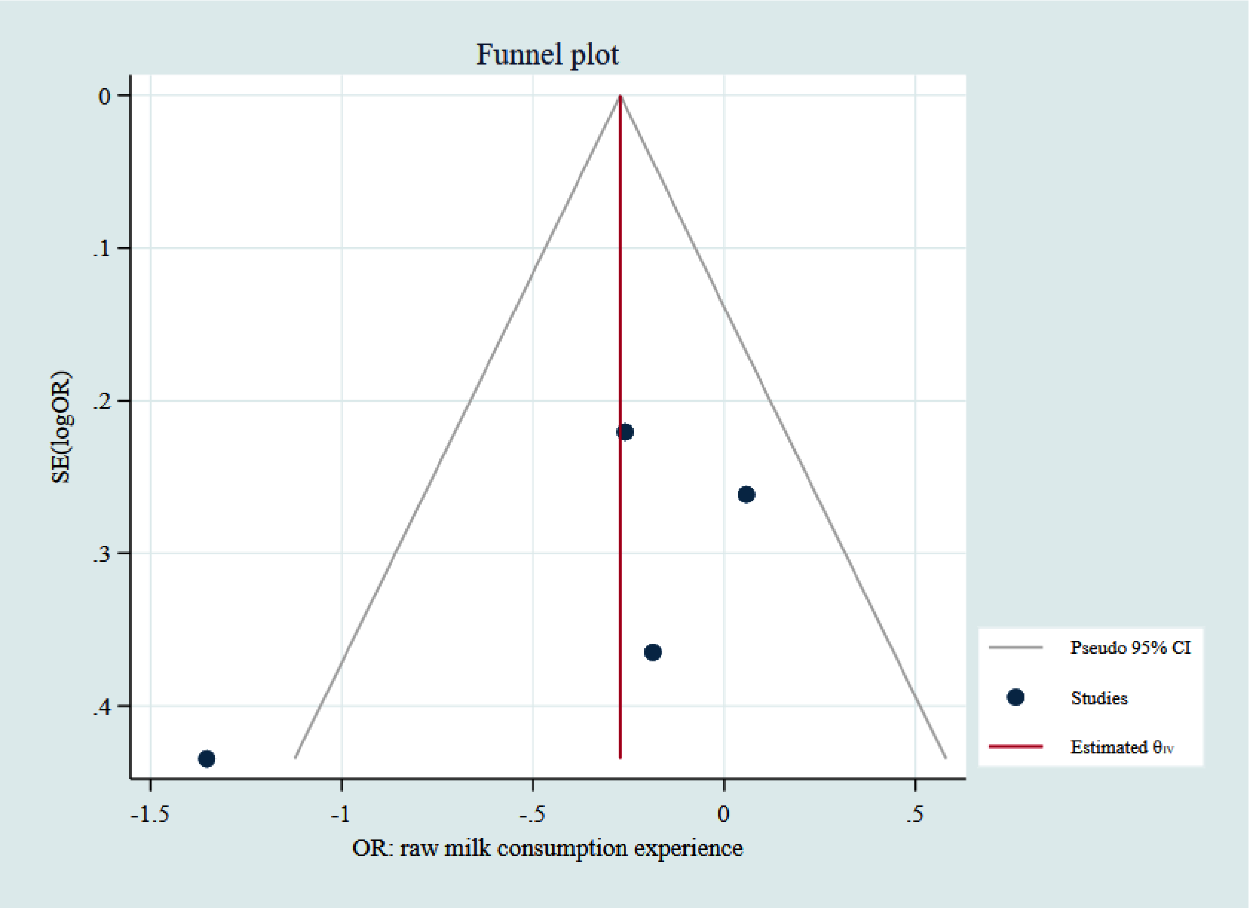

17. Raw vegetable consumption habit(yes/no)

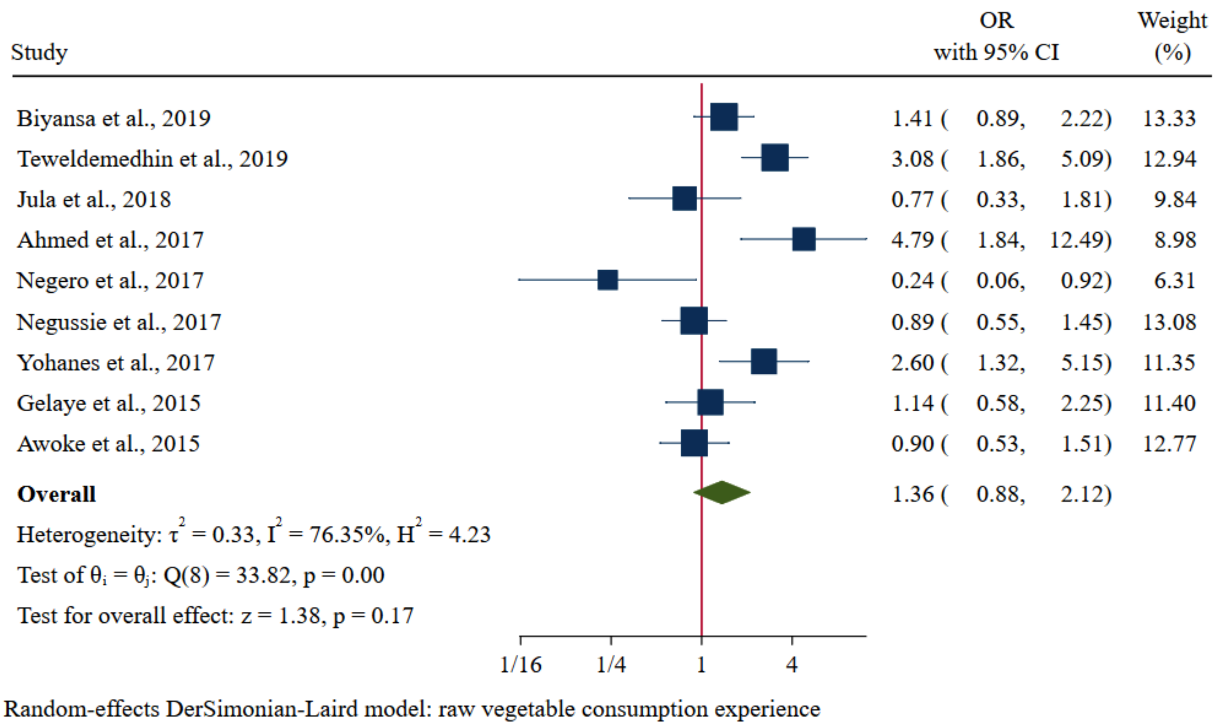

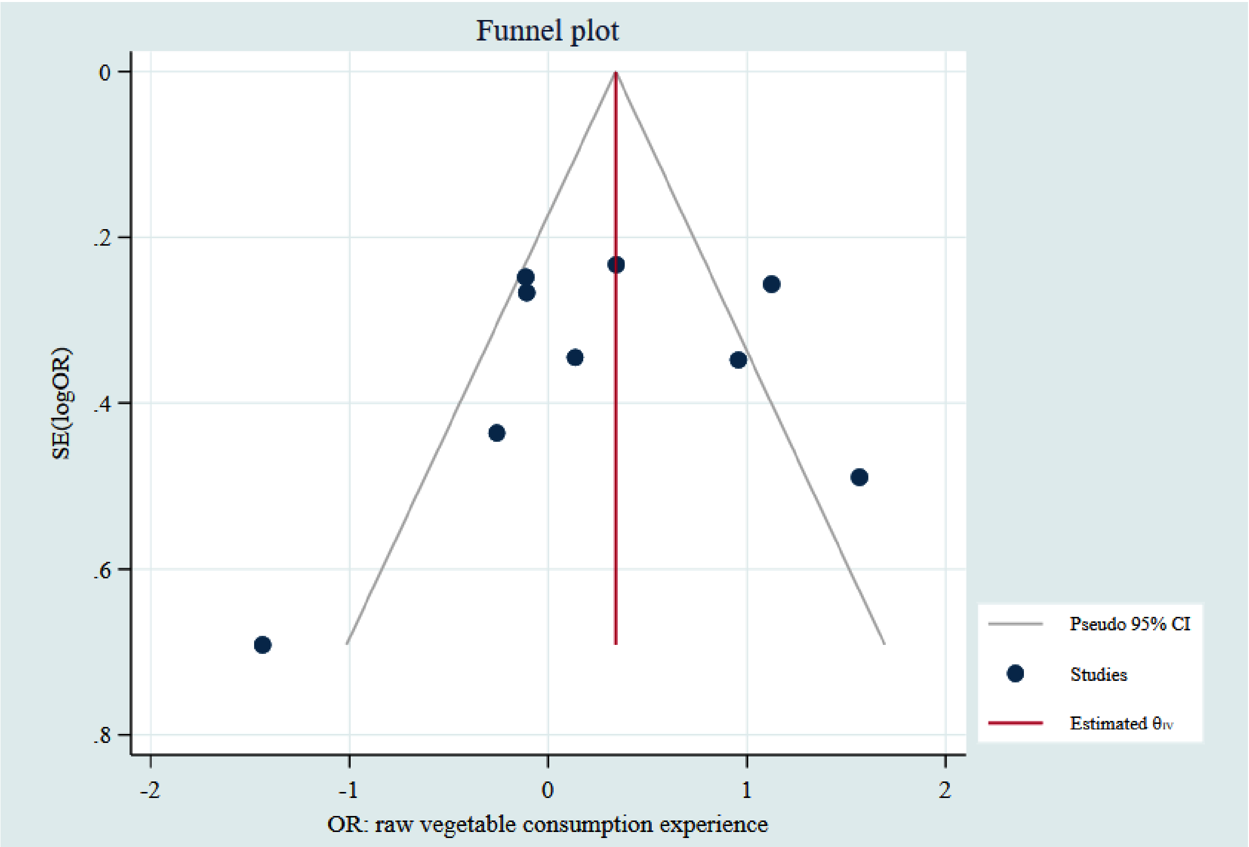

18. Religion (Christian vs Muslim)

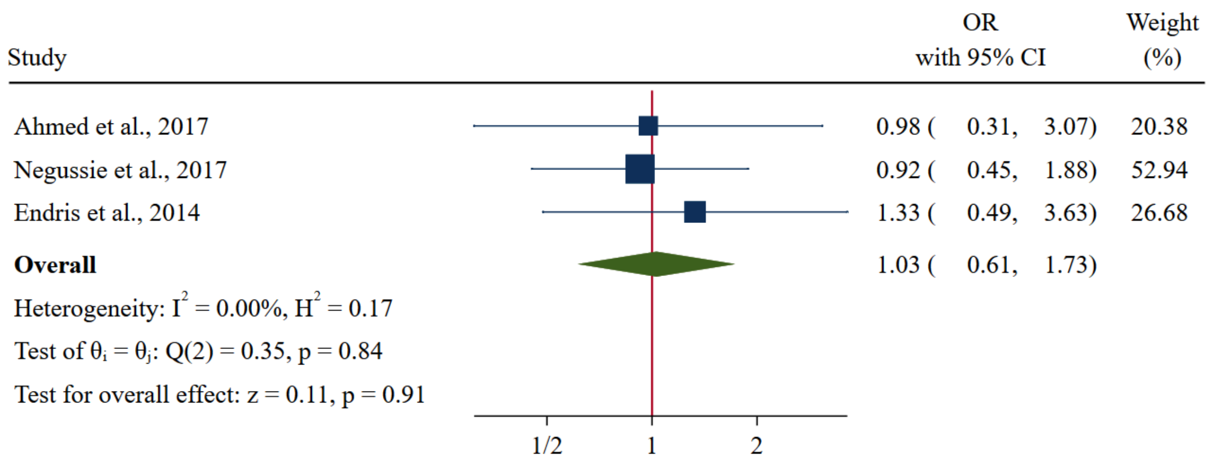

Fixed-effects inverse-variance model: religion

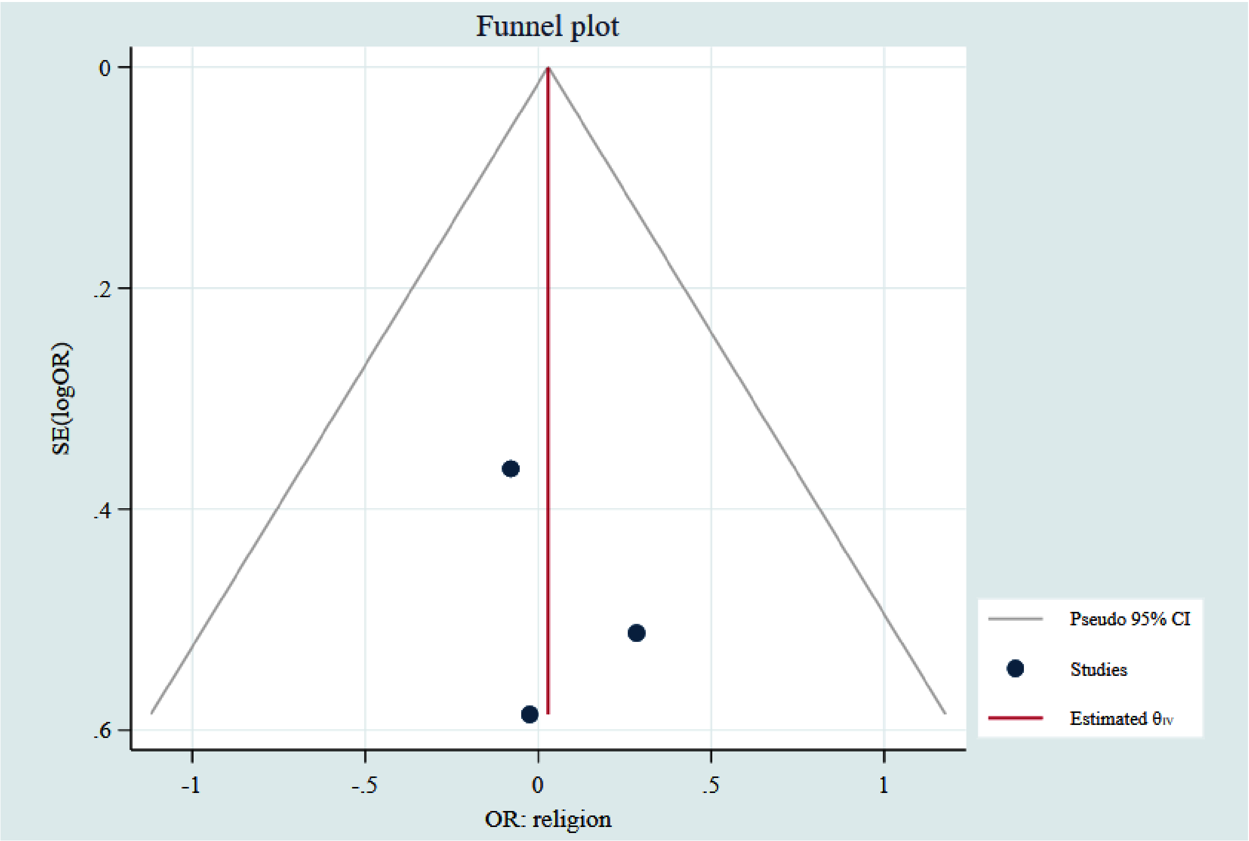

19. Residence (rural vs urban)

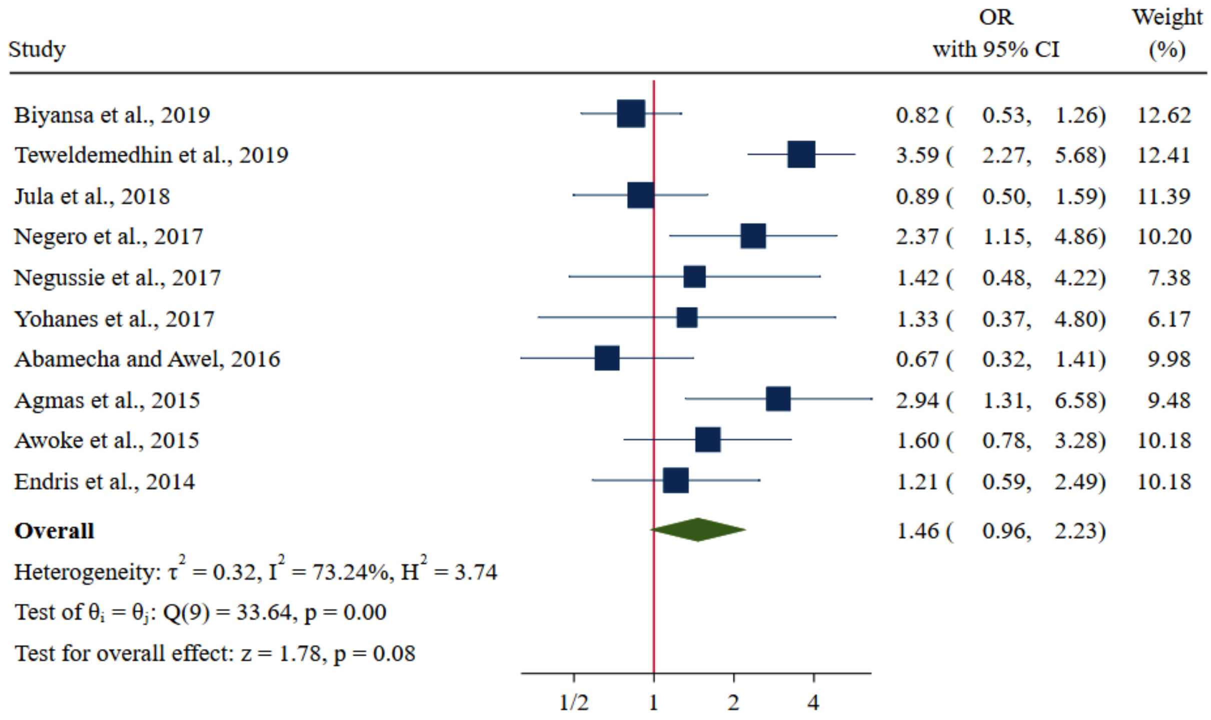

Random-effects DerSimonian-Laird model: residence

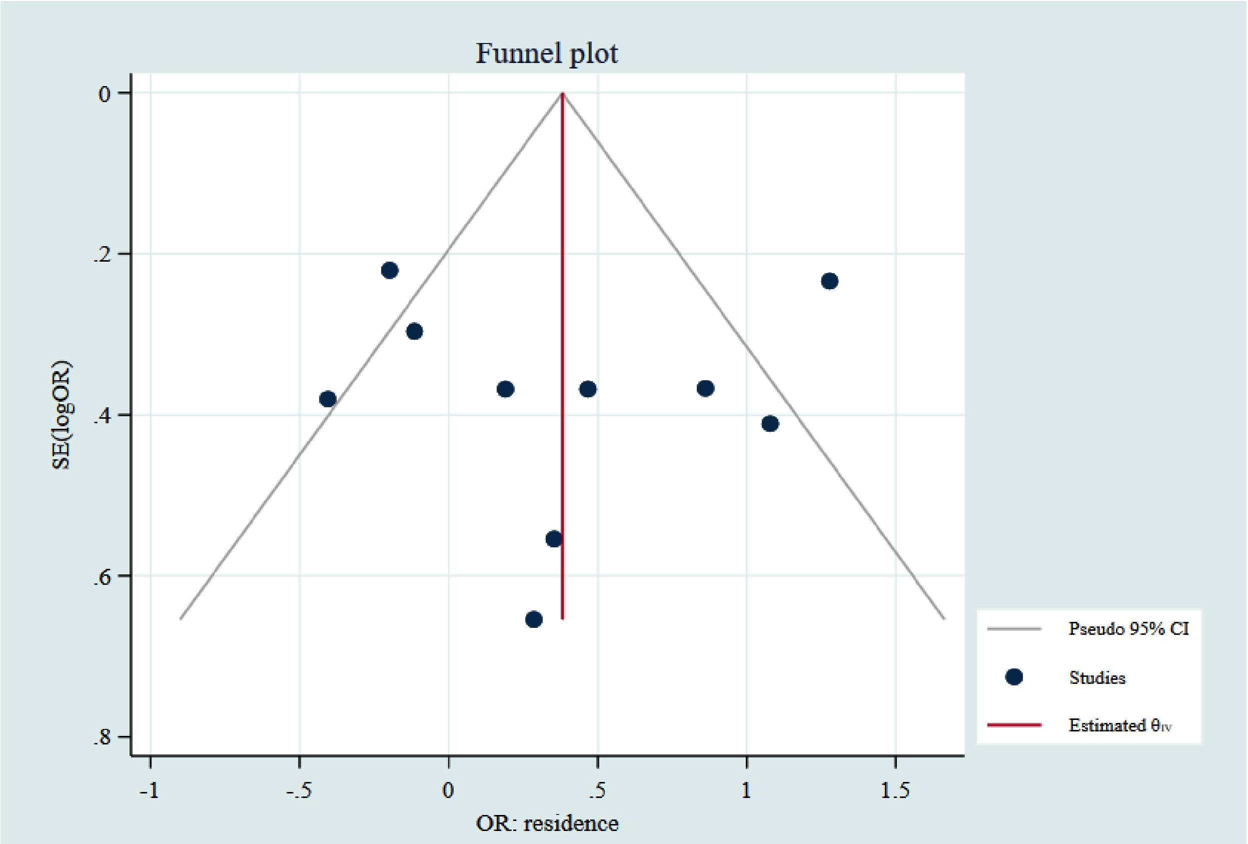

20. <u>Water source quality (unsafe/safe)</u>

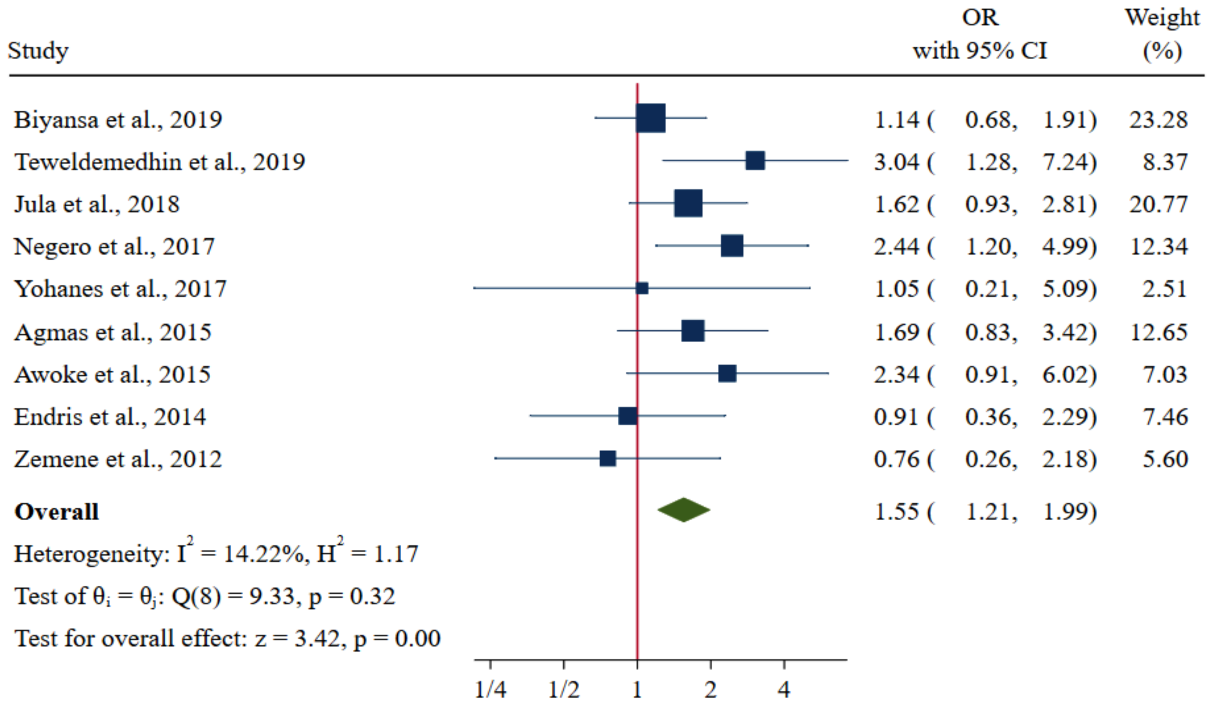

Fixed-effects inverse-variance model: water source

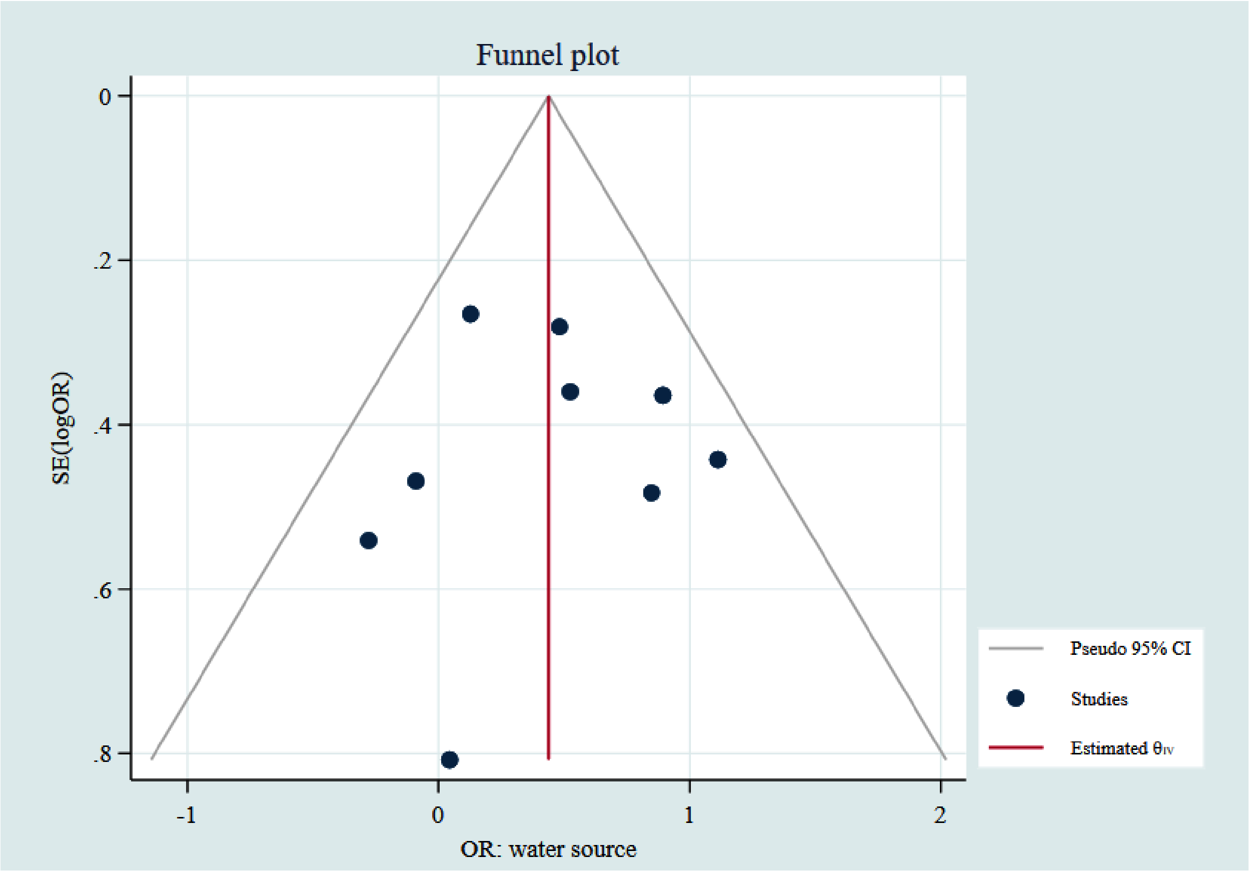

Supplement: S1 Fig — (PDF) [file pntd.0008944.s004.pdf]
