# Supplementary material for: Potential risk factors associated with seropositivity for Toxoplasma gondii among pregnant women and HIV infected individuals in Ethiopia: A systematic review and meta-analysis
Source: PLoS Negl Trop Dis. 2020 Dec 15;14(12):e0008944. doi: 10.1371/journal.pntd.0008944 (PMC7771857; doi:10.1371/journal.pntd.0008944)

Potential risk factors for *T. gondii* seropositivity in HIV infected individuals of Ethiopia

1. Age category (≥25 vs < 25 years)

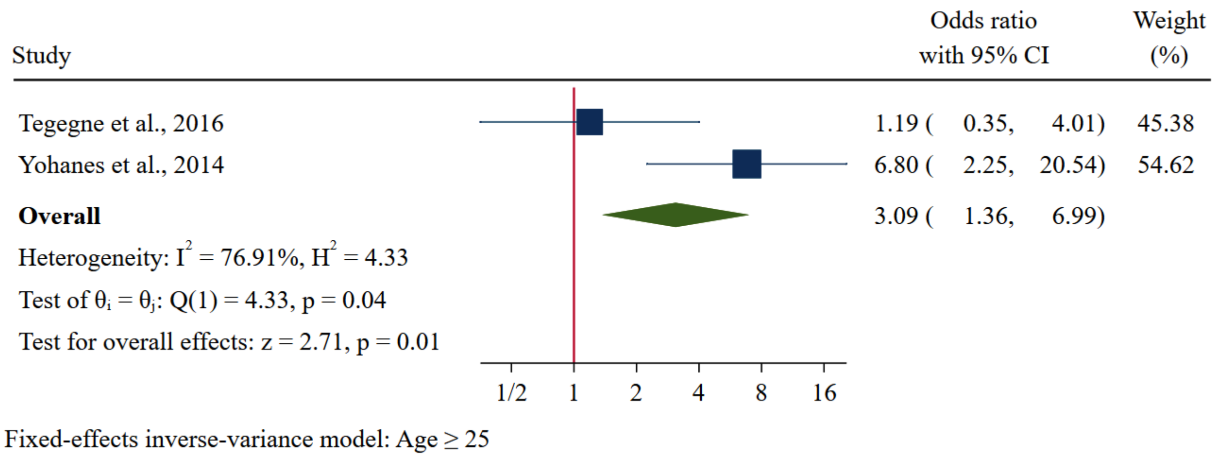

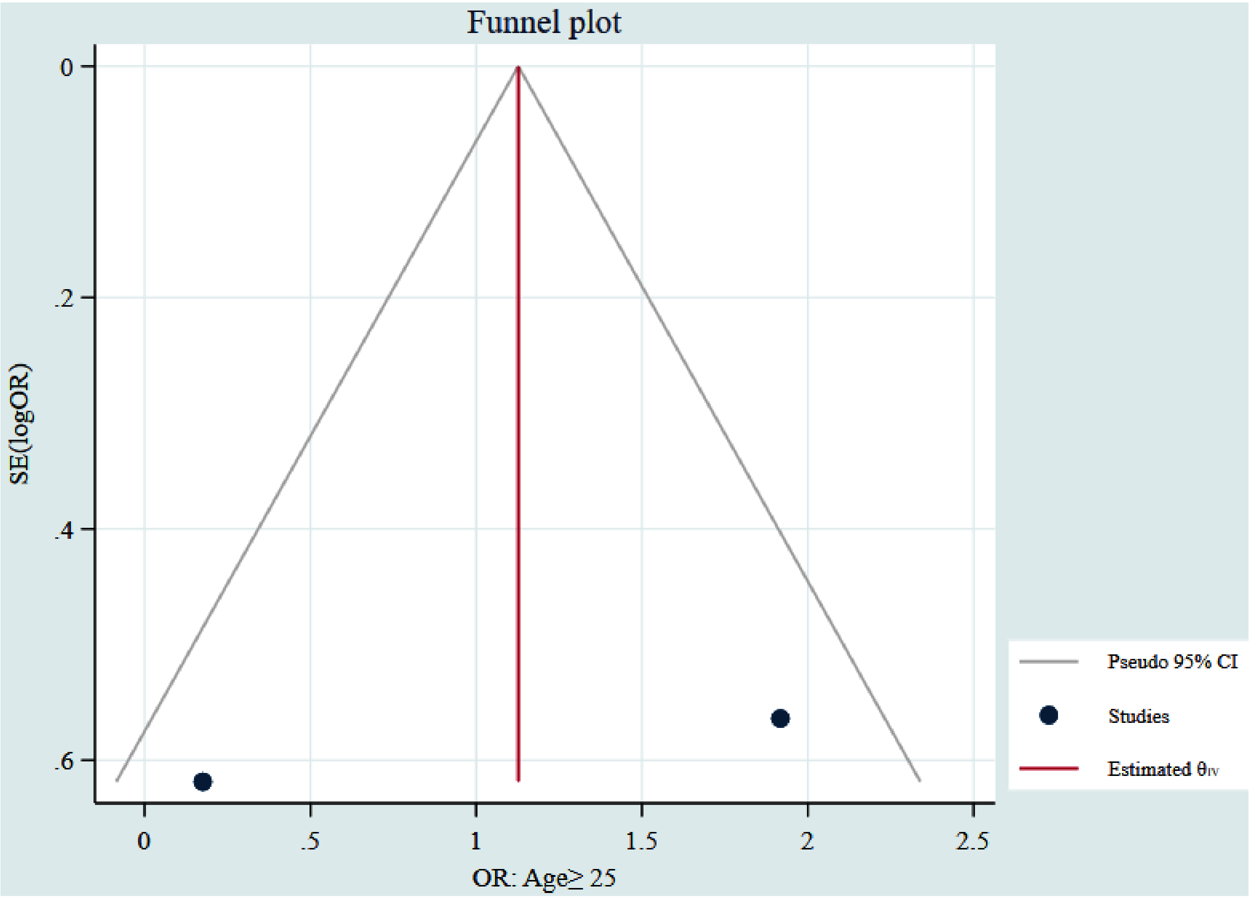

2. <u>Blood transfusion experience (yes/no)</u>

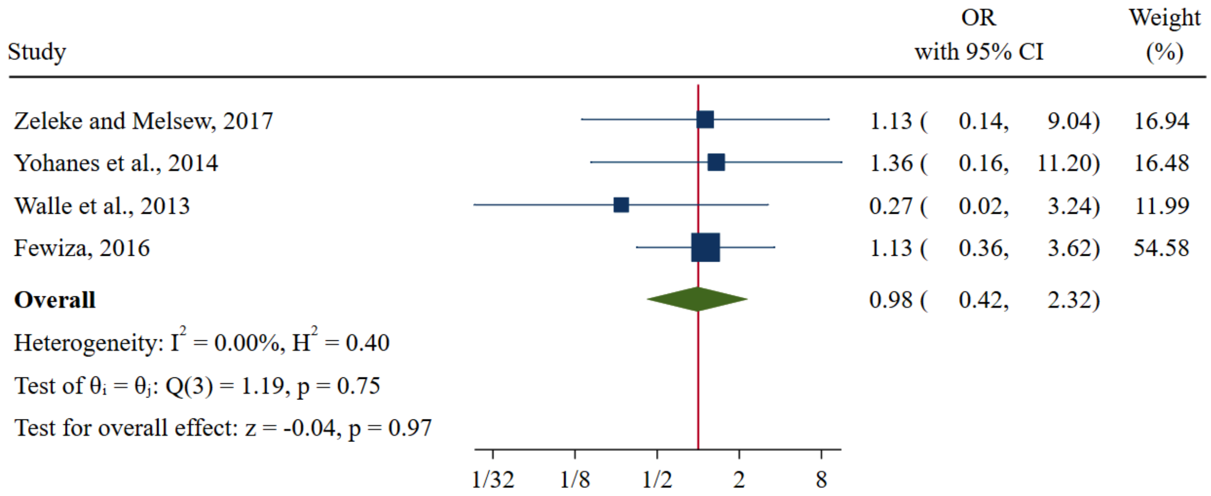

Fixed-effects inverse-variance model: blood transfusion

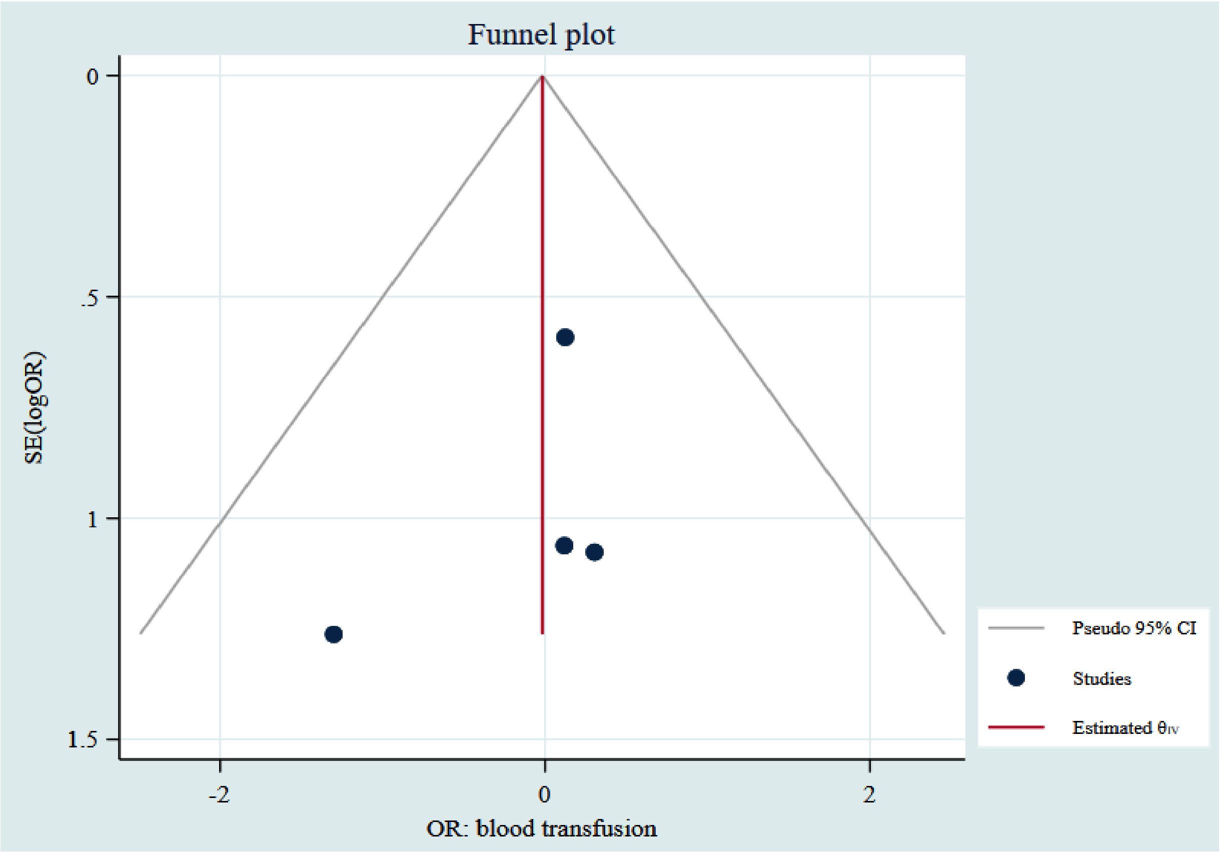

3. Cat presence/ownership (yes/no)

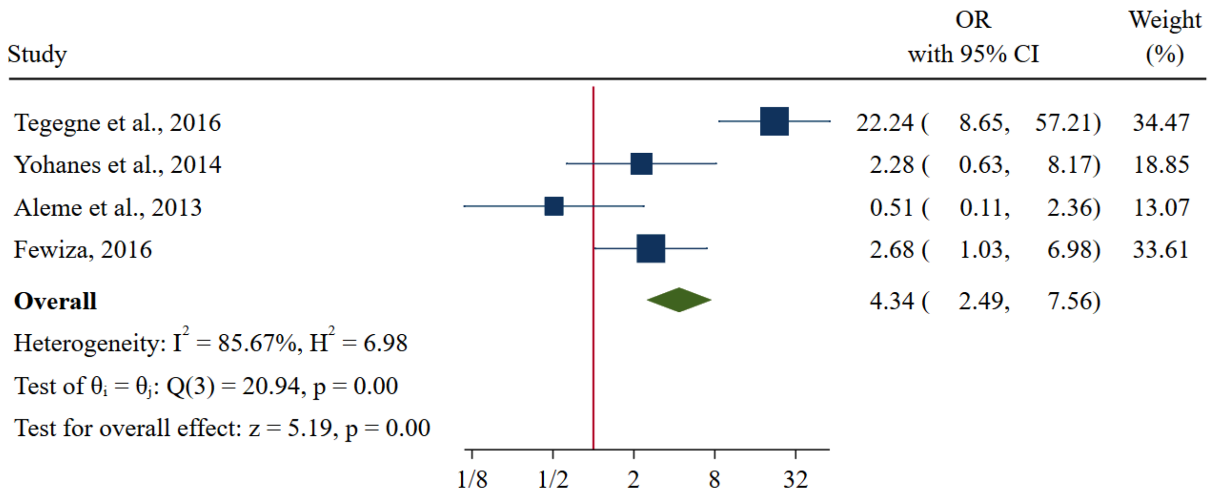

Fixed-effects inverse-variance model: cat presence/ownership

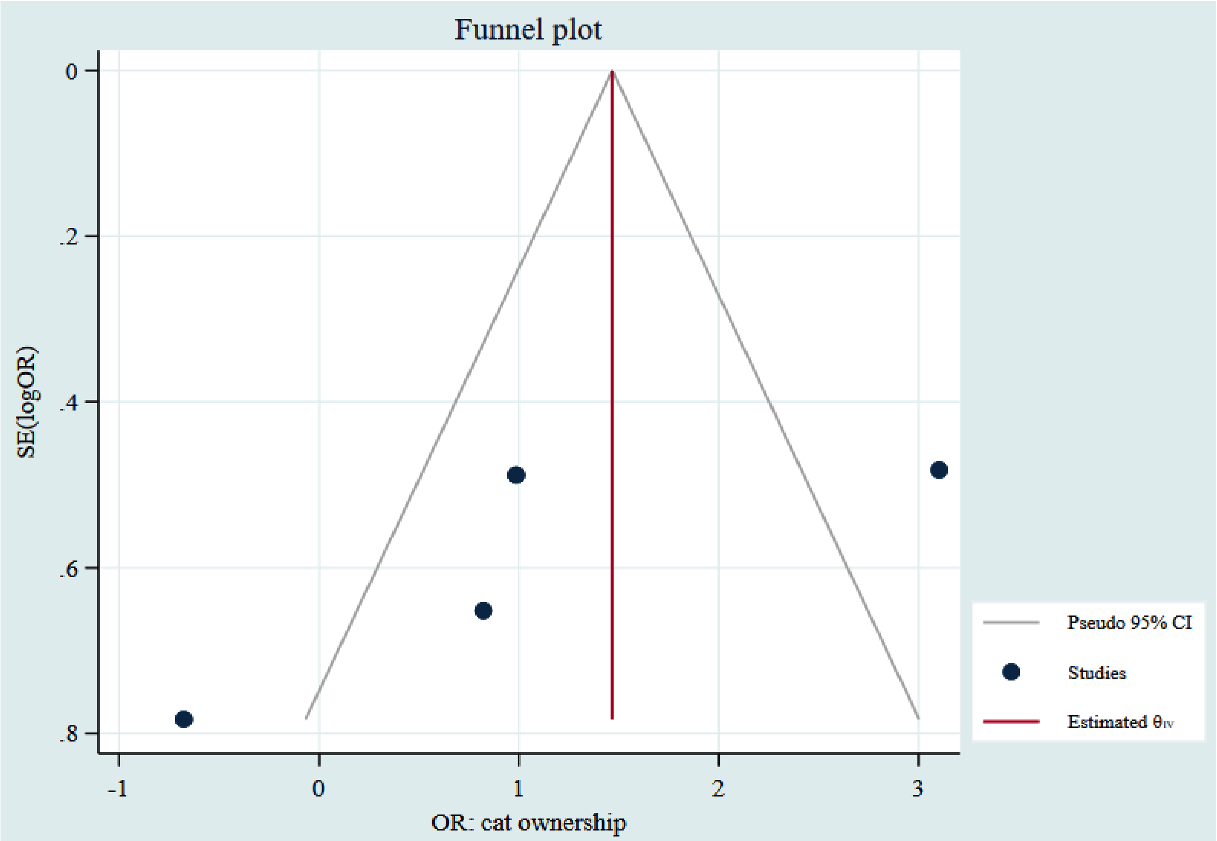

4. Contact with cat(yes/no)

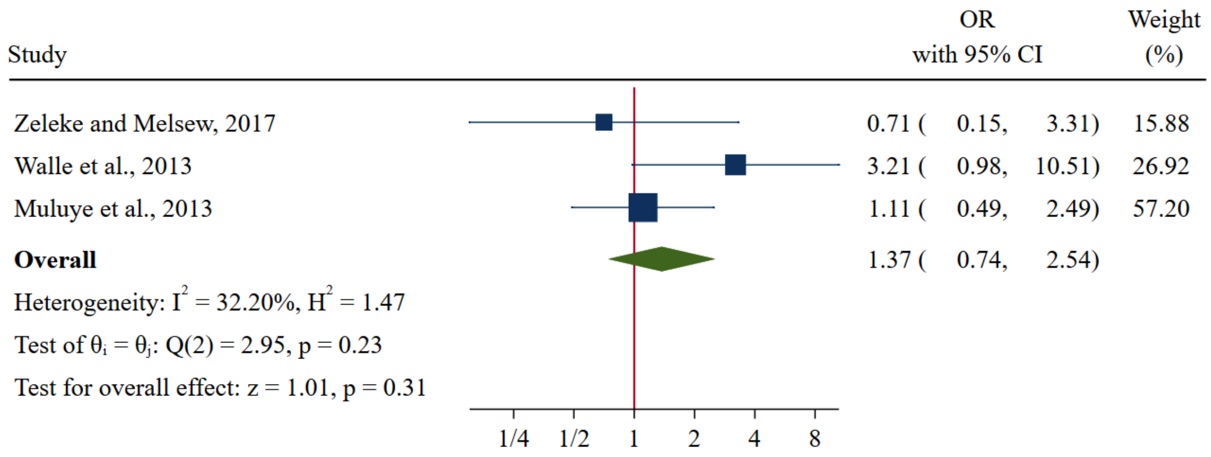

Fixed-effects inverse-variance model: contact with cat

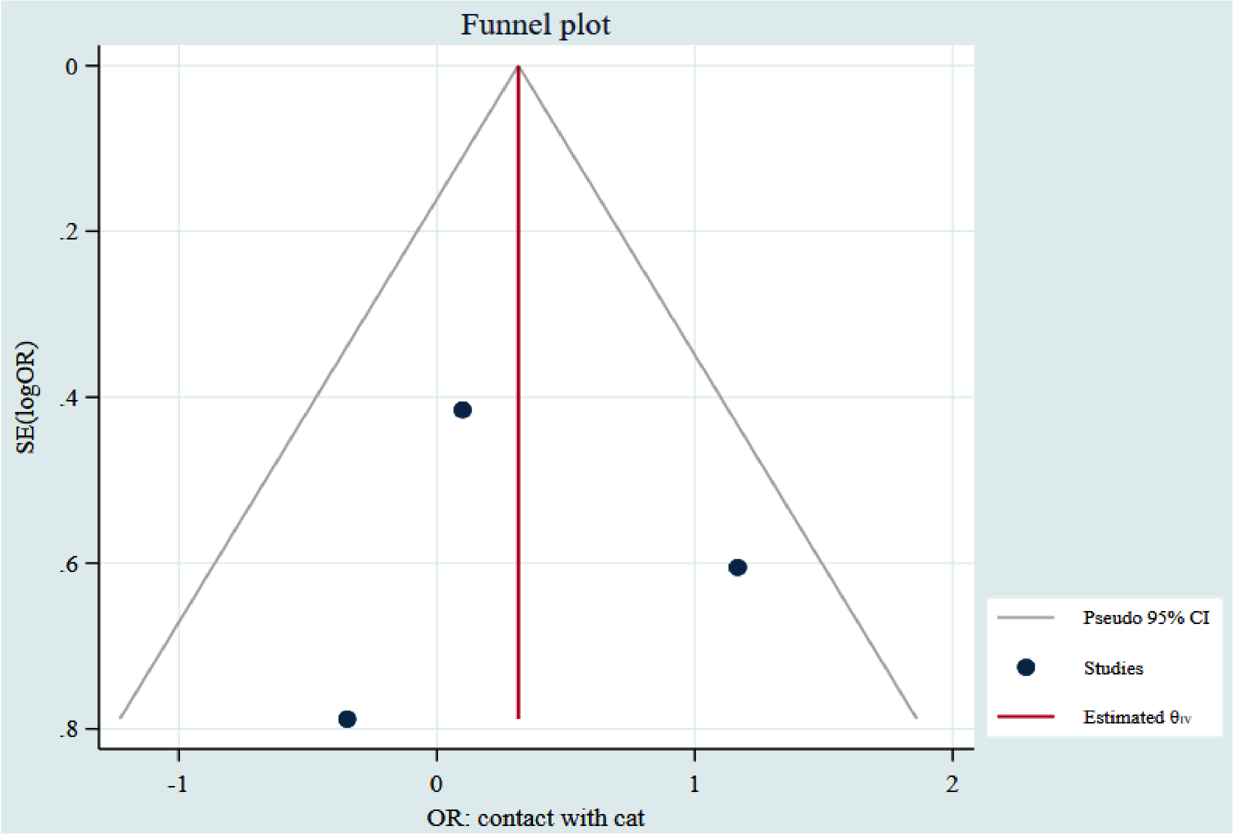

5. <u>Educational level (illiterate/literate)</u>

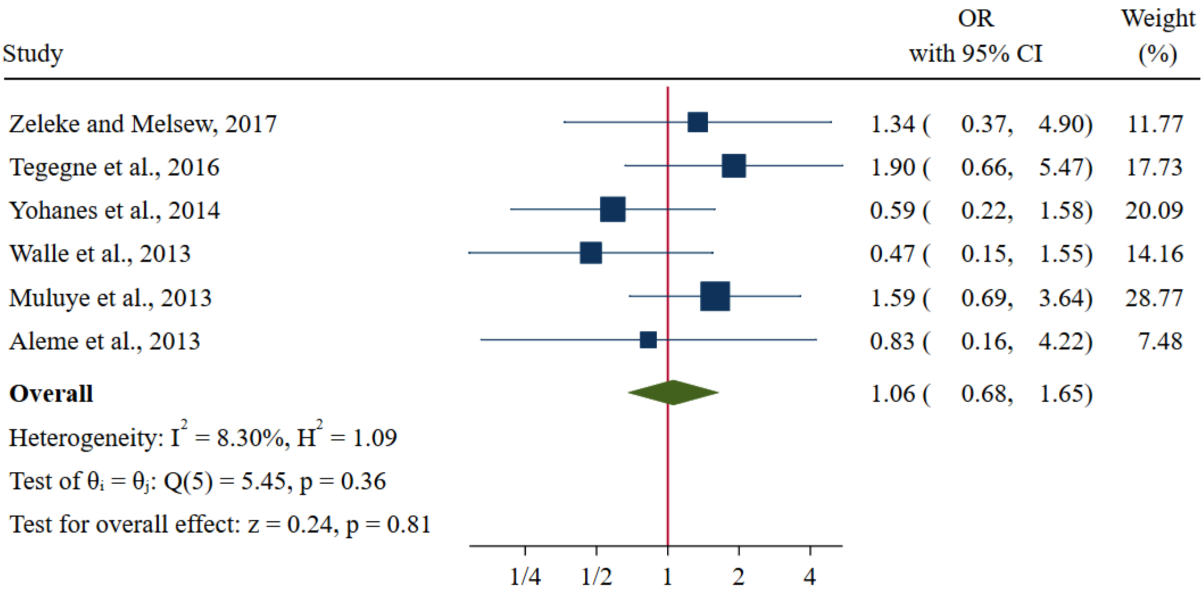

Fixed-effects inverse-variance model: educational level

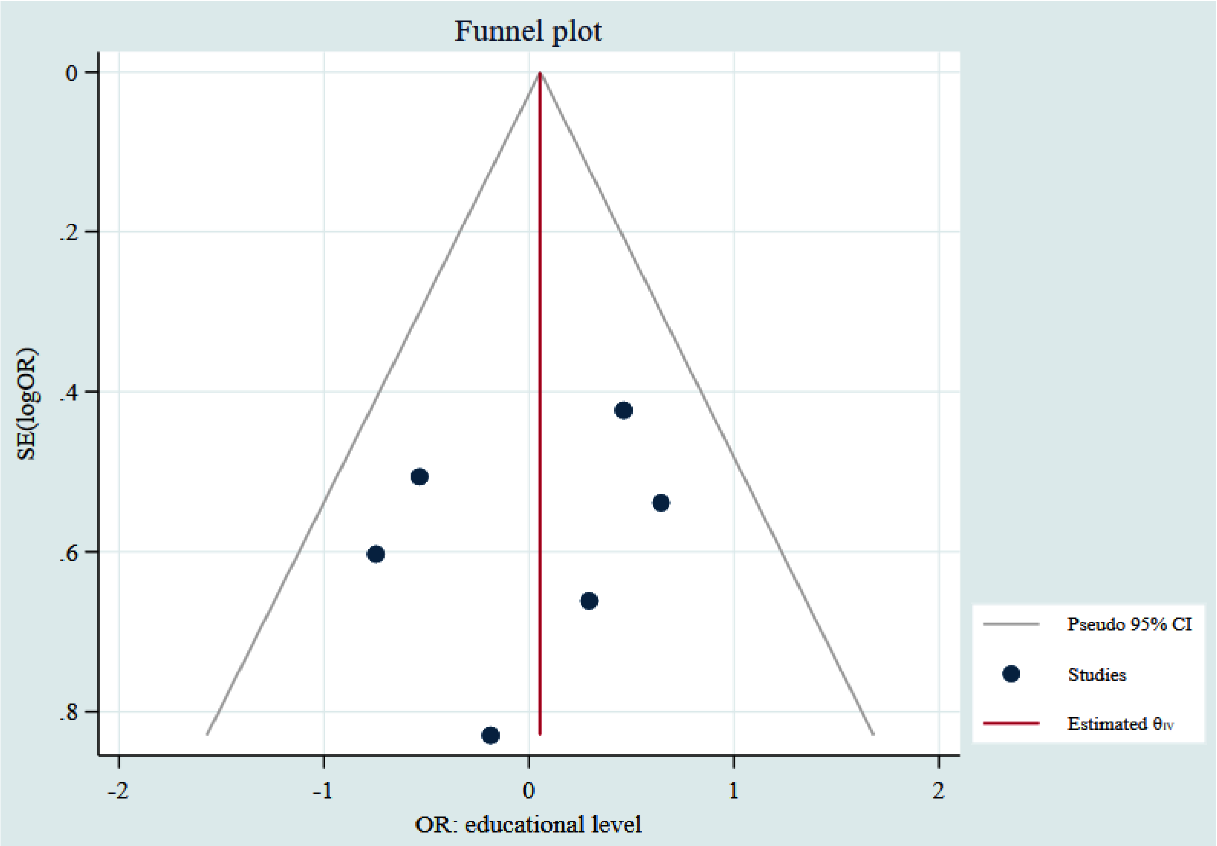

6. Hand washing habit after meat contact (sometimes/regularly)

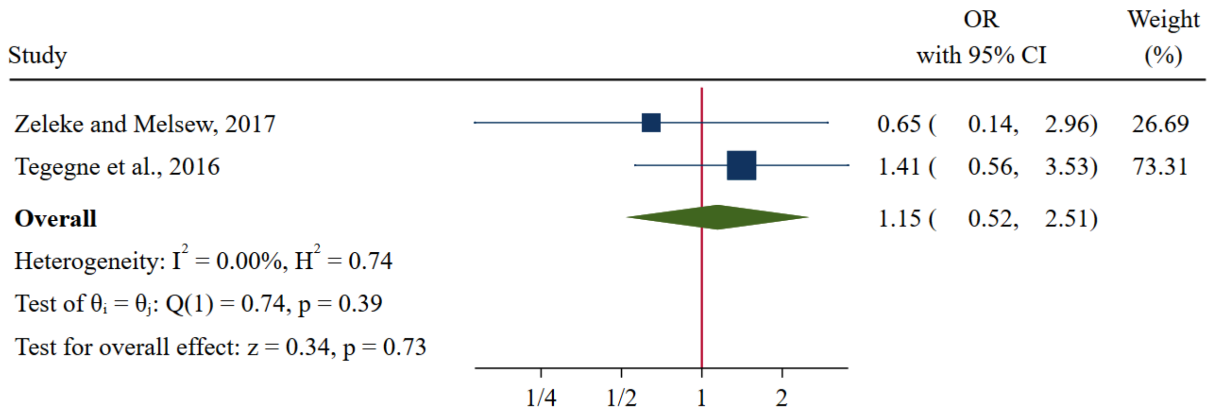

Fixed-effects inverse-variance model: handwashing habit

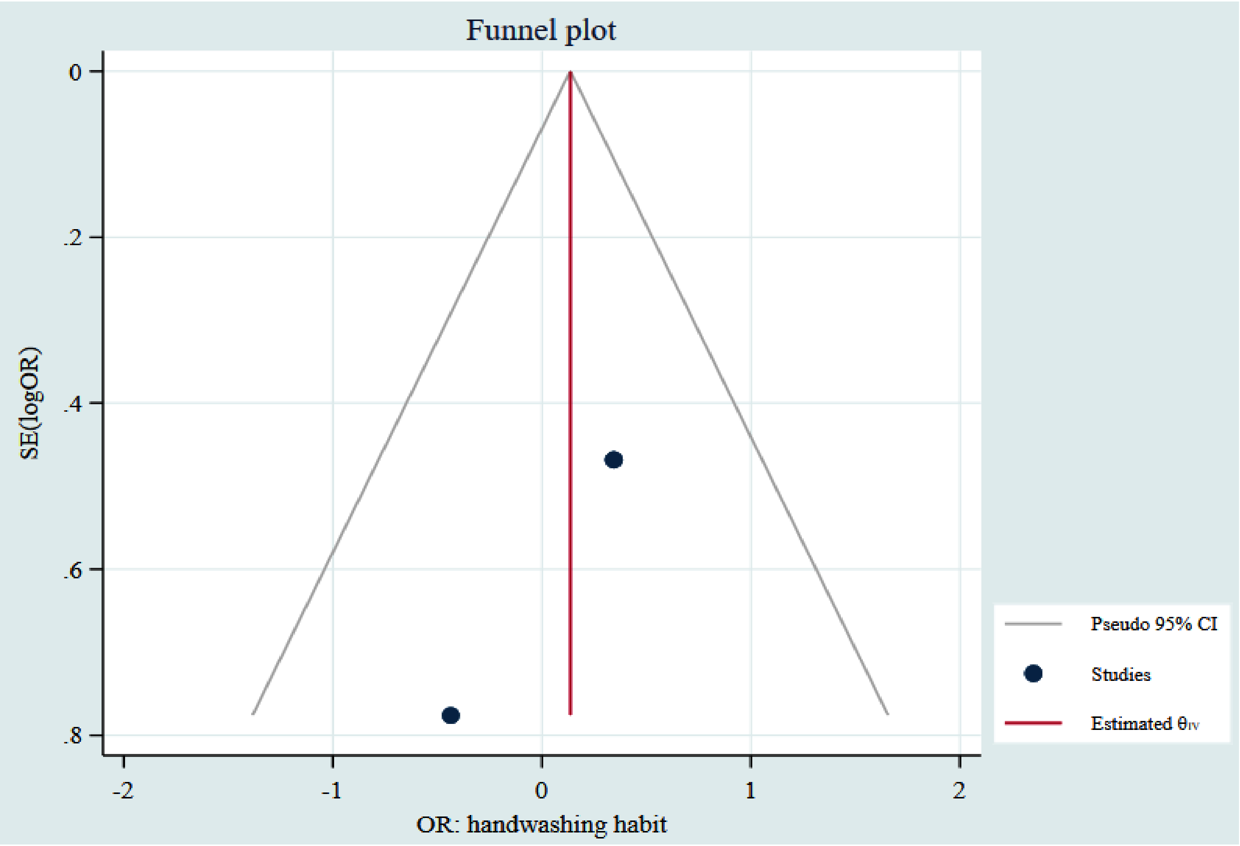

7. Knowledge on toxoplasmosis (yes/no)

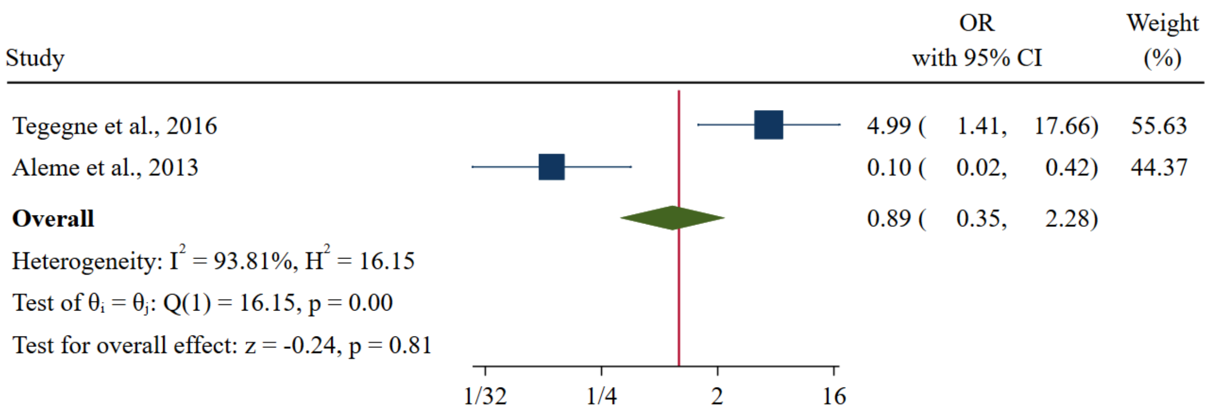

Fixed-effects inverse-variance model: knowledge on toxoplasmosis

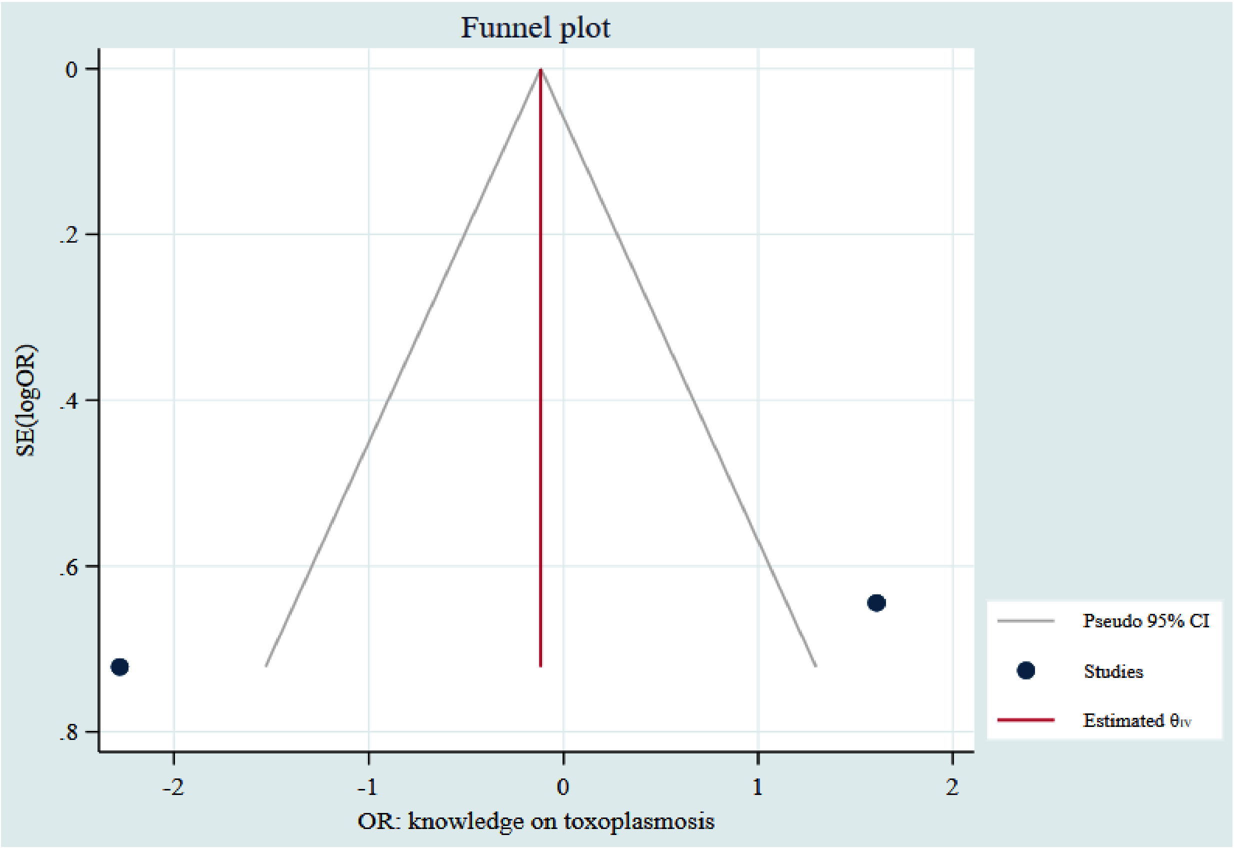

8. Marital status (single/couple)

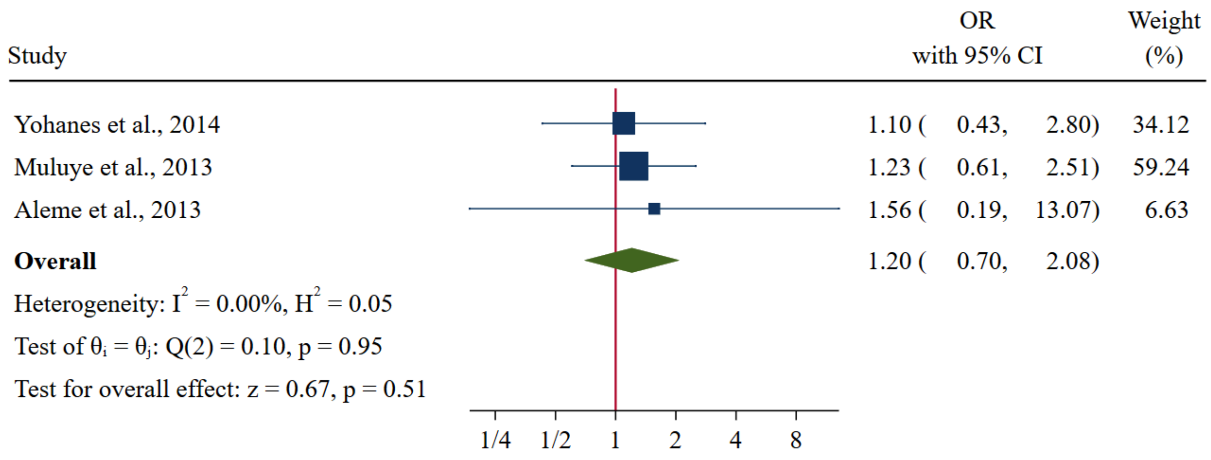

Fixed-effects inverse-variance model: marital status

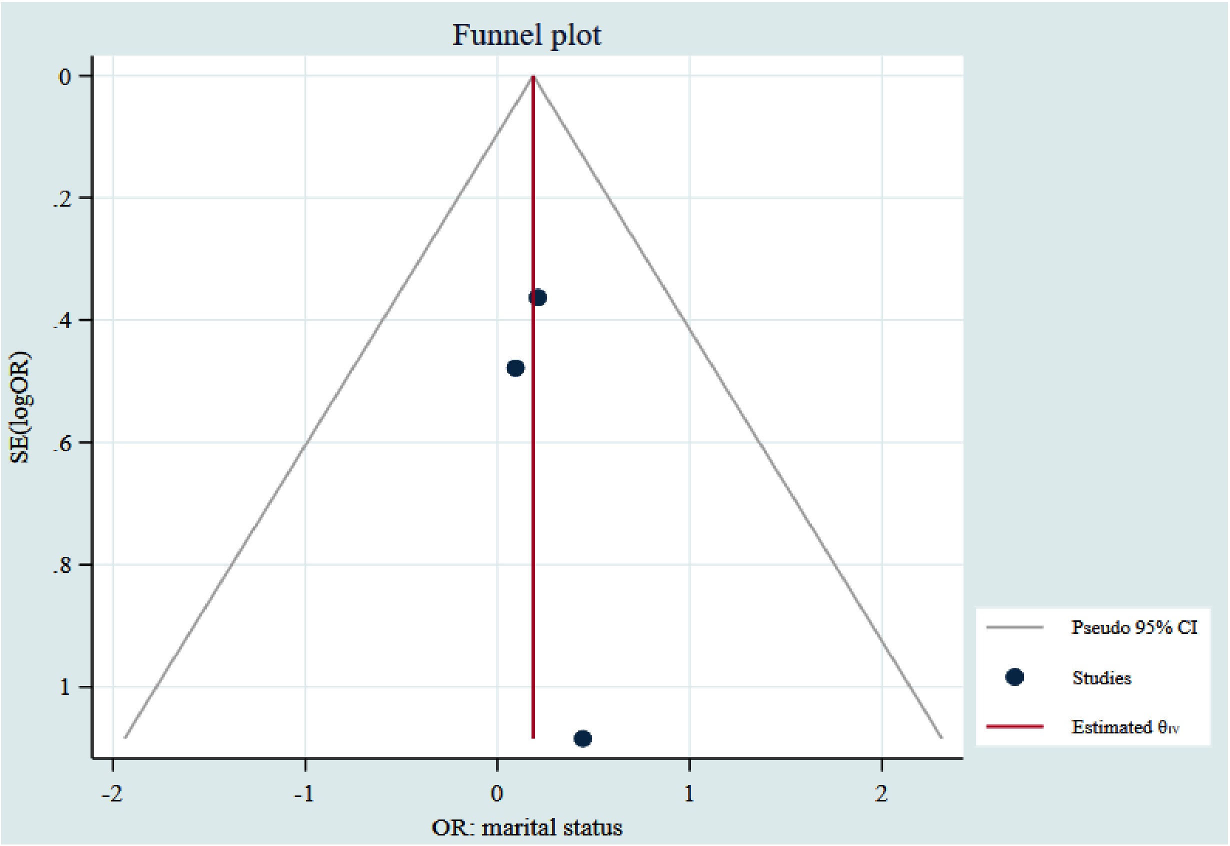

9. Raw meat consumption habit(yes/no)

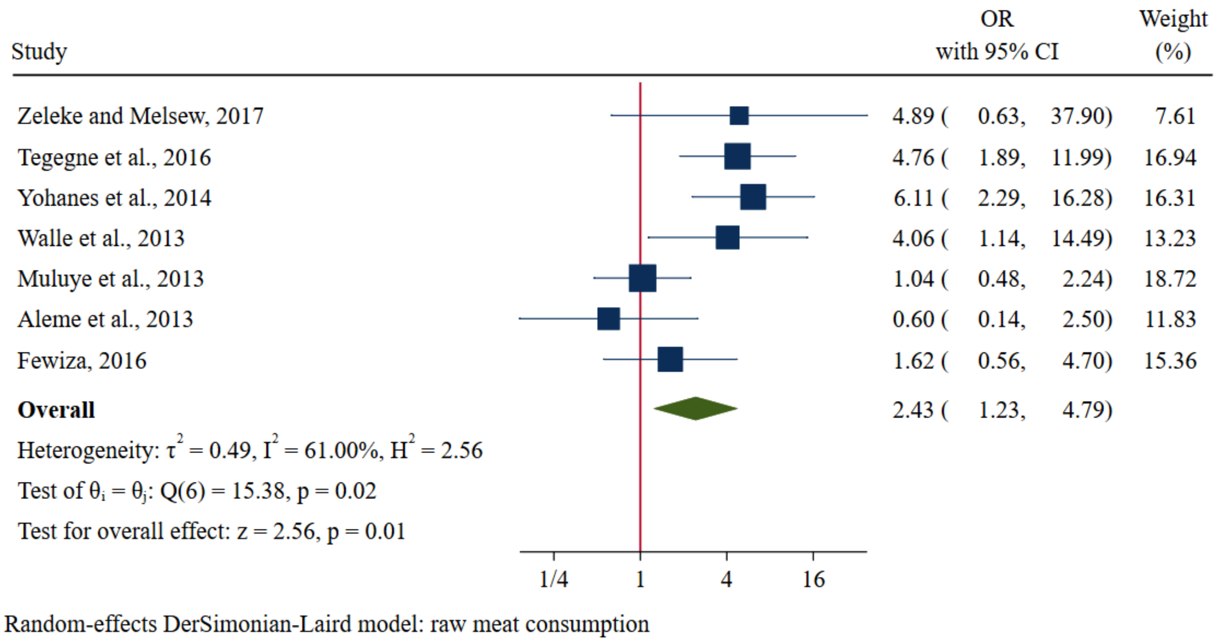

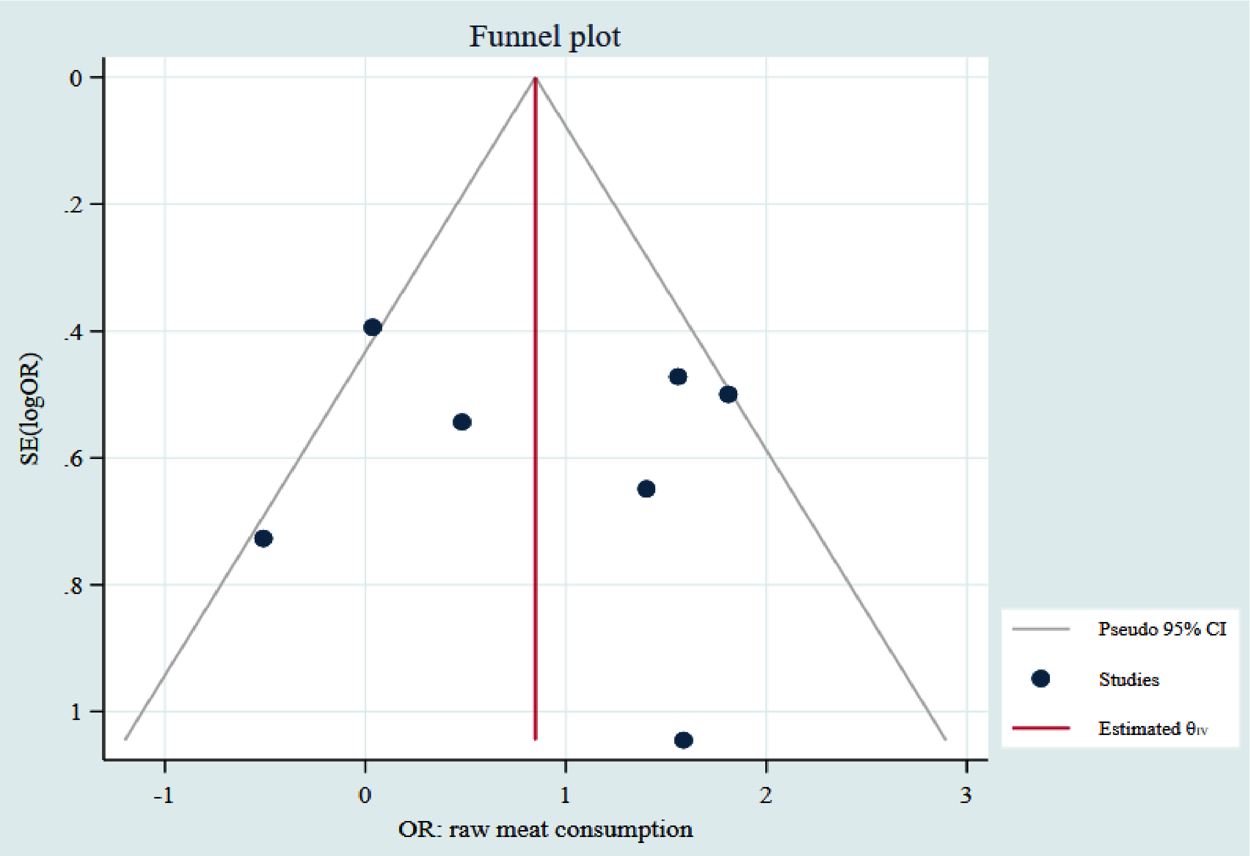

10. <u>Raw vegetable consumption habit (yes/no)</u>

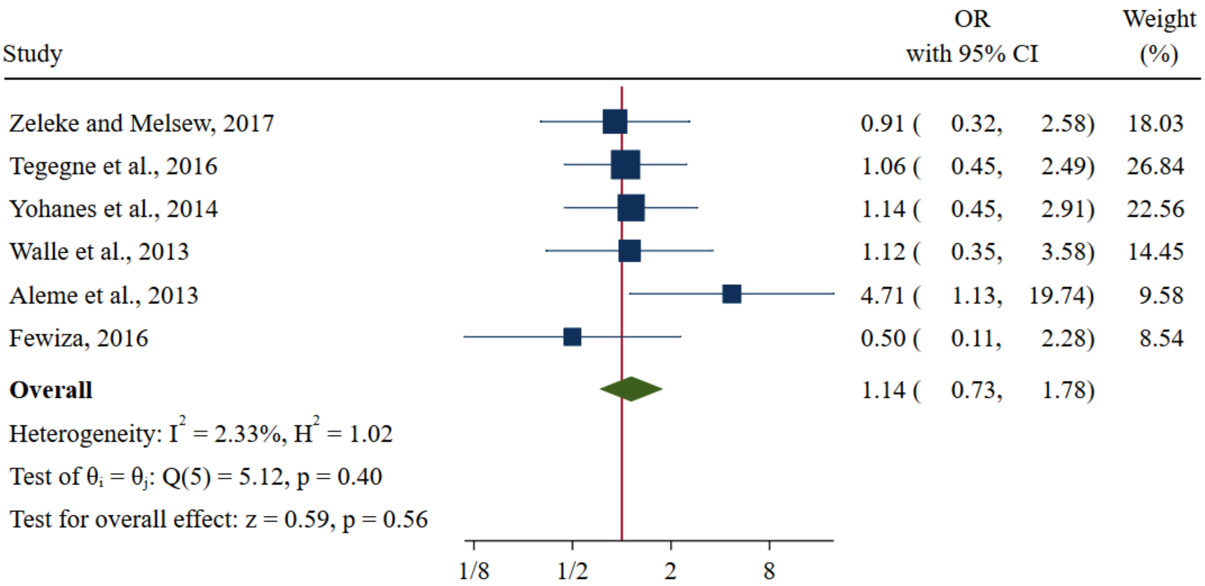

Fixed-effects inverse-variance model: raw vegetable consumption

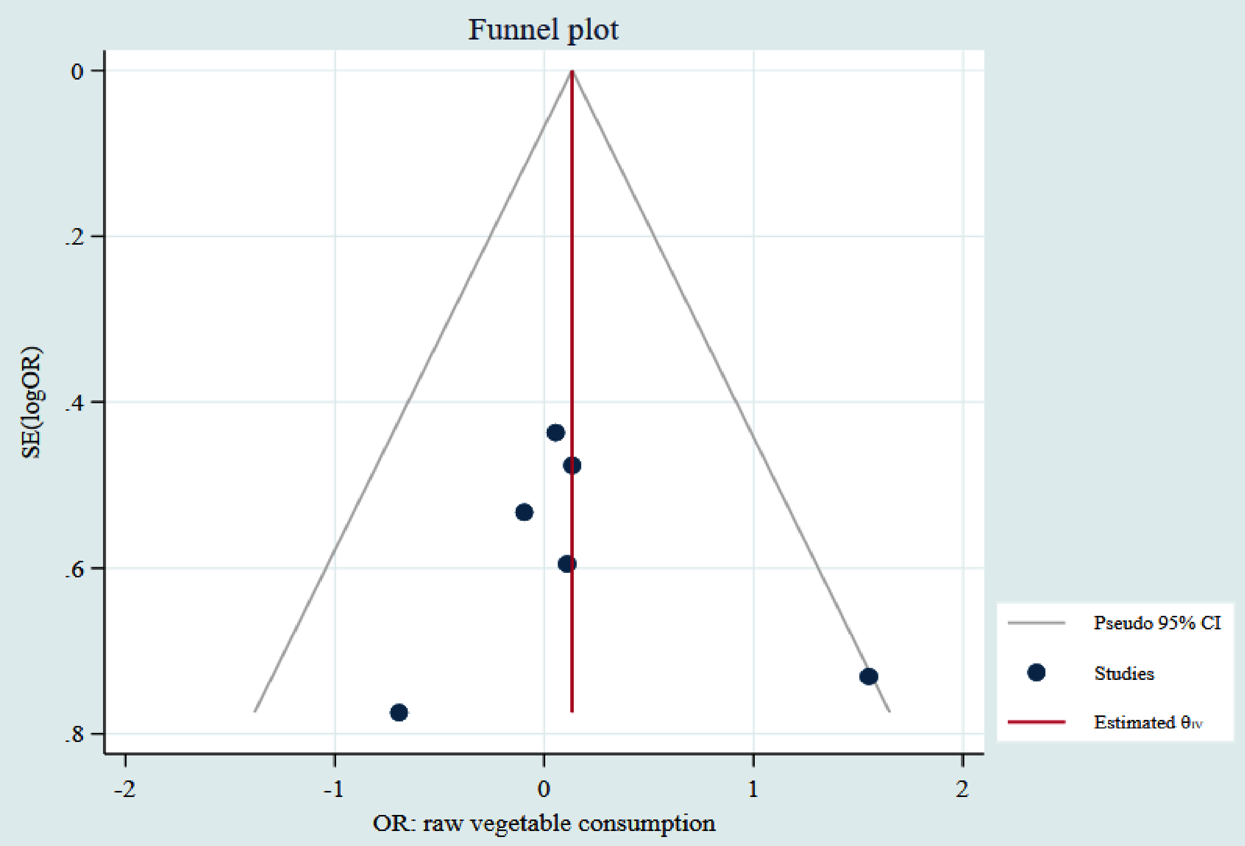

11. Religion (Christian vs Muslim)

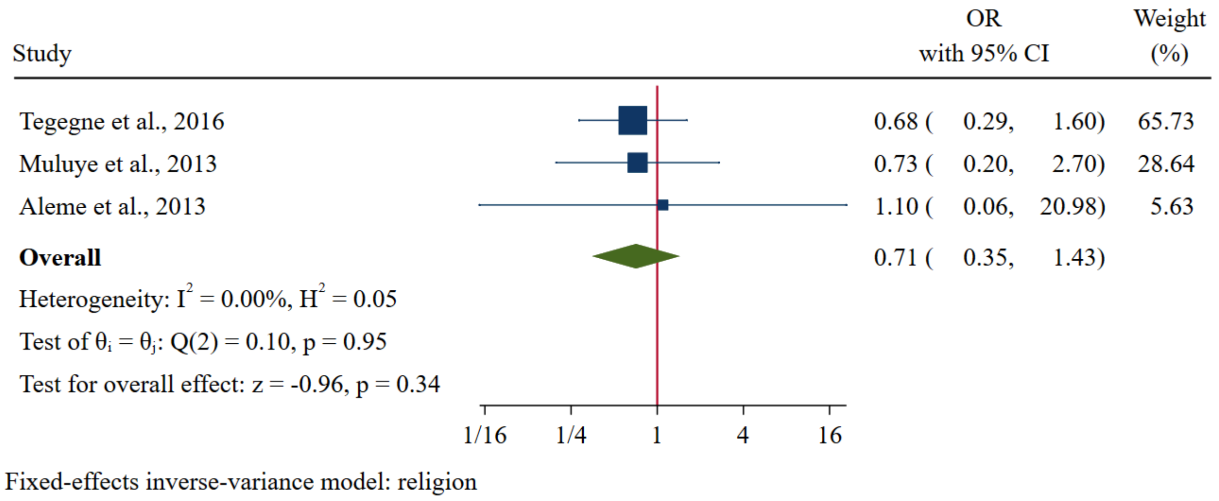

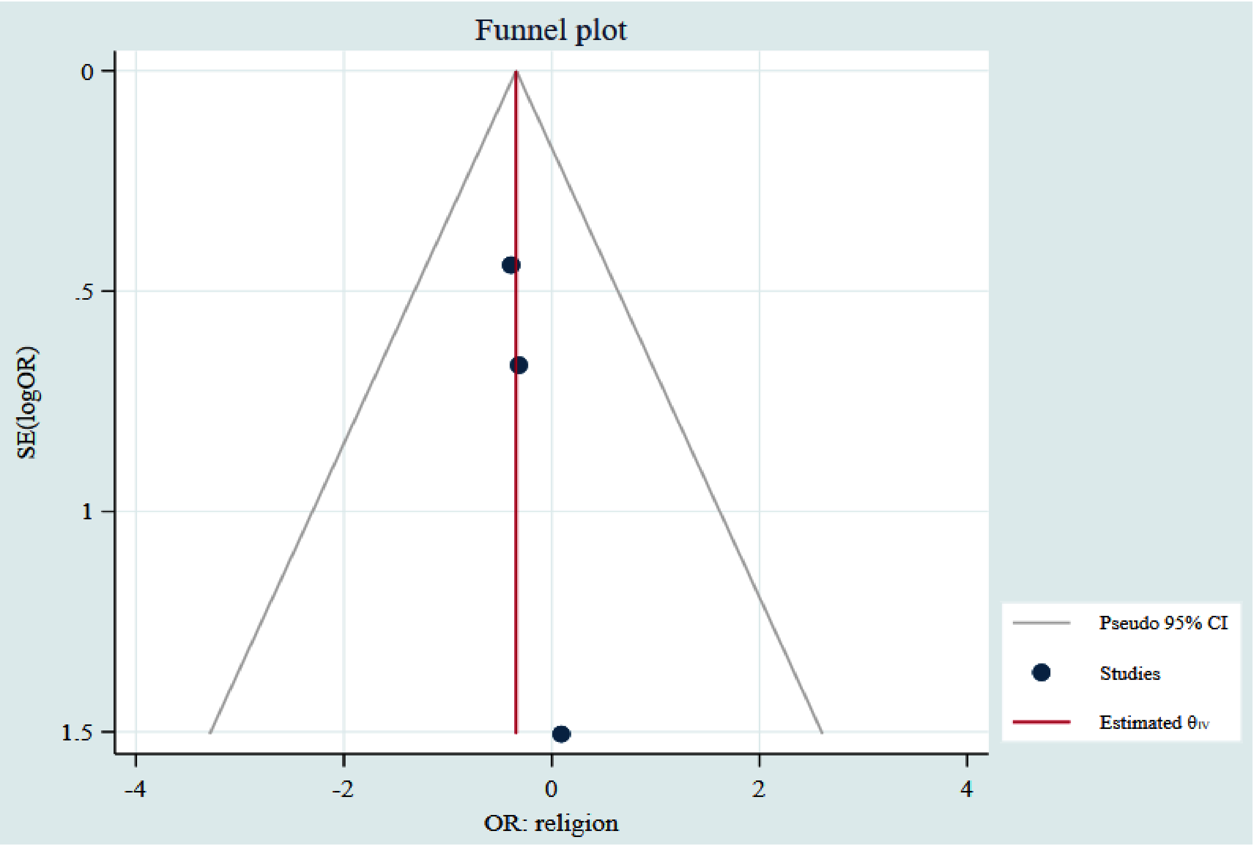

12. <u>Residence (rural vs urban)</u>

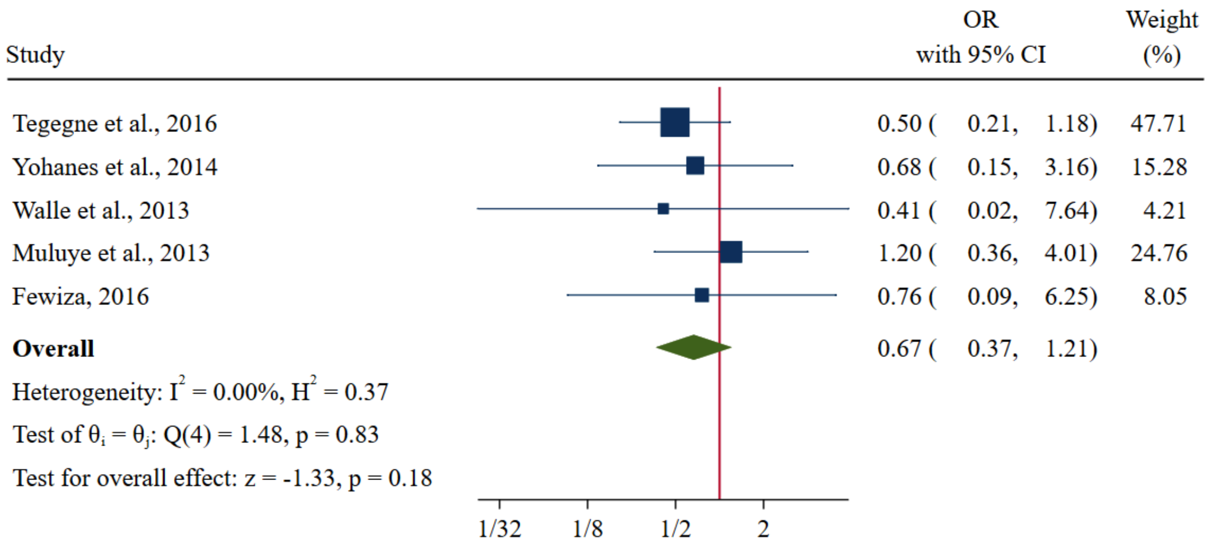

Fixed-effects inverse-variance model: residence

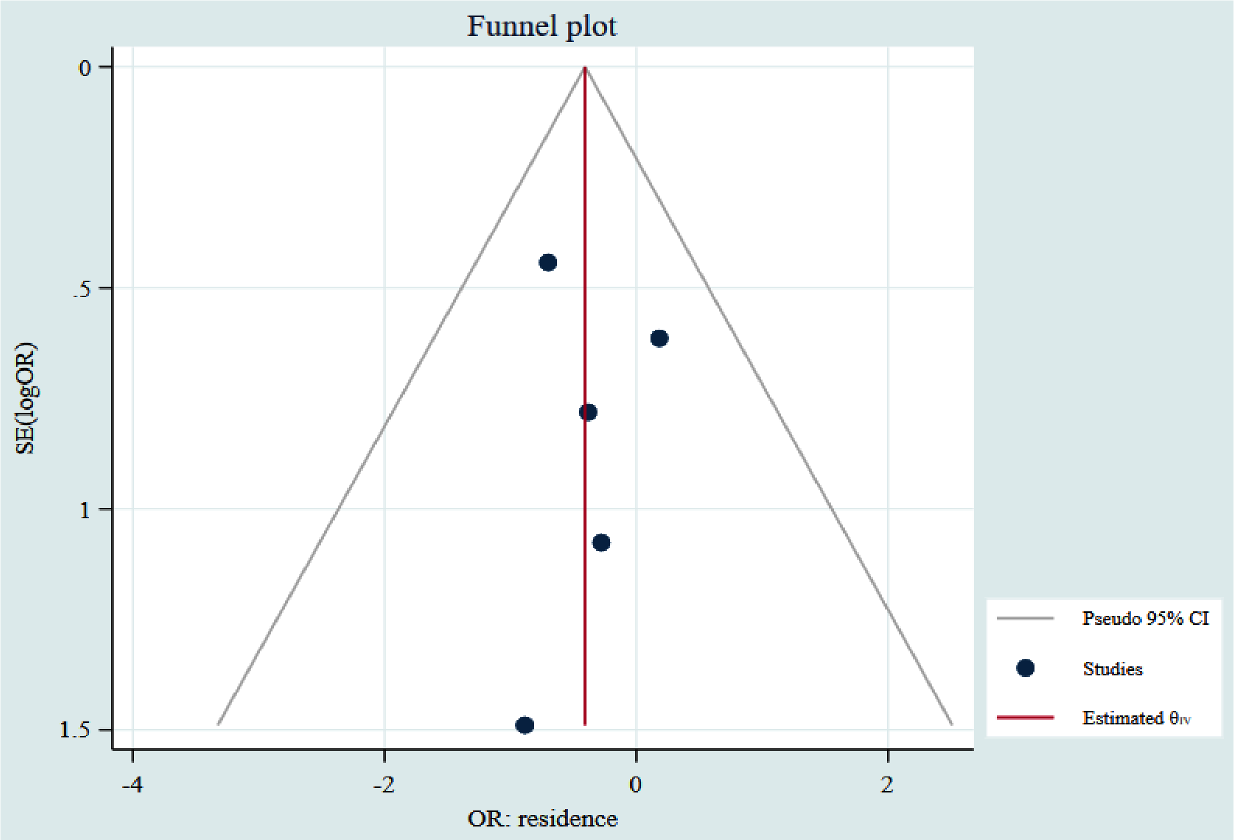

13. Sex (female vs male)

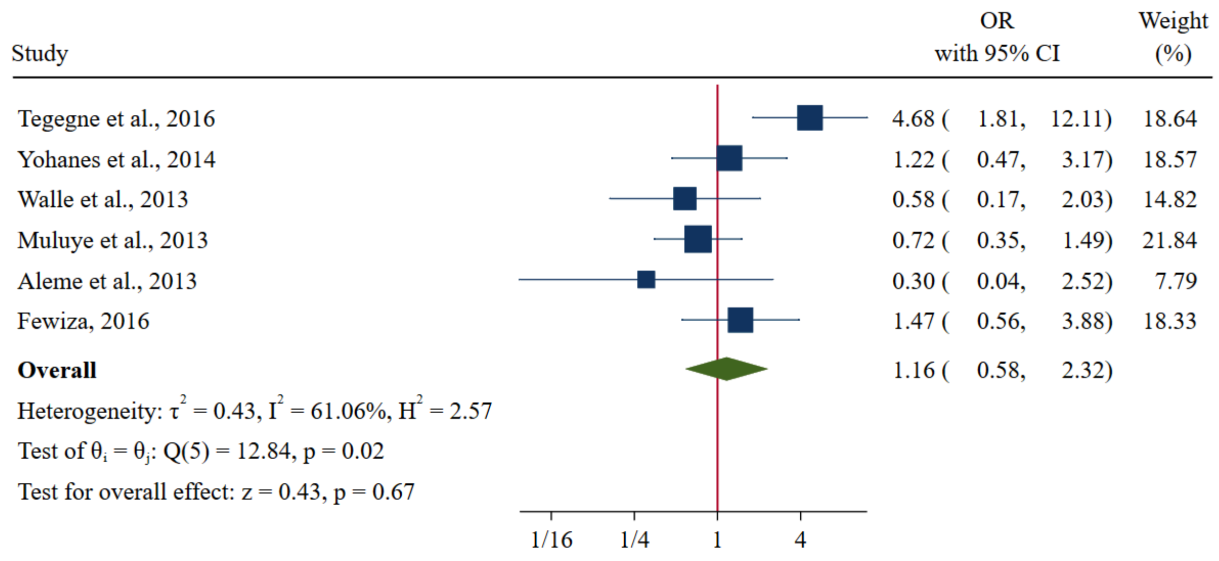

Random-effects DerSimonian-Laird model: sex

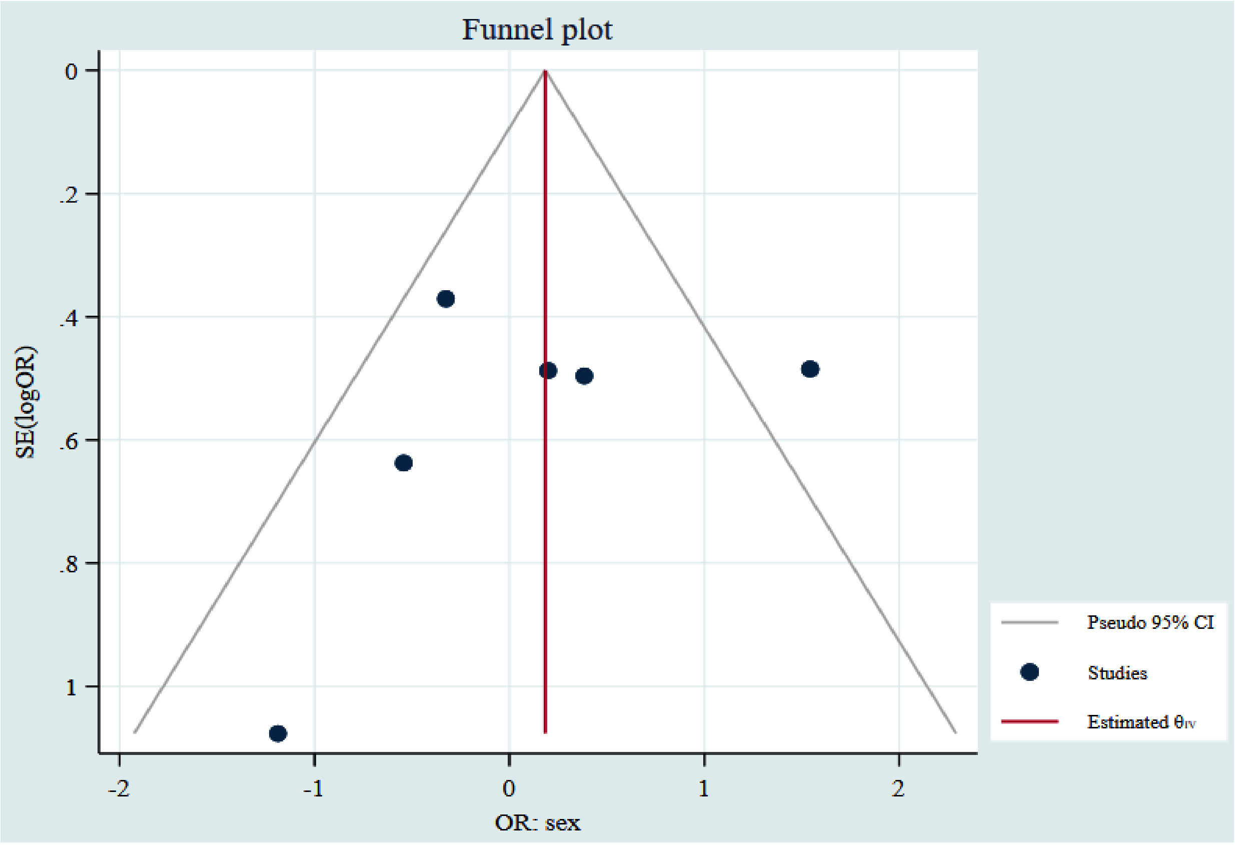

14. Water source quality (unsafe vs safe)

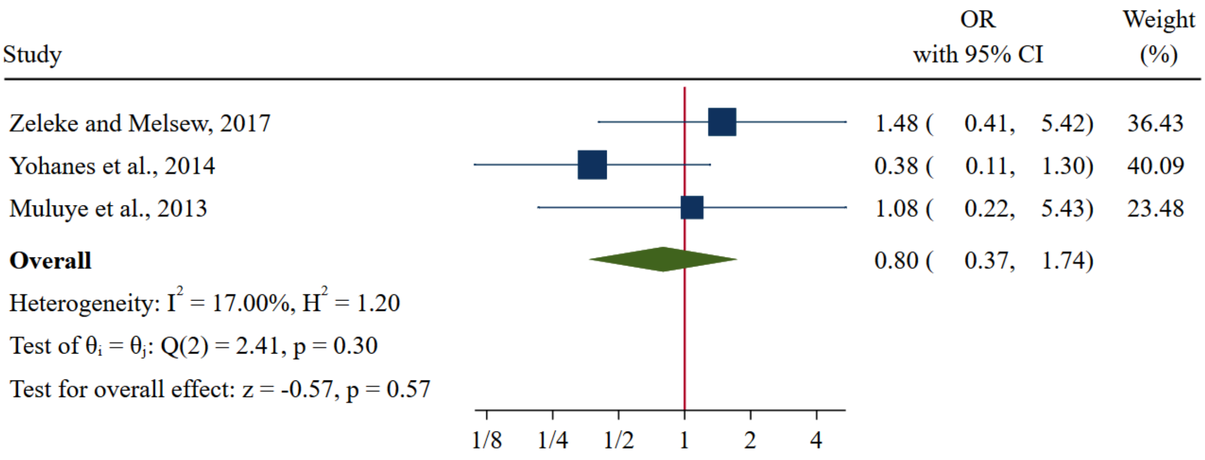

Fixed-effects inverse-variance model: water source

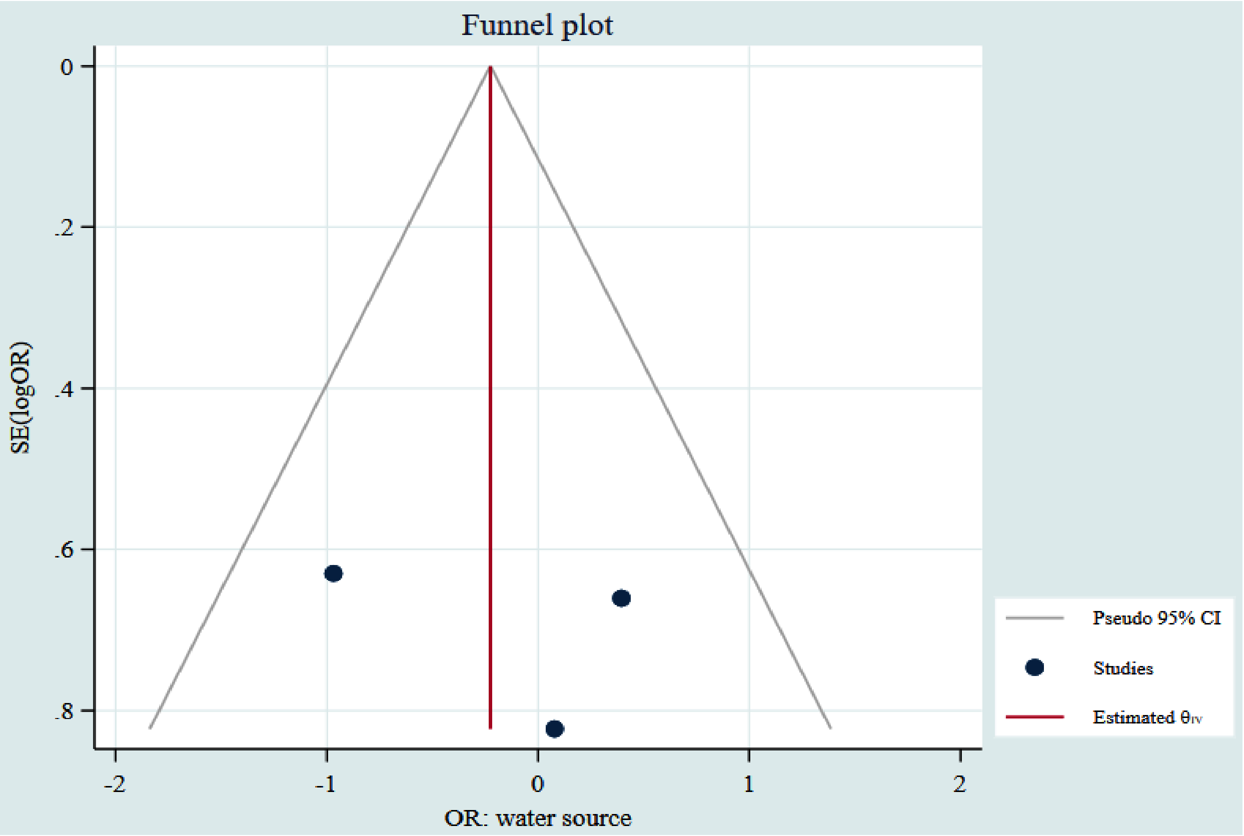

Supplement: S2 Fig — (PDF) [file pntd.0008944.s005.pdf]
