# Supplementary material for: Potential risk factors associated with seropositivity for Toxoplasma gondii among pregnant women and HIV infected individuals in Ethiopia: A systematic review and meta-analysis
Source: PLoS Negl Trop Dis. 2020 Dec 15;14(12):e0008944. doi: 10.1371/journal.pntd.0008944 (PMC7771857; doi:10.1371/journal.pntd.0008944)
Supplement: Attachment — Submitted filename: PNTD-D-20-00491_reviewer_anonymized.pdf [file pntd.0008944.s006.pdf]

PLOS Neglected Tropical Diseases

Potential risk factors associated with Toxoplasma gondii infection in pregnant women and HIV infected individuals in Ethiopia: A systematic review and Meta-analysis

--Manuscript Draft--

**Manuscript Number:**	PNTD-D-20-00491
**Full Title:**	Potential risk factors associated with Toxoplasma gondii infection in pregnant women and HIV infected individuals in Ethiopia: A systematic review and Meta-analysis
**Short Title:**	Potential risk factors associated with Toxoplasma gondii infection in pregnant women
**Article Type:**	Review
**Keywords:**	Ethiopia, Meta-analysis, Potential risk factors, Seroprevalence, Toxoplasma gondii
**Abstract:**	Objectives : We intended to determine the seroprevalence and associated risk factors of Toxoplasma infection in Ethiopian pregnant women and HIV infected individuals.<br>Study design : Systematic review of the previous reports.<br>Methods : We searched PubMed, Science Direct, African Journals Online, and Google Scholar for studies. All references were screened and included if they presented data on at least two risk factors. Meta-analysis using random/fixed effects models was made to calculate the overall effects for each exposure.<br>Results :- This review included 24 papers. The estimated pooled seroprevalence of Toxoplasma infection was found to be 72.5 % in pregnant women and 85.7% in HIV infected individuals. A significant overall effect on Toxoplasma infection in pregnant women ($p < 0.05$) was observed with age, abortion history, contact with cat, cat ownership, having knowledge about toxoplasmosis, being a housewife and having unsafe water source. Age, cat ownership and raw meat consumption have also shown significant effect ($p < 0.05$) on Toxoplasma infection in HIV infected individuals.<br>Conclusion :- This review shows gaps and drawbacks in the prior studies that are useful to keep in mind to design accurate investigations in the future. It proves a high seroprevalence of Toxoplasma infection and appropriate control strategies are recommended. Further study on the risk factors is also required to plan cost-effective preventive strategies.
**Additional Information:**	
**Question**	**Response**
**Financial Disclosure**<br><br>Enter a financial disclosure statement that describes the sources of funding for the work included in this submission. Review the submission guidelines for detailed requirements. View published research articles from *PLOS NTDs* for specific examples.<br><br>This statement is required for submission and **will appear in the published article** if the submission is accepted. Please make sure it is accurate.	The author(s) received no specific funding for this work.

Powered by Editorial Manager® and ProduXion Manager® from Aries Systems Corporation

**Unfunded studies**<br><br>Enter: *The author(s) received no specific funding for this work.*<br><br>**Funded studies**<br><br>Enter a statement with the following details:<br>• Initials of the authors who received each award<br>• Grant numbers awarded to each author<br>• The full name of each funder<br>• URL of each funder website<br>• Did the sponsors or funders play any role in the study design, data collection and analysis, decision to publish, or preparation of the manuscript?<br>• **NO** - Include this sentence at the end of your statement: *The funders had no role in study design, data collection and analysis, decision to publish, or preparation of the manuscript.*<br>• **YES** - Specify the role(s) played.<br><br>* typeset	
**Competing Interests**<br><br>Use the instructions below to enter a competing interest statement for this submission. On behalf of all authors, disclose any competing interests that could be perceived to bias this work—acknowledging all financial support and any other relevant financial or non-financial competing interests.<br><br>This statement **will appear in the published article** if the submission is accepted. Please make sure it is accurate. View published research articles from *PLOS NTDs* for specific examples.	The authors have declared that no competing interests exist.

NO authors have competing interests

Enter: *The authors have declared that no competing interests exist.*

Authors with competing interests

Enter competing interest details beginning with this statement:

I have read the journal's policy and the authors of this manuscript have the following competing interests: [insert competing interests here]

* typeset

Powered by Editorial Manager® and ProduXion Manager® from Aries Systems Corporation

Manuscript Click here to access/download;Manuscript;Potential risk factors.doc

Potential risk factors associated with *Toxoplasma gondii* infection in pregnant women and HIV infected individuals in Ethiopia: A systematic review and Meta-analysis

ABSTRACT

Objectives: We intended to determine the seroprevalence and associated risk factors of *Toxoplasma* infection in Ethiopian pregnant women and HIV infected individuals.

Study design: Systematic review of the previous reports.

Methods: We searched PubMed, Science Direct, African Journals Online, and Google Scholar for studies. All references were screened and included if they presented data on at least two risk factors. Meta-analysis using random/fixed effects models was made to calculate the overall effects for each exposure.

***Results*:-** This review included 24 papers. The estimated pooled seroprevalence of *Toxoplasma* infection was found to be 72.5 % in pregnant women and 85.7% in HIV infected individuals. A significant overall effect on *Toxoplasma* infection in pregnant women ($p < 0.05$) was observed with age, abortion history, contact with cat, cat ownership, having knowledge about toxoplasmosis, being a housewife and having unsafe water source. Age, cat ownership and raw meat consumption have also shown significant effect ($p < 0.05$) on *Toxoplasma* infection in HIV infected individuals.

***Conclusion*:-** This review shows gaps and drawbacks in the prior studies that are useful to keep in mind to design accurate investigations in the future. It proves a high seroprevalence of *Toxoplasma* infection and appropriate control strategies are recommended. Further study on the risk factors is also required to plan cost-effective preventive strategies.

Keywords: Ethiopia, Meta-analysis, Potential risk factors, Seroprevalence, *Toxoplasma gondii*

1. INTRODUCTION

Toxoplasmosis is a food and waterborne protozan infection with high prevalence and distribution in all warm-blooded vertebrates including humans caused by *Toxoplasma gondii*. It causes a considerable public health impact with higher burden in developing countries. Globally, it is estimated to infect approximately 30% of the human population [1]. Infection in healthy individuals is usually asymptomatic, but in immunodeficient patients, it can lead life-threatening encephalitis [2-5]. Further, infection of pregnant women may result in miscarriage or spontaneous abortion or congenital infection that may cause severe pathological effects [5,6]. It also results in personality shifts, epilepsy, bipolar disorder, suicide attempt, car accident and schizophrenia [7,8].

The seroprevalence of toxoplasmosis has been reported ranging from 1% to 100% in humans from various parts of the globe and also shows significant variations within a country or between communities [9]. In Africa, the infection rate ranges from 3% to 92.5% in women of reproductive age and pregnant women [2,6,10]. In Ethiopia, the infection rate estimates of 18.5-96.3% have been reported in the different risk groups of the population [2,11,12]. Various potential factors: sociodemographic and biological factors, lifestyle, climate, socioeconomic status and health care promotion have been documented to effect host- pathogen interaction [13-15]. Thus, control options for toxoplasmosis must rely on the vigorous evidence of the risk factors contributing to its circulation among hosts [16]. However, the relative effects of each noted factors have not been fully summarized, except their variations from area to area [13]. Therefore, this review was made to determine the seroprevalence and potential risk factors (PRFs) of *Toxoplasma* infection in Ethiopian pregnant women and HIV infected individuals.

2. METHODS

2.1. Search strategy

Literature searching was performed following the PRISMA guideline (Sup 1 File) and its checklist (STROBE) (Sup2 File) [17,18]. We searched PubMed, Science Direct, African Journals Online, and Google scholar databases with no restiriction on year up to 30th November 2019. Article search was made using the MeSH terms: (Toxoplasmosis OR *Toxoplasma* infection OR *Toxoplasma gondii* OR *T. gondii* OR *Toxoplasma*) AND

(Seroprevalence OR Prevalence OR Seroepidemiology) AND (Risk factors OR Potential factors) AND (Pregnant women) AND (Ethiopia). Also, keywords: (HIV infected individuals OR HIV/AIDS patients) were used. Moreover, unpublished thesis manuscripts were also accessed from Ethiopian Universities.

2.2. Inclusion criteria

We used the following inclusion criteria to confirm the eligibility of the searched papers: (1) original research articles and thesis; (2) cross-sectional studies that were reported: seroprevalence and risk factors; (3) studies with full texts; (4) targeted study population: pregnant women and HIV infected individuals; (5) studies with serological tests; (6) studies that provided the total sample size and the outcome of interest; and (7) studies published in English language. Each paper that did not meet the above-mentioned criteria was excluded.

2.3. Data extraction

Initially, articles were screened based on their titles and abstracts following the predefined inclusion criteria. Then, articles that seem potential for eligibility were selected and downloaded for full text. The searched articles were reviewed and abstracted carefully by two independent reviewers to prove the eligibility. Disagreements between reviewers were settled by discussion. For each article, the following information were extracted: first author, publication year, study year, location, study design, sampling method, sample size, study subject, diagnostic test, potential risk factors (exposure), number of positive and negative samples. The study effect size, odds ratio and their corresponding confidence intervals were also calculated from the extracted data. Study searching strategies and exclusion criteria are presented in detail in Fig 1**.**

2.4. Meta-analysis

The meta-analysis was performed according to the protocol noted by Foroutan-Rad et al. [19] and Khalkhali et al. [20]. In brief, data on pregnant women and HIV infected individuals were analyzed separately. The seroprevalence and corresponding 95% confidence intervals (CIs) of toxoplasmosis were calculated for each study. The pooled seroprevalence estimate was computed according to Barendregt et al. [21]. Also, meta-analyses

were made to determine the effects of each identified PRF if at least two included studies reported data on the same risk factor. OR and its respective 95% CI were calculated for each risk factor. We used pooled odds ratios (OR) as a measure of effect to assess the overall effects of each PRF. Forest plot was employed to present the outcomes of meta-analysis. Cochran's Q-statistics and inverse variance index (I^2) were computed to determine the heterogeneity and inconsistency among studies, respectively [2]. Galbraith plot was also constructed to assess the heterogeneities of study level estimates. We used random-effect model (if the p-value of the Q test was < 0.05 and I^2 was >50%) or fixed-effect model to pool the estimations in accordance with the heterogeneity result. Besides, we used random-effect model for risk factor assessment if we had five and above studies to accept the previous cut-off points [23]. Further, funnel plot, Egger's and Begg's tests [24] were performed to assess small study effects and publication bias. STATA software version 16 (StataCorp, College Station, TX, USA) was used for meta-analyses.

3. RESULTS

3.1. Search results and characteristics

We retrieved 216 reports for *Toxoplasma* infection in pregnant and HIV infected individuals. Of them, only 24 were found to be eligible for the inclusion criteria and data extraction (Fig 1). All selected studies were performed between 2007 and 2019 using a cross-sectional study design with convenient, systematic and simple random sampling procedures. Further, the studies were conducted using latex agglutination test(LAT), Enzyme-linked immunosorbent assay (ELISA) and Enzyme immune assay(EIA) to detect *Toxoplasma* antibodies (Table 1 and Table 2). The total sample size of the included studies was 6003 individuals (4356 pregnant women and 1647 HIV infected individuals). Of them, 4243 were found to be seropositive for *Toxoplasma* infection. The overall apparent infection rate in pregnant women and HIV infected individuals was 66% and 84%, respectively.

Table 1: List of included studies in meta-analysis on pregnant women

Author	Study Year	Geographical Location	Regional State	Sampling method	Diagnostic test	Sample Size	Events	Event rate (AP)	LCI	UCI
Biyansa [25]	2019	N.West Ethiopia	Amhara	Systematic	LAT	401	284	0.71	0.66	0.75
Teweldemedhin et al. [26]	2018	Northern Ethiopia	Tigray	Simple	ELISA	360	128	0.36	0.31	0.41
Jula et al. [27]	2015	Southern Ethiopia	Oromia	Simple	EIA	401	96	0.24	0.20	0.28
Ahmed et al. [28]	2012	Central Ethiopia	Addis Ababa	Systematic	ELISA	192	169	0.88	0.83	0.93
Negero et al. [29]	2016	S.West Ethiopia	SNNPR	Systematic	LAT	210	159	0.76	0.7	0.82
Negussie et al. [30]	2014	S.East Ethiopia	Somali	Convenient	LAT	301	201	0.67	0.61	0.72
Yohanes et al. [31]	2015	Southern Ethiopia	SNNPR	Systematic	ELISA	232	184	0.79	0.74	0.85
Abamecha and Awel [32]	2015	S.West Ethiopia	SNNPR	Systematic	ELISA	232	198	0.85	0.81	0.9
Agmas et al. [33]	2013	N.West Ethiopia	Amhara	Systematic	LAT	263	180	0.68	0.63	0.74
Gelaye et al.[34]	2010	Central Ethiopia	Addis Ababa	Convenient	LAT	288	246	0.85	0.81	0.89
Awoke et al.[11]	2013	N.West Ethiopia	Amhara	Simple	LAT	384	71	0.18	0.15	0.22
Hailu et al.[35]	2012	Selected parts of Ethiopia	Selected parts of Ethiopia	Simple	ELISA	293	256	0.87	0.84	0.91
Endris et al.[36]	2011	N.West Ethiopia	Amhara	Convenient	LAT	385	341	0.89	0.85	0.92
Gebremedhin et al.[37]	2011	Central Ethiopia	Selected parts of Ethiopia	Simple	ELISA	213	184	0.86	0.82	0.91
Zemene et al.[38]	2011	S.West Ethiopia	Oromia	Systematic	ELISA	201	168	0.84	0.78	0.89

Table 2: List of included studies in meta-analysis on HIV infected individuals

Author	Study Year	Geographical Location	Regional State	Sampling method	Diagnostic test	Sample Size	Events	Event rate (AP)	LCI	UCI
Fewiza [39]	2016	Central Ethiopia	Addis Ababa	Convenient	ELISA	174	99	0.57	0.5	0.64
Zeleke and Melsew [40]	2015	S.West Ethiopia	SNNPR	Systematic	ELISA	270	255	0.94	0.92	0.97
Tegegne et al.[41]	2015	S.West Ethiopia	Oromia	Convenient	LAT	135	109	0.81	0.74	0.87
Yesuf and Melese [42]	2014	S.West Ethiopia	Oromia	Systematic	-	120	72	0.6	0.51	0.69
Hailu et al.[35]	2012	Selected areas of Ethiopia	Selected areas of Ethiopia	Simple	ELISA	190	178	0.94	0.9	0.97
Yohanes et al.[43]	2013	Southern Ethiopia	SNNPR	Simple	ELISA	170	150	0.88	0.83	0.93
Walle et al.[44]	2011	N.West Ethiopia	Amhara	Convenient	ELISA	103	90	0.87	0.81	0.94
Muluye et al.[45]	2012	Northern Ethiopia	Amhara	Systematic	LAT	170	130	0.76	0.7	0.83
Aleme et al.[46]	2011	Central Ethiopia	Addis Ababa	Simple	ELISA	150	141	0.94	0.9	0.98
Shimelis et al.[47]	2007	Central Ethiopia	Addis Ababa	Systematic	ELISA	165	154	0.93	0.9	0.97

3.2. Meta-analysis and bias assessment

The random effect model with inverse variance procedures showed an overall pooled seroprevalence of 72.5 % in pregnant women and 85.7% in HIV infected individuals. A significant considerable heterogeneity of studies in pregnant women was evidenced ($I^2 = 98.6\%$, $p < 0.001$). Similarly, in HIV infected individuals, the heterogeneity of studies was statistically significant ($I^2 = 94.8\%$, $p < 0.001$). The forest plots for each study groups are shown in Fig 2 and 3. The Galbraith plot assessment in pregnant women and HIV infected individuals (Fig 4) also revealed that most of the included reports are laid outside of the 95% confidence limit and provided clear evidence on the variability of reports.

In our review, Funnel plot observations (Fig 5, 6 & 7) and the bias coefficients in both pregnant women (Egger's test: b= 26.24, 95%CI: 10.13-42.35, $p = 0.004$ and Begg's test: $p = 0.015$) and HIV infected individual (Egger's test: b= 14.8, 95%CI: 9.44-20.2, $p < 0.001$ and Begg's test: $p = 0.016$) studies did confirm the presence of publication bias and small-study effects.

3.3. Potential risk factors with *T. gondii* infection in pregnant women

Nineteen potential risk factors (PRF) were identified and meta-analysis was made on thirteen of the fifteen included papers (Table 3). Of them, seven were shown a statistically significant effect on *Toxoplasma* infection (test for overall effect, $p < 0.05$) with higher odds of outcome: "history of abortion" (OR: 1.52), "age ≥ 35 years" (OR: 2.93), "contact with cat" (OR: 1.50), "cat ownership" (OR: 2.35), "knowledge about toxoplasmosis" (OR: 0.13), "being house wife" (OR: 1.58) and "unsafe water source" (OR: 1.55) as shown in Table 3 and Sup 3.

An increased odds of infection was also observed in eight assessed PRFs with *Toxoplasma* infection in pregnant women; though these were not statistically significant ($p > 0.05$): "blood transfusion" (OR: 1.13), "contact with soil" (OR: 1.1), "dog ownership" (OR: 1.21), "being HIV positive" (OR: 1.22), "more than one pregnancy" (OR: 1.30), "raw meat consumption" (OR: 1.23), "raw vegetable consumption" (OR: 1.36) and "living in rural areas"

(OR: 1.46). Further, we were not able to determine the effect of educational status, farming activity, raw milk consumption and religion on odds of infection in pregnant women (Table 3). Forest plots, funnel plots and single weight of each publication contributing to the overall risk factors are presented in Sup 3 File.

Table 3: Potential risk factors and their overall effects on *Toxoplasma* seroprevalence in pregnant women

Potential risk factors	Effect model	OR(95%CI)	Overall effect	Num. studies	References (p < 0.05)	References (p > 0.05)
**History of abortion**	**IV, Fixed**	**1.52(1.15,2.00)**	***p* < 0.0001**	**6**	[11,29]	[25,26,30,34]
**Age (≥35years)**	**IV, Random**	**2.93(1.73,4.95)**	***p* < 0.0001**	**8**	**[26,29,33]**	**[11,27,28,31,34]**
Blood transfusion	IV, Fixed	1.13(0.73,1.73)	*p* = 0.59	6		[11,25,26,29,31,34]
**Contact with cat**	**IV, Fixed**	**1.50(1.04,2.14)**	***p* = 0.03**	**3**	**[32,33]**	**[27]**
**Cat ownership**	**IV, Random**	**2.35(1.44,3.85)**	***p* < 0.0001**	**12**	**[11,26,28,29,33,36,38]**	**[25,30, 31,32,34]**
Contact with soil	IV, Fixed	1.1(0.73,1.58)	*p* = 0.72	4	[32]	[29,33, 38]
Dog presence	IV, Fixed	1.21(0.86,1.71)	*p* = 0.28	3		[25,31,36]
Being illiterate	IV, Fixed	1.02(0.83,1.24)	*p* = 0.87	12	[26,32,33]	[11,25,27,29,30,31,34,36,38]
Farming/gardening activity	IV, Fixed	0.70(0.46,1.08)	*p* = 0.10	2		[30,31]
HIV status	IV, Fixed	1.22(0.70,2.13)	*p* = 0.47	5		[28,29,30,34,36]
**Knowledge on toxoplasmosis**	**IV, Fixed**	**0.13(0.02,0.66)**	***p* = 0.01**	**2**		**[27,28]**
Number of pregnancy	IV, Random	1.30(0.90,1.88)	*p* = 0.17	7	[28,29]	[11,25,32,29,31]
**Being housewife**	**IV, Random**	**1.58(1.12,2.22)**	***p* = 0.01**	**10**	**[26,33]**	**[25,27-32,34]**
Raw meat consumption	IV, Random	1.23(0.77,1.98)	*p* = 0.38	12	[11,28,29,31,32]	[25,26,27,30,34,36,38]
Raw milk consumption	IV, Fixed	0.76(0.57,1.01)	*p* = 0.06	4		[25-27,29]
Raw vegetable consumption	IV, Random	1.36(0.88,2.12)	*p* = 0.17	9	[26,28,30,31]	[11, 25,27,30,34]
Christianity vs Muslim	IV, Fixed	1.03(0.61,1.73)	*p* = 0.91	3		[28,30,36]
Residence: rural	IV, Random	1.46(0.96,2.23)	*p* = 0.08	10	[26,29,33]	[11,25,27,30,31,32,36]
**Unsafe water source**	**IV, Fixed**	**1.55(1.21,1.99)**	***p* < 0.0001**	**9**	**[26,27,29]**	**[11,25,31,33,36,38]**

3.4. Potential risk factors with *T. gondii* infection in HIV infected individuals

Thirteen potential risk factors were identified and meta-analysis was performed on seven of the included ten papers (Table 4). Of them, three were shown a statistically significant combined effect on *Toxoplasma* infection (test for overall effect, $p < 0.05$): "age ≥25 years" (OR: 3.087), "cat ownership" (OR: 4.34) and "raw meat consumption" (OR: 2.43). On the other hand, six risk factors appeared to have higher odds of infection but results have shown statistically insignificant overall effect ($p > 0.05$): "contact with cat" (OR: 1.37), "living as single" (OR: 1.20), "being illiterate" (OR: 1.1), "raw vegetable consumption" (OR: 1.14), "living in rural" (OR: 1.502) and "being female" (OR: 1.16) (Table 2). Besides, four risk factors: having "blood transfusion experience", having "knowledge about toxoplasmosis", "religion", and having "unsafe drinking water source" were not appeared to influence the odds of infection in HIV infected individuals (Table 4). Forest plots, funnel plots and single weight of each publication contributing to the overall risk factors are presented in Sup 4 File.

Table 4: Potential risk factors and their overall effects on *Toxoplasma* seroprevalence in HIV infected individuals

**Potential risk factor**	**Effect model**	**OR(95%CI)**	**Overall effect**	**Num. studies**	**References reported (p < 0.05)**	**References reported (p > 0.05)**
**Age (≥25yr)**	**IV, Fixed**	**3.087(1.36,6.99)**	***p* = 0.0068**	**2**	[**43**]	[**41**]
Blood transfusion	IV, Fixed	0.98 (0.42,2.32)	*p* = 0.97	4		[39,40,43,44**]**
**Cat ownership**	**IV, Fixed**	**4.34(2.49,7.56)**	***p* < 0.0001**	**4**	[**39,41]**	[**43,46]**
Contact with cat	IV, Fixed	1.37(0.74,2.54)	*p* = 0.31	3	[44**]**	[40,45**]**
Being illiterate	IV, Fixed	1.1(0.68,1.65)	*p* = 0.81	6		[40, 41, 43-46**]**
Knowledge on toxoplasmosis	IV, Fixed	0.89(0.35,2.28)	*p* = 0.81	2	[41,46**]**	
Marital status	IV, Fixed	1.20(0.70,2.08)	*p* = 0.51	3		[43,45,46**]**
**Raw meat consumption**	**IV, Random**	**2.43(1.23,4.79)**	***p* = 0.01**	**7**	[**41,43,44]**	[**39,40,45,46]**
Raw vegetable consumption	IV, Fixed	1.14(0.73,1.78)	*p* = 0.56	6	[46**]**	[39,40,41,43,44**]**
Religion (Christian)	IV, Fixed	0.71(0.35,1.43)	*p* = 0.34	3		[41,45,46**]**
Residence (rural)	IV, Fixed	1.50(0.83,2.73)	*p* = 0.18	5		[39,41,43-45**]**
Sex (female)	IV, Fixed	1.16(0.58,2.32)	*p* = 0.67	6	[41**]**	[39,43-46**]**
Unsafe water source	IV, Fixed	0.80(0.37,1.74)	*p* = 0.57	3		[40,43,45**]**

4. DISCUSSION

4.1. Pooled seroprevalence of *Toxoplasma* infection

The present study revealed that the overall pooled *Toxoplasma* seroprevalence in Ethiopian pregnant women and HIV infected individuals was found to be 72.5% and 85.7%, respectively. This suggests that the probability of high risk for congenital and cerebral toxoplasmosis in such groups, respectively. Higher *Toxoplasma* seroprevalence was reported in previous studies in Ethiopia: 81.4% in women of childbearing age [37], 74.2% in HIV infected individuals [48] and 33.6% in psychiatric patients [49]. Seroprevalence reports for the general population of Ethiopia were also found to be 60-74.4% [12,50,51]. Comparable findings were also reported across the world [52-58]. On contrary, our report is higher than the previous findings in Africa [6,59-70]. Further, significantly lower seroprevalence reports were also documented in other continents [5,15,71-82]. This variation among countries may be due to various risk factors: local climatic which determines the survival of oocysts and favours the dissemination and sporulation of oocysts, nutritional habit, personal hygiene, sources of drinking water, socio-cultural differences, residence, cat density and management, and levels of close contact with cats [4,5,13,54,57,67]. Several studies have also shown the effects of close contact with infected meat, consumption of raw meat, vegetable and contaminated water [4,19,72,77,83,84]. Further, infection rate also depends on education level, socioeconomic status, and local religious belief of the community [4,5,54,77,85,86].

4.2. Potential risk factors analysis

We observed significantly higher odds of *Toxoplasma* infection in pregnant women and HIV infected individuals as the study subjects' age increases. This agrees with studies that reported higher *Toxoplasma* seroposetivity in an older groups than youngs [5,16,54,83,87]. Increased seroprevalence in older groups was also confirmed [9,15,52,64,88-90]. Studies included in this review were also considered age as a potential factor [26,29,33,43] This might be explained by the assenting interaction between an increase in age, with a prolonged risk of exposure to *Toxoplasma* oocysts over time, with divers transmission route, lack of community awareness and a

long-lived immune response [15,91]. This could also be due to that older groups could have a longer period of exposure to any of the risk factors [60,92].

Toxoplasma seropositivity has shown an association with history of abortion ($p < 0.05$). This is in accordance with the previous reports [60,93,94]. Being cat ownership, close contact with cats and abundance of cats are also considered to be important drivers for *Toxoplasma* infection [13,54,90]. Domestic cats are important sources of viable oocysts for environmental contamination [5,90]. Cats after having a single viable tissue cyst or bradyzoite can release millions of viable oocysts and augment the likelihood of pathogen transmission to the risk groups [19,83]. In this review, ownership of cats was found with significantly higher combined odds ratio in both study targets. Contact with cats was also found with significantly higher odds ratio in pregnant women. This suggests that ownership of cats or close contact with cats in couple with frequent exposure to cat faeces, cats' litter box management way or neglect of preventive measures (hand washing or wearing gloves) could increase the risk of infection to appreciable level [13,15,19,37,77,90].

Toxoplasma infection in high-risk groups such as pregnant women, HIV infected individuals and cancer patients has been reported to be influenced by educational status and knowledge about toxoplasmosis [54,90]. Sun et al. [95] and Liu et al. [77] also stated that individuals with no knowledge and formal education are more likely to acquire *Toxoplasma* infection. Other authors suggest that health education as cost-effective intervention [13,77,90]. This is supported by our findings in which literacy and health knowledge are considered as protective factors. Similar findings also reported by other authors [40,41,77,83]. This might be attributed to that those with low/no formal education status may have less hygienic practice and they are more likely to acquire *Toxoplasma* infection [54,77]. Lack of basic information about toxoplasmoses such as a source of infection, hygiene, raw meat/vegetable consumption, transmission route, and ignorance of the disease can maintain the risk of infection.

Previous reports have pointed out that *Toxoplasma* seroprevalence is varied among various working groups. Certain working groups have more contact than others with *Toxoplasma* directly or indirectly. The present meta-

analysis reveals that housewives are a more vulnerable group with significantly higher *Toxoplasma* seroprevalence. Our results are in line with those studies done in Saudi Arabia and the United States [72,96,97]. Habitually, housewives spend more time in taking care of pets, cooking and tasting food at home during meal preparation, handling and chopping meat without wearing gloves, cleaning and washing vegetables, and engaging in gardening, mainly in rural regions [15,83].

Published reports have documented that unhygenic drinking water is a considerable risk factor for *Toxoplasma* infection in human and animals [13,90]. In our review, unsafe drinking water has also shown a significant association with *Toxoplasma* infection in pregnant women but not in HIV infected individuals. This might be due to the contradicting findings of the included studies and wide confidence intervals. Hence, we considered the absence of tap water as an indicator of unsafe drinking water quality. However, this might not be necessarily true. The microbiological quality of well or spring water could be satisfactory for the majority of the time unless contamination events happen by flooding and contaminated individuals/animals [98,99].

Higher odds of *Toxoplasma* infection were confirmed in pregnant women with risk factors like raw meat and vegetable consumption; though it was not significant. Similarly, raw meat consumption habit has shown significantly higher odds of *Toxoplasma* infection in HIV infected individuals (p = 0.01). This is consistent with reports across the globe [34,77,90,100-102]. The consumption of raw meat with tissue cysts and vegetables contaminated with sporulated oocysts are considerable sources of infection [77]. The risks of acquiring *Toxoplasma* via contaminated meat with tissue cysts and vegetable with oocysts vary with cultural and feeding habits in various communities [13].

This systematic and meta-analysis has certain limitations, including: (1) all studies were used cross-sectional study design, this may bias our risk factor analysis, (2) all studies that exploring seroprevalence/ factors were drawn from limited samples of participants which may not represent the national seroprevalence, (3) lack of related risk factors evaluated by the majority of papers, (4) the number of eligible studies used for each risk factor

analysis were small; so the estimates and the predictive values of the risk factors were assessed accordingly; it might decrease the power of meta-analysis, (5) limited or lack of studies in many regions of the country and (6) lack of a standard questionnaire to gather suitable data in the country.

In conclusion, the results of this meta-analysis show that pregnant women and HIV infected individuals have a high burden of *Toxoplasma* infection. The main inference of our results is that routine serological screening tests for *Toxoplasma* infection coupled with health promotion should be made to the community. This review also identified various important risk factors associated with *Toxoplasma* infection. Cat contact and ownership are relevant for both developed and developing countries and prevention measures should include awareness campaigns and better hand hygiene. By considering the significance of toxoplasmosis in pregnant women and HIV infected individuals, strategies to reduce the prevalence and burden of toxoplasmosis and other diseases should be prioritised and offered adequate funding.

Authors statement

Ethical approval

None sought

Funding

This work not supported by any funding agency.

Competing interests

We declare that no conflict of interests.

Availability of data and materials

All data generated during this study are included as supplementary information.

Supplementary files and tables

Sup 1 File: PRISMA 2009-checklist

Sup 2 File: STROBE Statement-checklist

Sup 3 File: Potential factors reported for toxoplasmosis in pregnant women of Ethiopia

Sup 4 File: Potential factors for toxoplasmosis in HIV infected individuals of Ethiopia

5. REFERENCES

1. Foroutan M, Dalvand S, Daryani A, Ahmadpour E, Majidiani H, Khademvatan S, et al. Rolling up the pieces of a puzzle: A systematic review and meta-analysis of the prevalence of toxoplasmosis in Iran. *Alex J Med* 2018; **54**: 189-196.
2. Dubey JP, Tiao N, Gebreyes WA, Jones JL. A review of toxoplasmosis in humans and animals in Ethiopia. *Epidemiol Infect* 2012; **140**: 1935–1938.
3. Wang Z, Liu H, Ma Z, Ma H, Li Z, Yang Z, et al. *Toxoplasma gondii* infection in immunocompromised patients: A systematic review and meta-analysis. *Front Microbiol* 2017a; **8**: 1–12.
4. Wang Z, Wang S, Liu H, Ma H, Li Z, Wei F, et al. Prevalence and burden of *Toxoplasma gondii* infection in HIV-infected people: a systematic review and meta-analysis. *Lancet HIV* 2017b; **4**: e177–e188.
5. Jiang RL, Ma LH, Ma ZR, Hou G, Zhao Q, Wu X. Seroprevalence and associated risk factors of *Toxoplasma gondii* among Manchu pregnant women in northeastern China. *Microb Pathog* 2018;**123**: 398–401.
6. Paul E, Kiwelu I, Mmbaga B, Nazareth R, Sabuni E, Maro A, et al. *Toxoplasma gondii* seroprevalence among pregnant women attending antenatal clinic in Northern Tanzania. *Trop Med Health* 2018; **46**: 1–8.
7. Dogruman-Al F, AslantS, AlcanS, Customer S, Turk S. A possible relationship between *Toxoplasma gondii* and schizophrenia: a seroprevalence study. *Int J Psychiatr Clin Pract* 2009; **13**:82–7.

8. Chegeni TN, Sarvi S, Amouei A, Moosazadeh M, Hosseininejad Z, Aghayan SA, et al. Relationship between toxoplasmosis and obsessive-compulsive disorder: A systematic review and meta-analysis. *PLoS Negl Trop Dis* 2019; **13**(4): e0007306.

9. Alzaheb R A. Seroprevalence of *Toxoplasma gondii* and its associated risk factors among women of reproductive age in Saudi Arabia: a systematic review and meta-analysis. *Int J Women Health* 2018;**10**: 537-544.

10. Hammond-Aryee K, Esser M, Van Helden PD. *Toxoplasma gondii* seroprevalence studies on humans and animals in Africa. *S Afr Fam Pract* 2014; **56**(2): 119-124.

11. Awoke K, Nibret E, Munshea A. Sero-prevalence and associated risk factors of *Toxoplasma gondii* infection among pregnant women attending antenatal care at Felege Hiwot Referral Hospital, northwest Ethiopia. *Asian Pac J Trop Med* 2015; **8**: 549–554.

12. Tilahun B, Hailu Y, Tilahun G, Ashenafi H, Vitale M, Di Marco V et al. Seroprevalence and risk factors of *Toxoplasma gondii* infection in humans in East Hararghe Zone, Ethiopia. *Epidemiol Infect* 2016; **144**: 64–71.

13. Elsheikha HM. Congenital toxoplasmosis: Priorities for further health promotion action: a review paper. *J R Inst Pub Health* 2008; **122**: 335-353.

14. Dubey JP. Toxoplasmosis, 2nd ed. CRC Press Taylor & Francis Group, New York; 2010

15. Mizani A, Alipour A, Sharif M, Sarvi S, Amouei A, Shokri A, Daryani A et al. Toxoplasmosis seroprevalence in Iranian women and risk factors of the disease: a systematic review and meta-analysis. *Trop Med Health* 2017; **45**(1): 7.

16. Ding H, Gao Y, Deng Y, Lamberton PHL, Lu D. A systematic review and meta-analysis of the seroprevalence of *Toxoplasma gondii* in cats in mainland China. *Parasit Vect* 2017; **10**: 27.

17. Moher D, Liberati A, Tetzlaff J, Altman DG. The PRISMA Group: Preferred Reporting Items for Systematic Reviews and Meta-Analyses: The PRISMA Statement. *Int J Sur* 2010; **8**: 336-341.

18. von Elm E, Altman DG, Egger M, Pocock SJ, Gotzsche PC, Vandenbroucke JP. STROBE Initiative. The strengthening the Reporting of Observational Studies in Epidemiology (STROBE) statement: guidelines for reporting observational studies. Prev Vet Med 2007; **45**(4): 247-51.

19. Foroutan-Rad M, Khademvatan S, Majidiani H, Aryamand S, Rahim F, Malehi A. Seroprevalence of *Toxoplasma gondii* in the Iranian pregnant women: A systematic review and meta-analysis. *Acta Trop* 2016; **158**: 160-169.

20. Khalkhali HR, Foroutan M, Khademvatan S, Majidiani H, Aryamand S, Khezri P, Aminpour A. Prevalence of cystic echinococcosis in Iran: a systematic review and meta-analysis. *J Helminthol* 2017; **92**: 260-268.

21. Barendregt J J, Doi S A, Lee YY, Norman RE, Vos T. A meta-analysis of prevalence: Theory and methods. *J Epidemiol Commun Health* 2013; **67**: 974-978.

22. Higgins J, Green S. Cochrane Handbook for Systematic Reviews of Interventions. Cochrane Book Series. Published by John Wiley & Sons, Ltd., UK; 2008

23. Tufanaru C, Munn Z, Stephenson M,Aromataris E. Fixed or random-effects meta-analysis? Common methodological issues in systematic reviews of effectiveness. *Int J Evid Based Healthc* 2015; **13**:196–207.

24. Borenstein M, Hedges LV, Higgins JPT, Rothstein HR. Introduction to Meta-Analysis. 1st edition. John Wiley & Sons, Ltd., UK; 2009

25. Biyansa A. Sero-epidemiology of toxoplasmosis among antenatal care attending pregnant women in selected hospitals in Central and South Gondar Zones, Ethiopia. MSc thesis, UoG, Gondar, Ethiopia; 2019

26. Teweldemedhin M, Gebremichael A, Geberkirstos G, Hadush H, Gebrewahid T, Asgedom SW, et al. Seroprevalence and risk factors of *Toxoplasma gondii* among pregnant women in Adwa district, northern Ethiopia. *BMC Infect Dis* 2019; **19**.

27. Jula J, Girones G, Edao B, Deme C, Cebrian J, Butrón L, et al. Seroprevalence of *Toxoplasma gondii* infection in pregnant women attending antenatal care in southern Ethiopia. *Rev Esp Quimioter* 2018; **31**: 363–366.

28. Ahmed M, Tilahun G, Berhe N, Yigeremu M, Fekade D. Seroprevalence of IgG and IgM anti-*Toxoplasma gondii* antibodies among pregnant women attending antenatal clinic at Tikur Anbessa specialized hospital, Addis Ababa, Ethiopia. Int *J Current Med Pharm Res* 2017; **3**(1): 1262-1269.

29. Negero J, Yohannes M, Woldemichael K, Tegegne D. Seroprevalence and potential risk factors of T. gondii infection in pregnant women attending antenatal care at Bonga Hospital, Southwestern Ethiopia. *Int J Infect Dis* 2017; **57**: 44–49.

30. Negussie A, Beyene E, Palani S. Toxoplasmosis and Associated Risk Factors in Antenatal Clinic follow up Pregnant Women in Selected Health Institutes of Jigjiga, East Ethiopia. *Int J Trop Dis Health* 2017; **21**: 1–7.

31. Yohanes T, Zerdo Z, Chufamo N, Abossie A. Seroprevalence and Associated Factors of *Toxoplasma gondii* Infection among Pregnant Women Attending in Antenatal Clinic of Arba Minch Hospital, South Ethiopia: Cross-Sectional Study. *Transl Biomed* 2017; **8**: 1–7.

32. Abamecha, F., Awel, H. Seroprevalence and risk factors of *Toxoplasma gondii* infection in pregnant women following antenatal care at Mizan Aman General Hospital, Bench Maji Zone (BMZ), Ethiopia. *BMC Infect Dis* 2016; **16**.

33. Agmas B, Tesfaye R, Koye DN. Seroprevalence of Toxoplasma gondii infection and associated risk factors among pregnant women in Debre Tabor, Northwest Ethiopia. *BMC Res Notes* 2015;**8**.

34. Gelaye W, Kebede T, Hailu A. High prevalence of anti-toxoplasma antibodies and the absence of *Toxoplasma gondii* infection risk factors among pregnant women attending routine antenatal care in two hospitals of Addis Ababa, Ethiopia. *Int J Infect Dis* 2015; **34**: 41–45.

35. Hailu AH, Negashe KN, Tasew AT, Getachew MG, Sisay TS, Jibat TJ, et al. Sero–Prevalence, and Associated Risk Factors of *Toxoplasma gondii* Infection in Pregnant Women and HIV/AIDS Patients in Selected Cities of Ethiopia. *Banat's J Biotechnol* 2014;**10**: 17-29.

36. Endris M, BelyhunY, Moges F, Adefiris M, Tekeste Z, Mulu A, Kassu A. Seroprevalence and associated risk factors of *Toxoplasma gondii* in pregnant women attending in Northwest Ethiopia. *Iran J Parasitol* 2014; **9**: 407–414.

37. Gebremedhin EZ, Abebe AH, Tessema TS, Tullu KD, Medhin G, Vitale M, et al. Seroepidemiology of *Toxoplasma gondii* infection in women of child-bearing age in central Ethiopia. *BMC Infect Dis* 2013;**13**.

38. Zemene E, Yewhalaw D, Abera S, Belay T, Samuel A, Zeynudin A. Seroprevalence of *Toxoplasma gondii* and associated risk factors among pregnant women in Jimma town, Southwestern Ethiopia. *BMC Infect Dis* 2012;**12**.

39. Fewzia M. Sero-burden of *Toxoplasma gondii* and associated risk factors among HIV/AIDS patients in Armed Forces Referal and Teaaching Hospital, Addis Ababa, Ethiopia. MSc thesis, Addis Ababa University, Addis Ababa, Ethiopia; 2016

40. Zeleke AJ, Melsew YA. Seroprevalence of *Toxoplasma gondii* and associated risk factors among HIV-infected women within the reproductive age group at Mizan Aman General Hospital, Southwest Ethiopia: A cross-sectional study. *BMC Res Notes* 2017; **10**.

41. Tegegne D, Abdurahaman M, Mosissa T, Yohannes M. Anti-*Toxoplasma* antibodies prevalence and associated risk factors among HIV patients. *Asian Pac J Trop Med* 2016; **9**: 460–464.

42. Yesuf M K, Melese Z T. Prevalence of Toxoplasmosis in HIV/AIDS Patients in Mettu Karl Hospital. *Am J Health Res* 2015; **3**(3): 183-188.

43. Yohanes T, Debalke S, Zemene E. Latent *Toxoplasma gondii* infection and associated risk factors among HIV-infected individuals at Arba Minch Hospital, south Ethiopia. *AIDS Res Treat* 2014; **2014**.

44. Walle F, Kebede N, Tsegaye A, Kassa T. Seroprevalence and risk factors for toxoplasmosis in HIV infected and non-infected individuals in Bahir Dar, Northwest Ethiopia. *Parasit Vect* 2013; **6**.

45. Muluye D, Wondimeneh Y, Belyhun Y, Moges F, Endris M, Ferede G, et al. Prevalence of *Toxoplasma gondii* and Associated Risk Factors among People Living with HIV at Gondar University Hospital, Northwest Ethiopia. *ISRN Trop Med* 2013; **2013**: 1–5.

46. Aleme H, Tilahun G, Fekada D, Berhe N, Medhin G. Seroprevalence of Immunoglobulin-G and of Immunoglobulin-M Anti- *Toxoplasma gondii* antibodies in Human Immunodeficiency Virus Infection / Acquired Immunodeficiency Syndrome Patients at Tikur Anbessa. *J Infect Dis Ther* 2013; **1:** 1–5.

47. Shimelis T, Tebeje M, Tadesse E, Tegbaru B, Terefe A. Sero-prevalence of latent *Toxoplasma gondii* infection among HIV-infected and HIV-uninfected people in Addis Ababa, Ethiopia: A comparative cross-sectional study. *BMC Res Notes* 2009; **2**.

48. Woldemichael T, Fontanet AL, Sahlu T, Gilis H, Messele T, De Rinke Wit, TF et al. Evaluation of the Eiken latex agglutination test for anti-*Toxoplasma* antibodies and seroprevalence of *Toxoplasma* infection among factory workers in Addis Ababa, Ethiopia. *Trans R Soc Trop Med Hyg* 1998; **92**: 401–403.

49. Achaw B, Tesfa H, Zeleke AJ, Worku L, Addisu A, Yigzaw N, et al. Seroprevalence of *Toxoplasma gondii* and associated risk factors among psychiatric outpatients attending the University of Gondar Hospital, Northwest Ethiopia. *BMC Infect Dis* 2019; **19**.

50. Guebre-Xabier M, Nurilign A, Gebre-Hiwot A, Hailu A, Sissay Y, Getachew E, et al. Sero-epidemiological survey of *Toxoplasma gondii* infection in Ethiopia. *Ethiop Med J* 1993; **31**: 201–208.

51. Negash T, Tilahun G, Medhin G. Seroprevalence of *Toxoplasma gondii* in Nazaret town, Ethiopia. *East Afr J Public Health* 2008; **5**: 211–214.

52. Fan CK, Hung CC, Su KE, Chiou HY, Gil V, Ferreira MDR, Tseng LF. Seroprevalence of *Toxoplasma gondii* infection among inhabitants in the Democratic Republic of Sao Tome and Principe. *Tran R Soc Trop Med Hyg* 2007; **101**(11): 1157-1158.

53. Ayi I, Edu S, Apea-Kubi KA, Boamah D, Bosompem KM, Edoh D. Sero-epidemiology of toxoplasmosis amongst pregnant women in the greater Accra region of Ghana. *Ghana Med J* 2009; **43**(3): 107-114.

54. Abu EK, Boampong JN, Ayi I, Ghartey-Kwansah G, Afoakwah R, Nsiah P, Blay E. Infection risk factors associated with seropositivity for Toxoplasma gondii in a population-based study in the Central Region, Ghana. Epidemiol Infect 2014; 1-9.

55. Doudou Y, Renaud P, Coralie L, Jacqueline F, Hypolite S, Hypolite M, et al. Toxoplasmosis among pregnant women: high seroprevalence and risk factors in Kinshasa, Democratic Republic of Congo. *Asian Pac J Trop **B**iomed* 2014; **4**(1): 69–74.

56. Njunda AL, Assob JCN, Nsagha DS, Kamga HL, Nde PF, Yugah VC. Seroprevalence of *Toxoplasma gondii* infection among pregnant women in Cameroon. *J Public Health Africa* 2011; **2**: 98–101.

57. Barbosa IR, de Carvalho Xavier Holanda CM, de Andrade-Neto VF. Toxoplasmosis screening and risk factors amongst pregnant females in Natal, northeastern Brazil. *Trans R Soc Trop Med Hyg* 2009;**103**: 377-82.

58. Da Silva MG, Vinaud MC, De Castro AM. Prevalence of toxoplasmosis in pregnant women and vertical transmission of *Toxoplasma gondii* in patients from basic units of health from Gurupi, Tocantins, Brazil, from 2012 to 2014. *PLoS One* 2015;**10**: 1–15.

59. Mwambe B, Mshana SE, Kidenya BR, Massinde AN, Mazigo HD, Michael D, et al. Sero-prevalence and factors associated with *Toxoplasma gondii* infection among pregnant women attending antenatal care in Mwanza, Tanzania. *Parasit Vect* 2013; **6**: 2–6.

60. Murebwayire E, Njanaake K, Ngabonziza JCS, Njunwa KJ, Jaoko W. Seroprevalence and risk factors of *Toxoplasma gondii* infection among pregnant women attending antenatal care in Kigali, Rwanda. *Tanzan J Health Res* 2017; **19**: 2–9.

61. Mohammed MW, Abdalla HS, Satti AB, Mustafa TB, Kabbashi AS. Sero-prevalence and associated risk factors of *Toxoplasma gondii* infection among pregnant women attending Omdurman friendship hospital, Omdurman. *Ment Health Addict Res* 2018; **3**(1): 1–3.

62. Mustafa M, Fathy F, Mirghani A, Mohamed MA, Muneer MS, Ahmed AE, et al. Prevalence and risk factors profile of seropositive *Toxoplasmosis gondii* infection among apparently immunocompetent Sudanese women. *BMC Res Notes* 2019; **12**: 1–6.

63. Kistiah K, Winiecka-Krusnell J, Barragan A, Karstaedt A, Frean J. Seroprevalence of *Toxoplasma gondii* Infection in HIV-positive and HIV-negative subjects in Gauteng, South Africa. *S Afr J Epidemiol Infect* 2011; **26**: 225–228.

64. Domingos A, Ito LS, Coelho E, Lúcio JM, Matida LH, Ramos AN. Seroprevalence of *Toxoplasma gondii* igg antibody in HIV/AIDS-infected individuals in Maputo, Mozambique. *Rev Saude Publica* 2013; **47**: 890–896.

65. Ogoina D, Onyemelukwe GC, Musa BO, Obiako RO. Seroprevalence of IgM and IgG antibodies to *Toxoplasma* infection in healthy and HIV-positive adults from Northern Nigeria. *J Infect Dev Ctries* 2013; **7**(5): 398-403.

66. Okwuzu JO, OdunukweNN, Ezechi OC, Gbajabiamila TA, Musa AZ, Ezeobi PM, et al. *Toxoplasma gondii* infection in HIV/AIDS: Prevalence and Risk Factors. *Afr J Clin Exper Microb* 2014;**15**: 97.

67. Bamba S, Cissé M, Sangaré I, Zida A, Ouattara S, Guiguemdé RT. Seroprevalence and risk factors of *Toxoplasma gondii* infection in pregnant women from Bobo Dioulasso, Burkina Faso. *BMC Infect Dis* 2017; **17**: 1-6

68. Frimpong C, Makasa M, Sitali L, Michelo C. Seroprevalence and determinants of toxoplasmosis in pregnant women attending antenatal clinic at the university teaching hospital, Lusaka, Zambia. *BMC Infect Dis* 2017; **17**(10): 1–8.

69. Opintan JA, Awadzi BK, Biney IJK, Ganu V, Doe R, Kenu E, et al. High rates of cerebral toxoplasmosis in HIV patients presenting with meningitis in Accra, Ghana. *Trans R Soc Trop Med Hyg* 2017; **111**(10): 464–471.

70. Todjom FG, Tsapi EM, Gamago GA, Vignoles P, Pone JW, Teukeng FD. Seroprevalence of toxoplasmosis and associated risk factors in pregnant women at the Protestant Hospital, Mbouo-Bandjoun, Cameroon. *Afr J Clin Exper Microbiol* 2019; **20**(3): 221-230.

71. Petersson K, Stray-Pedersen B, Malm G, Forsgren M, Evengård B . Seroprevalence of *Toxoplasma gondii* among pregnant women in Sweden. *Acta Obstetricia Gynecol Scandin* 2000; **79**(10): 824-829.

72. Jones JL, Kruszon-Moran D, Wilson M, McQuillan G, Navin T, McAuley JB. *Toxoplasma gondii* infection in the United States: seroprevalence and risk factors. *Am J Epidemiol* 2001; **154**(4): 357-365.

73. Alvarado-Esquivel C, Sifuentes-Álvarez A, Narro-Duarte SG, Estrada-Martínez S, Díaz-García JH, Liesenfeld O, et al. Seroepidemiology of *Toxoplasma gondii* infection in pregnant women in a public hospital in northern Mexico. *BMC Infect Dis* 2006; **6**: 1–7.

74. Nowakowska D, Stray-Pedersen B, Śpiewak E, Sobala W, Małafiej E, Wilczyński J. Prevalence and estimated incidence of *Toxoplasma* infection among pregnant women in Poland: A decreasing trend in the younger population. *Clin Microbiol Infect* 2006; **12**: 913–917.

75. De Paschale M, Agrappi C, Clerici P, Mirri P, Manco MT, Cavallari S, et al. Seroprevalence and incidence of *Toxoplasma gondii* infection in the Legnano area of Italy. *Clin Microbiol Infect* 2008; **14**: 186–189.

76. Ramsewak S, Gooding R, Ganta K, Seepersadsingh N, Adesiyun AA. Seroprevalence and risk factors of *Toxoplasma gondii* infection among pregnant women in Trinidad and Tobago. *Rev Panam Salud Publica* 2008; **23**(3): 164–70.

77. Liu Q, Wei F, Gao S, Jiang L, Lian H, Yuan B, et al. *Toxoplasma gondii* infection in pregnant women in China. *Trans R Soc Trop Med Hyg* 2009; **103**: 162–166.

78. Flatt A, Shetty N. Seroprevalence and risk factors for toxoplasmosis among antenatal women in London: A re-examination of risk in an ethnically diverse population. *Eur J Public Health* 2012; **23**: 648–652.

79. Almushait MA, Dajem SMB, Elsherbiny NM, Eskandar MA, Al Azraqi TA, Makhlouf LM. Seroprevalence and risk factors of *Toxoplasma gondii* infection among pregnant women in southwestern, Saudi Arabia. *J Parasit Dis* 2014; **38**: 4–10.

80. Chemoh W, Sawangjaroen N, Siripaitoon P, Andiappan H, Hortiwakul T, Sermwittayawong N, et al. *Toxoplasma gondii* - Prevalence and risk factors in HIV-infected patients from Songklanagarind Hospital, Southern Thailand. *Front Microbiol* 2015; **6**: 1–5.

81. Iddawela D, Vithana SMP, Ratnayake C. Seroprevalence of toxoplasmosis and risk factors of Toxoplasma gondii infection among pregnant women in Sri Lanka: A cross-sectional study. *BMC Public Health* 2017;**17**: 1–6.

82. Arefkha N, Sarkari B, Afrashteh M, Rezaei Z, DehghaniM. *Toxoplasma gondii*: the prevalence and risk factors in HIV-infected patients in Fars province, southern Iran. *Iran Red Crescent Med J* 2018; **20**(6): e66521.

83. Daryani A, Sarvi S, Aarabi M, Mizani A, Ahmadpour E, Shokri A, et al. Seroprevalence of *Toxoplasma gondii* in the Iranian general population: a systematic review and meta-analysis. *Acta Trop* 2014;**137**: 185-194.

84. Alsammani MA. Sero-epidemiology and risk factors for *Toxoplasma gondii* among pregnant women in Arab and African countries. *J Parasit Dis* 2016; **40**: 569-579.

85. Minbaeva G, Schweiger A, Bodosheva A, Kuttubaev O, Hehl AB, Tanner I, et al. *Toxoplasma gondii* infection in Kyrgyzstan: seroprevalence, risk factor analysis, and the estimate of congenital and AIDS-related toxoplasmosis. *PLoS Negl Trop Dis* 2013; **7**: e2043.

86. Abbas, IE, Villena I, Dubey, JP. A review on toxoplasmosis in humans and animals from Egypt. *Parasitol* 2019; 1-25.

87. Al-Eryani SMA, Al-Mekhlafi AM, Al-Shibani LA, Mahdy MMK, Azazy AA. *Toxoplasma gondii* infection among pregnant women in Yemen: Factors associated with high seroprevalence. *J Infect Dev Ctries* 2016; **10**: 667–672.

88. Tagwireyi WM, Etter E, Neves L. Seroprevalence and associated risk factors of *Toxoplasma gondii* infection in domestic animals in southeastern South Africa. *Onderstepoort J Vet Res* 2019; **86**: 1–6.

89. Alvarado-Esquivel C, Estrada-Martínez S, Pizarro-Villalobos H, Arce-Quiñones M, Liesenfeld O, Dubey JP. Seroepidemiology of *Toxoplasma gondii* infection in general population in a northern Mexican city. *J Parasitol* 2011; **97**(1): 40-44.

90. Gao XJ, Zhao ZJ, He ZH, Wang T, Yang TB, Chen XG, et al. *Toxoplasma gondii* infection in pregnant women in China. *Parasitol* 2012; **139**: 139–147.

91. Rosso F, Les JT, Agudelo A, Villalobos C, Chaves JA, Tunubala GA, et al. Prevalence of infection with *Toxoplasma gondii* among pregnant women in Cali, Colombia, South America. *Am J Trop Med Hyg* 2008; **78**(3): 504-508.

92. Rouatbi M, Amairia S, Amdouni Y, Boussaadoun MA, Ayadi O, Al-Hosary AAT. *Toxoplasma gondii* infection and toxoplasmosis in North Africa: A review. *Parasit* 2019: **26**.

93. Falusi O, French AL, Seaberg EC, Tien PC, Watts DH, Minkoff H, Cohen MH. Prevalence and predictors of *Toxoplasma* seropositivity in women with and at risk for human immunodeficiency virus infection. *Clin Infect Dis* 2002; **35**(11): 1414-1417.

94. Kamal AM, Ahmed AK, Abdellatif MZM, Tawfik M, Hassan EE. Seropositivity of toxoplasmosis in pregnant women by ELISA at Minia university hospital, Egypt. *Korean J Parasitol* 2015; **53**: 605–610.

95. Sun X F, Liu PZ, Song YN, Zhang SJ, Ren JQ. Study on *Toxoplasma* infection among some pregnant women in Qingdao in 2005. *Prev Med Tribune* 2006;**12**: 675-676.

96. Al-Qurashi AR, Ghandour AM, Obied OE, Al-Mulhim AA, Makki SM. Seroepidemiological study of *Toxoplasma gondii* infection in the human population in the Eastern Region. *Saudi Med J* 2001; **22**: 13–18.

97. Al-Qurashi AR. Seroepidemiological study of toxoplasmosis in rural areas in the eastern region of Saudi Arabia. *J Egypt Soc Parasitol* 2004; **34**(1): 23-34.

98. Rufener S, Mäusezahl D, Mosler H, Weingartner R. Quality of drinking-water at source and point of the consumption-drinking cup as a high potential recontamination risk: A field study in Bolivia. *J Health Popul Nutr* 2010; **28**(1): 34-41.

99. Bouzid M, Kintz E, Hunter PR. Risk factors for *Cryptosporidium* infection in low and middle-income countries: A systemic review and meta-analysis. *PLOS Negl Trop Dis* 2018; **12**(6) e0006553.

100. Pappas G, Roussos N, Falagas ME. Toxoplasmosis snapshots: global status of *Toxoplasma gondii* seroprevalence and implications for pregnancy and congenital toxoplasmosis. *Int J Parasitol* 2009; **39**: 1385–1394.

101. Jones JL, Dargelas V, Roberts J, Press C, Remington JS, Montoya JG. Risk factors for *Toxoplasma gondii* infection in the United States. *Clin Infect Dis* 2009; **49**: 878-884.

102. Lopes FMR, Mitsuka-Breganó R, Gonçalves DD, Freire RL, Karigyo CJT, Wedy GF, et al. Factors associated with seropositivity for anti-*Toxoplasma* gondii antibodies in pregnant women of Londrina, Paraná, Brazil. *Memórias do Instituto Oswaldo Cruz* 2009; **104**(2): 378-382.

Click here to access/download;Figure;Figure1.doc

PRISMA Flow Diagram used for Study Searching process

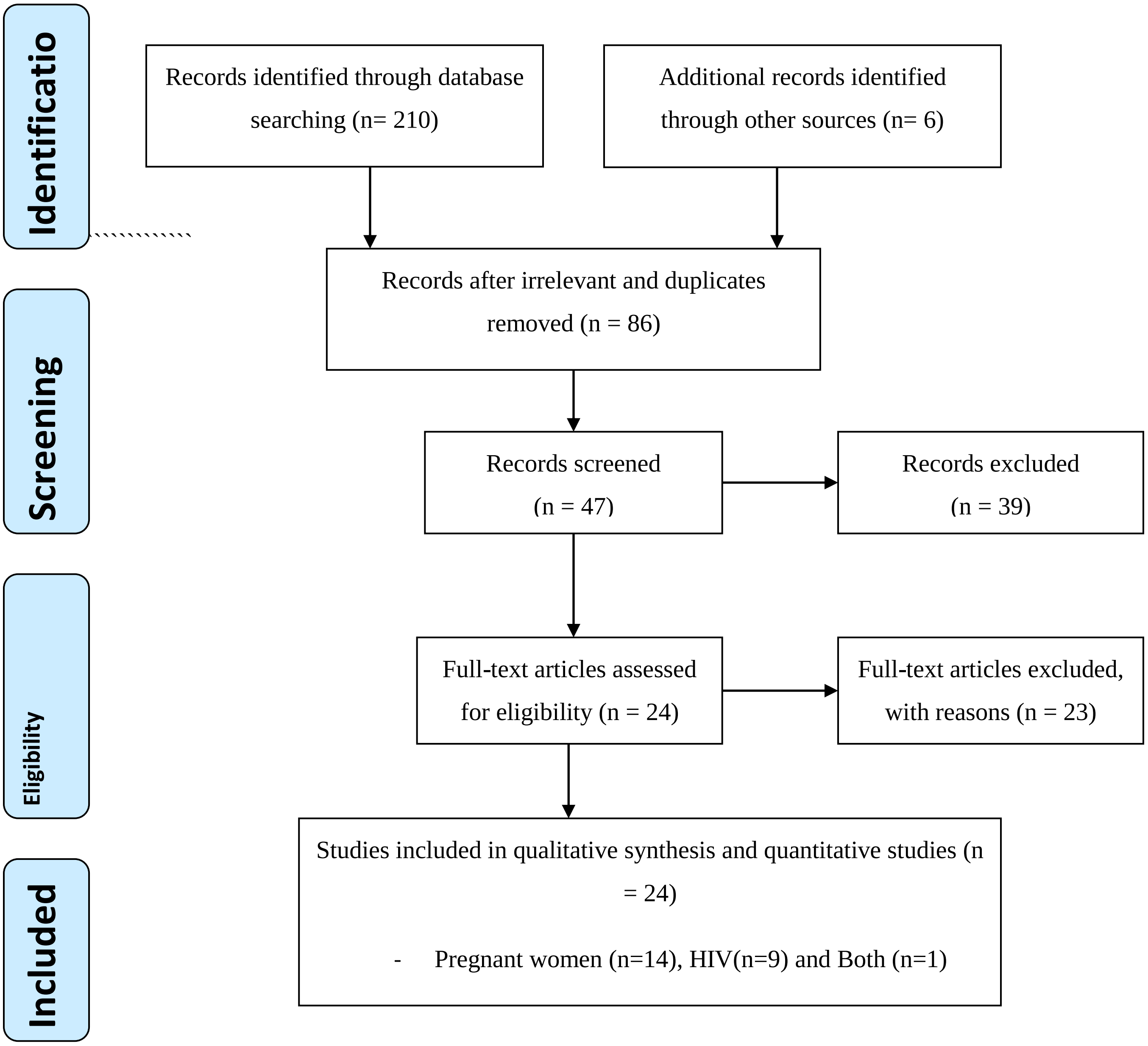

Figure 1: PRISMA Flow Diagram used for Study Searching process: adapted from Moher *et al.* (2010)

Click here to access/download;Figure;Figure2.doc

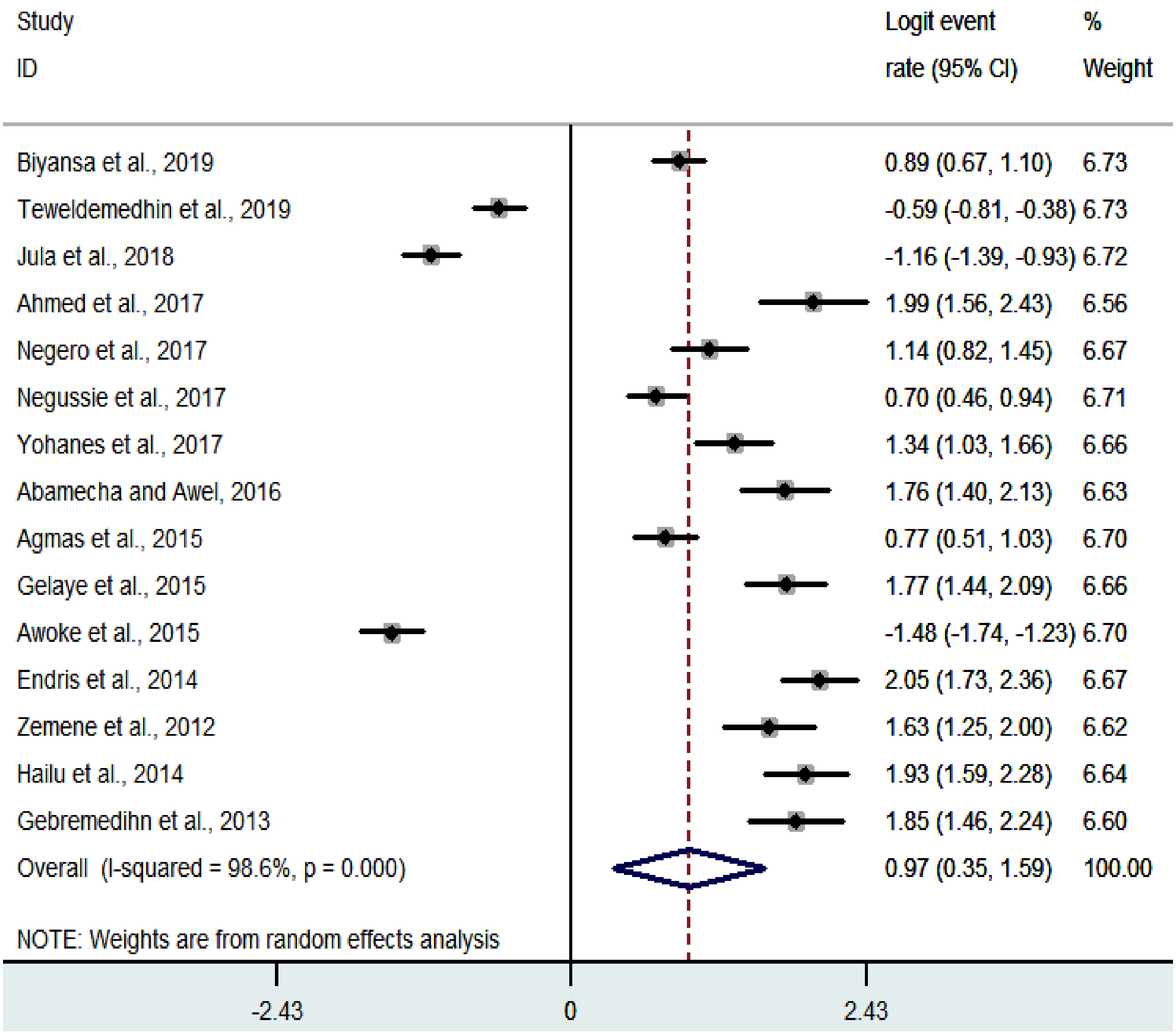

Figure 1: Forest plot of the logit event rate estimate (lp) in pregnant women

Click here to access/download;Figure;Figure5.doc

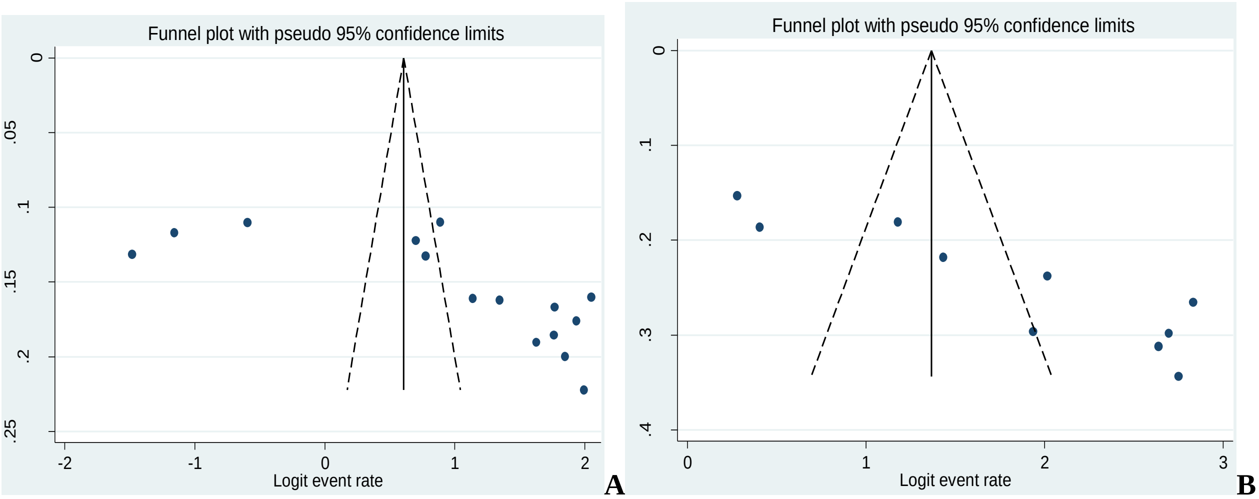

Figure 1: Funnel plots of the logit event rate of toxoplasmosis in pregnant women (A) and HIV infected (B)

Click here to access/download;Figure;Figure3.doc

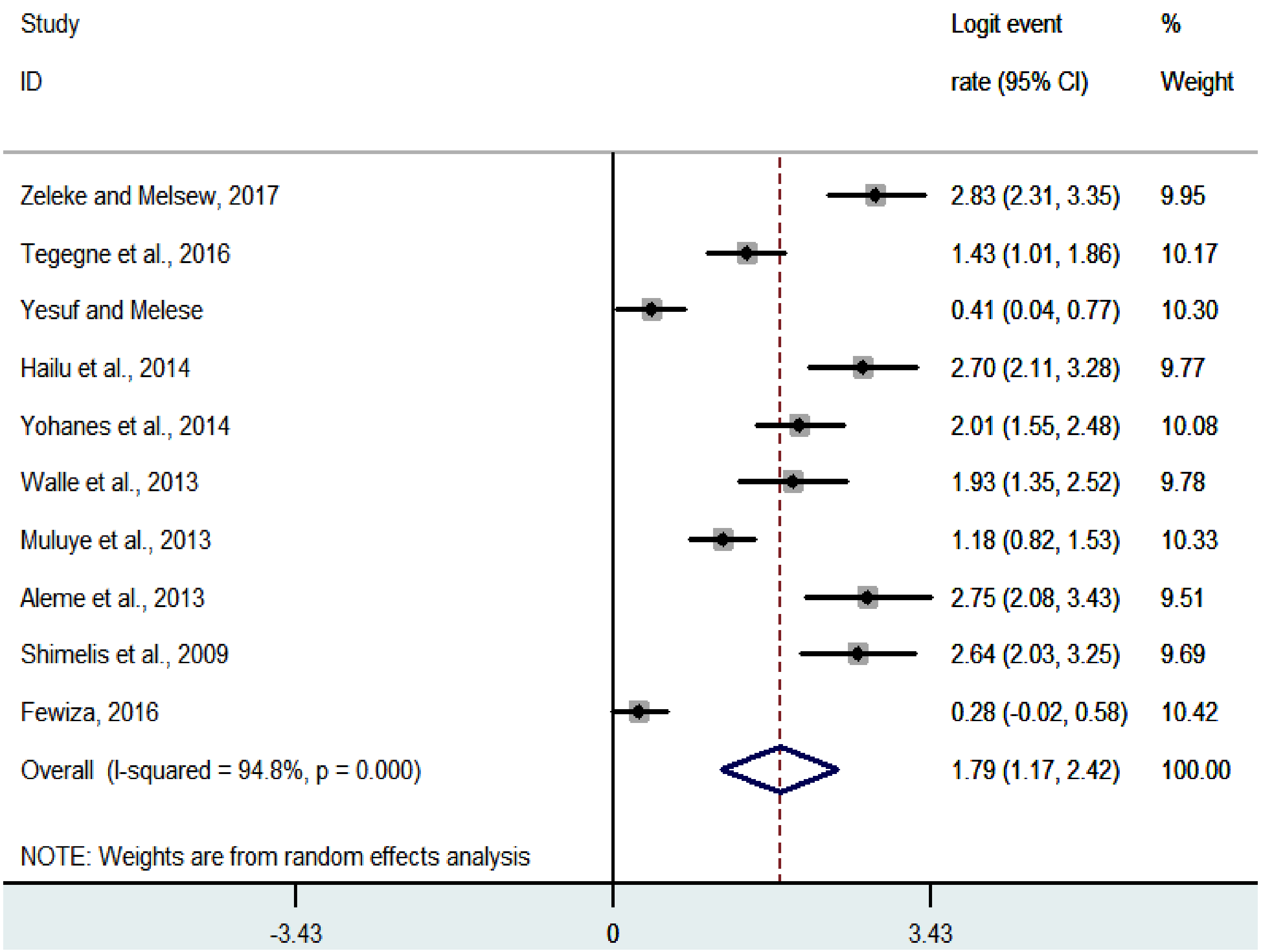

Figure 1: Forest plot of the logit event rate estimate (lp) in HIV infected individuals

Click here to access/download;Figure;Figure4.doc

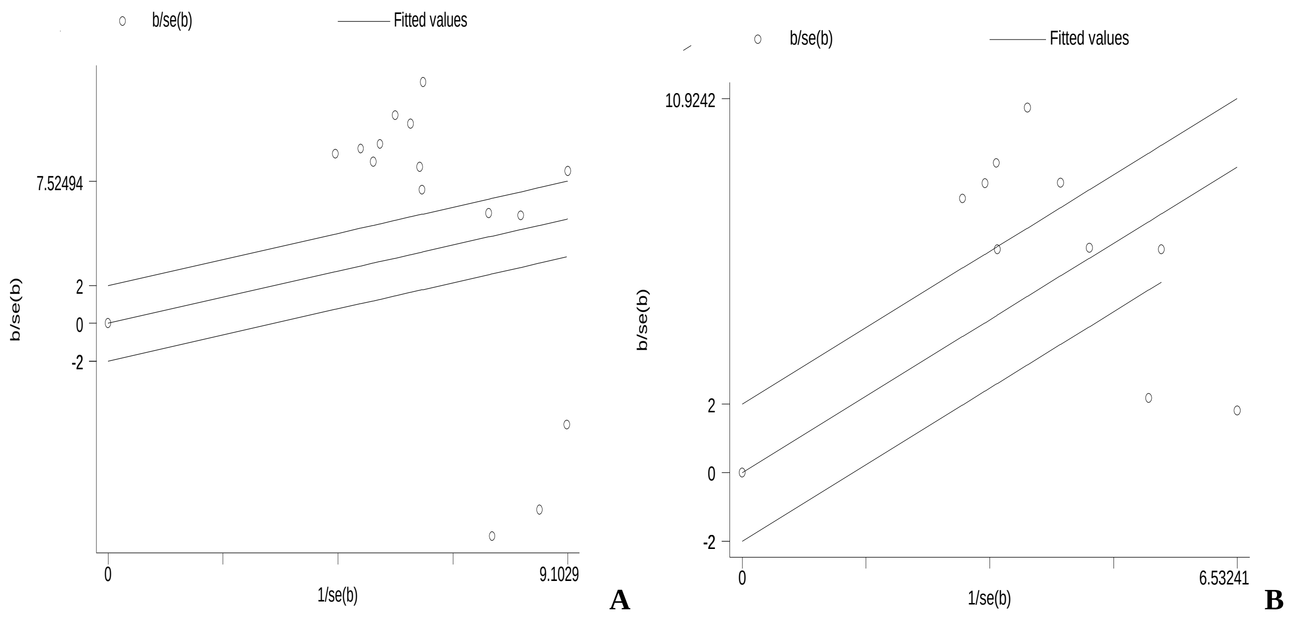

Figure 1: Galbraith plots for seroprevalence of toxoplasmosis in pregnant women (A) & HIV infected (B)

Click here to access/download;Figure;Figure6.doc

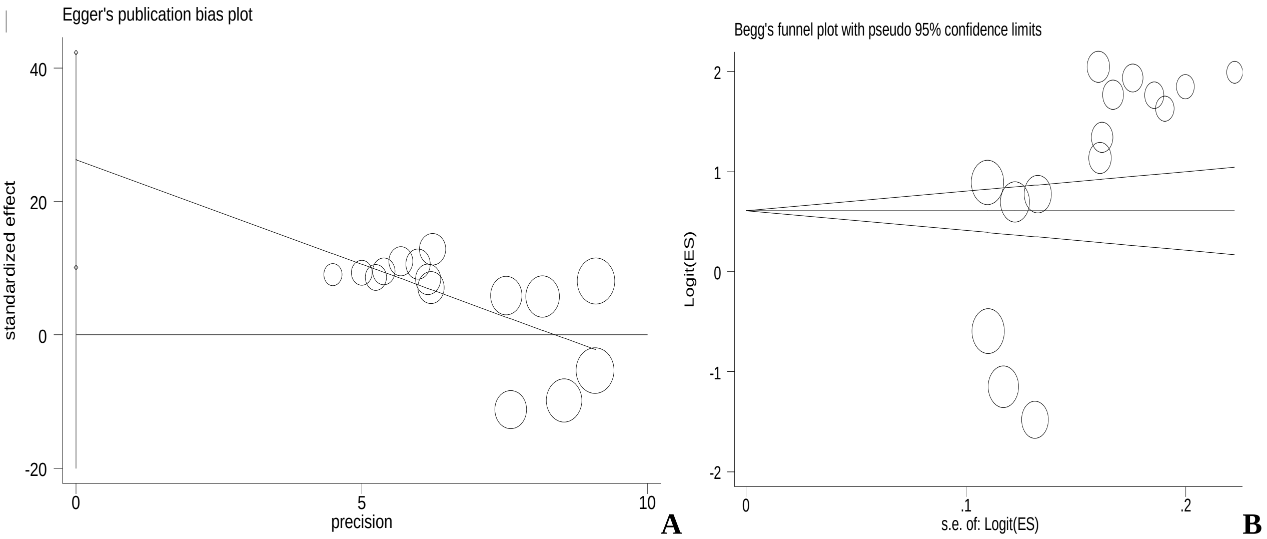

Figure 1: Eggers's publication bias plot (**A**) and Begg's funnel plot (**B**) reports for pregnant women studies

Click here to access/download;Figure;Figure7.doc

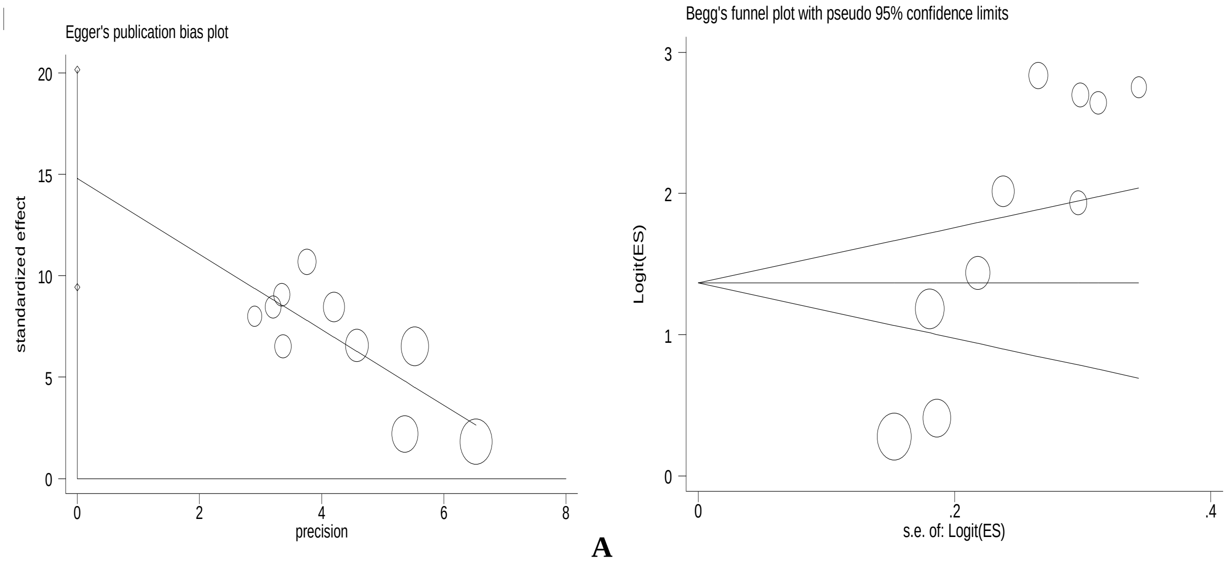

A **B**

Figure 1: Eggers's publication bias plot (**A**) and Begg's funnel plot (**B**) reports for HIV infected individual studies

Click here to access/download
Supporting Information - Compressed/ZIP File Archive
Sup1 File.doc

Click here to access/download
Supporting Information - Compressed/ZIP File Archive
Sup2 File.doc

Click here to access/download

Supporting Information - Compressed/ZIP File Archive

Sup3 File.docx

Click here to access/download
Supporting Information - Compressed/ZIP File Archive
Sup4 File.docx

Author summary

Toxoplasmosis is a zoonotic protozoan infection caused by *Toxoplasma gondii*. *Toxoplasma gondii* causes a considerable public health impact with higher burden in developing countries. Estimating pooled seroprevalence of *Toxoplasma* infection in high risk groups can draw health policy makers' attention in planning a screening program and control needs. Several risk factors determine the circulation of *Toxoplasma gondii* between intermediate and final hosts. Health information has high implication to control *Toxoplasma* infection in high risk groups of communities. Sociocultural and economic factors contribute high burden of toxoplasmosis in developing countries. In the present study, we tried to identify the potential factors influencing the seroprevalence of *Toxoplasma* infection pregnant women and HIV infected individuals of Ethiopia.
