# Supplementary material for: Potential risk factors associated with seropositivity for Toxoplasma gondii among pregnant women and HIV infected individuals in Ethiopia: A systematic review and meta-analysis
Source: PLoS Negl Trop Dis. 2020 Dec 15;14(12):e0008944. doi: 10.1371/journal.pntd.0008944 (PMC7771857; doi:10.1371/journal.pntd.0008944)
Supplement: Attachment — Submitted filename: PLOS review.docx [file pntd.0008944.s007.docx]

| **Date:** | July 31 2020 |
| --- | --- |
| **To:** | "PLOS Neglected Tropical Diseases" plosntds@plos.org |
| **From:** | Zewdu Seyoum Tarekegn; [zewdusagera@gmail.com](mailto:zewdusagera@gmail.com) or [Zewdu.seyoum@uog.edu.et](mailto:Zewdu.seyoum@uog.edu.et) |
| **Subject:** | **Responses for reviewers and editors for manuscript: PNTD-D-20-00491** |

**Dear reviewers and editors**

First of all, we thank you very much for your concrete comments and suggestion to strengthen this manuscript. Having this, we tried to consider all comments and give correction as of the reviewer and editors suggestion.

**1. Reviewer #1:**

This is a work of high importance and it is generally well conducted. I have a couple of minor but essential comments that should be addressed. They can be found as comments in the attached document.

- **Accepted, corrected and included corrections in the text and tables**

Reviewer #3: The main problem (in my opinion) for this study is novelty of results, as we know there are some recent global and regional meta-analyses considering prevalence and risk factors of T. gondii in pregnant women and HIV patients; and data from Ethiopia is present in them.

- Introduction
  * There are four global studies with regard to acute and latent toxoplasmosis in pregnant women (https://doi.org/10.1371/journal.pntd.0007807 and https://doi.org/10.1016/j.cmi.2020.01.008) and in HIV patients (10.1097/QAD.0000000000002424 and http://dx.doi.org/10.1016/S2352-3018 (17)30005-X). I think use of results of these studies is informative for introduction. Moreover in discussion results of present study should be compared with global and regional prevalences according to results from abovementioned studies.
- **Accepted and considered for this review**
- Methods
- Data extraction: please mention details for risk factors.
- **Accepted and corrected**
- Meta-analysis:
- The studies by Foroutan-Rad et al. and Khalkhali et al. could not be a protocol. Please refer to original study by DerSimonian and Laird (1986).
- **Accepted and corrected**
- In the following sentence, used reference is invalid and authors should add valid reference. "Cochran's Q-statistics and inverse variance index (I2) were computed to determine the heterogeneity and inconsistency among studies, respectively [2]."
- **Accepted and corrected**
- I think only random effects model is proper for estimation of pooled prevalence rates.
- **We thank the reviewer very much for his/her concrete comments. We accepted and considered the reviewer comments. Actually, the reviewer suggestion is exact but we put our criteria following previous suggestion when we can use the random-effect model.**
- Results
- Meta-analysis and bias assessment: please add 95% CI related with pooled prevalences.
- **Accepted and corrected**
- Results for prevalence rates presented in text is inconsistent with forest plots (figure 2 and 3).
- **We thank the reviewer for his/her comments. However, the result depicts with forest plots with logit event rate rather than prevalence. We calculated the pooled prevalence using the formula: p= exp (lp)/ (exp (lp) +1) given by Barendregt et al. (2013), where exp (lp) = exponential of the logit event estimate. Using this formula if you substitute logit event rate (lp) by 0.97, where event rate or prevalence (p): exp(0.97)/(exp(0.97)+1)= 0.725 ¬ 0.725*100% = 72.5%. So, please consider the result in the text with prevalence rather logit event rate in the forest plot. Actually, this is our fault to show how we can reach with this result.**
- All fig are numbered as figure 1. Please correct them.
- **Accepted and corrected**
- There are several editorial errors that should be modified in revise.
- **Accepted and corrected**

**2. Deputy Editor:**

**Comments from Deputy Editor:**

- The author summary should be a summary of this work. I recommend to largely rewrite the current summary.
- **Accepted and corrected.**
- The conclusions and recommendations given must be based on this study.
- **Accepted and corrected.**
- What is meant by ‘burden’ in the first concluding sentence?
- **To mean that the risk of infection is very high**
- Can it be concluded directly from this study that routine serological screening and health promotion should be started?
- **Based on the information available, since *Toxoplasma gondii* is a neglected zoonotic disease, there is no routine screening in pregnant women and HIV infected individuals in Ethiopia. But, our finding shows that the prevalence of *T. gondii* infection in pregnant women and HIV infected individual is very high. Thus, it can be expected that the pathogen may induce considerable health impact during pregnancy resulting in congenital toxoplasmosis and cerebral toxoplasmosis in HIV infected individuals. Hence, routine serological screening during pregnancy may help to reduce congenital toxoplasmosis. Similarly, serological screening may help us to reduce the impact of cerebral toxoplasmosis.**
- Can the aspects about cats mentioned in the last paragraph be concluded from this study?
- **Accepted and corrected.**
- This study was limited to one country. Have similar studies been made in other countries? I expect in most countries, the number of studies conducted is too low for a similar study – this could be discussed.
- **Accepted and corrected.**
- Relevant basic background information about the setting of the study (e.g. population of Ethiopia) should be given. It would be good to emphasize why this work was important to do, in particular the importance to international audience. It is important that the setting is repeated in the Discussion, to make it clear the results are from studies performed in one country. E.g. the first sentence of the last paragraph should specify the setting.
- **Accepted and corrected**
- Limitations of the study need to be discussed more.
- **Accepted and corrected**
- Please check the use of the key expressions everywhere in the manuscript very carefully. In particular, ‘toxoplasmosis’ is usually used for the clinical disease; for the subclinical infection detected by serology, ‘T. gondii infection’ is better expression. For example, please change ‘Seroprevalence of toxoplasmoses-> T. gondii seroprevalence; and please rephrase ‘95% confidence intervals (Cis) of toxoplasmoses. The name of the parasite should be written in full (Toxoplasma gondii, in Italics).
- **Accepted and corrected.**
- Please check that abbreviations are explained when used for the first time (PRF).
- **It has been explained in background part**
- Avoid using the word ‘**only**’ in results.
- **Accepted and corrected**
- Table 1: Sampling method ‘simple’ should be explained.
- **Accepted and corrected. It was to say: Simple random sampling**
- The variable ‘age’ was dichotomized; this needs to be mentioned in Methods.
- **Accepted and corrected.**
- Consider changing ‘Ethiopian’ to ‘in Ethiopia’, if the inclusion criteria of the original studies did not include the nationality of the individuals.
- **Accepted and corrected.**
- In the Discussion, the sentence starting ‘**Higher Toxoplasma seroprevalence was reported**’ is confusing, as two of the three estimates mentioned are not higher than the pooled estimates obtained.
- **Accepted and corrected**
- Ref nr 48 was not included in the study, but it is used for comparison. Please check/clarify.
- **We thank you very much this is our fault the study was conducted on factory workers rather than HIV infected individuals. Thus, we corrected it.**
- In the Discussion, the sentence starting ‘Comparable findings…’ puts the study in the global setting, but, the references used (nr 52-58) do not cover different areas across the world very well. In some parts of the world, the seroprevalence is clearly lower. I can see an opportunity to widen the interest of this work by including a wider scope here.
- **Accepted and corrected.**
- ‘infected meat’ -> meat of infected animals
- **Corrected**
- ‘Also depends on’ -> has also been associated with ‘infection… has been reported to be influenced by’ – please edit, the infection was likely not shown to be influenced.
- **Accepted and corrected**
- The sentences providing possible explanations for the higher seroprevalence in older age groups needs checking and editing. Is it reasoned to mention only oocysts?
- **Accepted and corrected**
- The discussion about cats would benefit from editing; please check that the sentences are supported by the references.
- **Accepted and corrected**
- Please rephrase the sentence using expression ‘contaminated individuals/animals’.
- **Accepted and corrected**
- Please make sure the figure legends provide enough information so that the figures can also “stand alone” without the text. E.g. the parasite and the country should be mentioned
- **Accepted and corrected**
- Was the quality of the included studies evaluated in any way? If not, why?
- **Yes, we did it.**
- Including the raw data that was extracted as supplementary material would be a very good addition.
- **Ok! Actually we uploaded it in supplementary material part.**
- Figure Files:
- **We corrected and included the files**
