# Supplementary material for: Potential risk factors associated with seropositivity for Toxoplasma gondii among pregnant women and HIV infected individuals in Ethiopia: A systematic review and meta-analysis
Source: PLoS Negl Trop Dis. 2020 Dec 15;14(12):e0008944. doi: 10.1371/journal.pntd.0008944 (PMC7771857; doi:10.1371/journal.pntd.0008944)
Supplement: Attachment — Submitted filename: PLOS Review 2.docx [file pntd.0008944.s008.docx]

Reviewer #1: This looks much better now compared to before the corrections made. However, I miss a sound justification why this systematic review was conducted in addition to those already present, which were mentioned by one of the reviewers. Within the introduction section it still reads: "no systematic studies have been conducted to determine the prevalence with associated risk factors of Toxoplasma gondii infection in pregnant women and HIV infected individuals." Once this issue is convincingly addressed, I would recommend this SLR for publication.

- **Accepted and corrected. Please have a look within the introduction part with highlight.**
